# A Multidisciplinary Approach to Unraveling the Natural Product Biosynthetic Potential of a *Streptomyces* Strain Collection Isolated from Leaf-Cutting Ants

**DOI:** 10.3390/microorganisms9112225

**Published:** 2021-10-26

**Authors:** Ana Ceniceros, Lorena Cuervo, Carmen Méndez, José A. Salas, Carlos Olano, Mónica G. Malmierca

**Affiliations:** 1 Departamento de Biología Funcional, Universidad de Oviedo, Avda. Julián Clavería s/n, 33006 Oviedo (Principado de Asturias), Spain; acenicerosm@gmail.com (A.C.); cuervolorena@uniovi.es (L.C.); cmendezf@uniovi.es (C.M.); jasalas@uniovi.es (J.A.S.); olanocarlos@uniovi.es (C.O.); 2 Instituto Universitario de Oncología del Principado de Asturias (I.U.O.P.A.), Edificio Santiago Gascón, Despacho 2.15-Campus El Cristo B, Universidad de Oviedo, 33006 Oviedo (Principado de Asturias), Spain; 3 Instituto de Investigación Sanitaria del Principado de Asturias (ISPA), Avda. del Hospital Universitario, s/n, 33011 Oviedo (Principado de Asturias), Spain

**Keywords:** *Streptomyces*, secondary metabolites, biosynthetic potential, gene clusters, bioinformatics

## Abstract

The rapid emergence of bacterial resistance to antibiotics has urged the need to find novel bioactive compounds against resistant microorganisms. For that purpose, different strategies are being followed, one of them being exploring secondary metabolite production in microorganisms from uncommon sources. In this work, we have analyzed the genome of 12 *Streptomyces* sp. strains of the CS collection isolated from the surface of leaf-cutting ants of the *Attini* tribe and compared them to four *Streptomyces* model species and *Pseudonocardia* sp. Ae150A_Ps1, which shares the ecological niche with those of the CS collection. We used a combination of phylogenetics, bioinformatics and dereplication analysis to study the biosynthetic potential of our strains. 51.5% of the biosynthetic gene clusters (BGCs) predicted by antiSMASH were unknown and over half of them were strain-specific, making this strain collection an interesting source of putative novel compounds.

## 1. Introduction

In the current scenario of emergence of new pathogens and the rapidly increasing rate of resistant bacteria to clinically used antibiotics, the need for compounds with novel bioactivities has arisen as an urgent worldwide concern [1]. According to the UK Government-commissioned O’Neill report, 10 million people a year will die by 2050 from drug resistant infections [2]. One of the main problems faced in new drug discovery is the re-isolation of already known compounds. In the period from 1940 to 1970 (known as the golden age of antibiotic discovery) the scientific community made a huge effort looking for compounds with antibiotic activity that could be used in human therapies. This led to the discovery of a great number of novel natural products such as the tetracyclines or vancomycin, but his enormous success had as a collateral effect the depletion of the traditional bioactive metabolite sources. To solve this problem, new approaches have been followed, mainly focused on the isolation of antibiotic-producing organisms from under-explored environments (e.g., extreme habitats) or new ecological niches (e.g., symbiosis with another organisms), the genetic manipulation of the known producers or the chemical modification of already known compounds [3,4].

In recent years, bioinformatics has become an essential tool in the field of new drug discovery mainly due to three facts: (i) the improvement in sequencing techniques as well as the reduction of their costs have contributed to the public availability of an enormous pool of genome sequences; (ii) the rapid development of new, powerful and ‘user-friendly’ bioinformatic tools; and (iii) the great amount of information that could be obtained without resorting to traditional expensive and time-consuming laboratory screening programs.

Insect microbiomes have attracted the attention of scientists in recent years as they comprise a valuable source of bioactive compounds [5,6]. Ants of the *Attini* tribe cultivate basidiomycete fungi in their nests to provide food to their larvae. A well-known symbiotic relationship has been stablished between these ants and bacteria of the genus *Pseudonocardia* which defends the fungus cultivar from the attack of the fungal parasite *Escovopsis* sp. by secreting antifungal compounds. In this highly competitive environment, a third partner has been identified: bacteria of the genus *Streptomyces* have been usually isolated from the cuticle of leaf-cutting ants. These Streptomycete strains form a protective microbiome as they produce many bioactive compounds (e.g., depsipeptides and polyenes) against other invasive microorganisms [7,8].

*Streptomyces* spp. have widely been recognized for their secondary metabolite production. They are, in fact, the source of 2/3 of the antibiotics currently available for human use [9]. When the first strains of this genus were sequenced, it was discovered that they can potentially produce many more compounds than those already known [10,11]. Thus, Streptomycete strains are the perfect raw material for in-depth bioinformatic analysis to unravel new biosynthetic capacities possibly hidden in their genomes.

With the aim of studying the diversity of compounds produced by an in-house group of *Streptomyces* strains isolated from leaf-cutting ants (the CS strain collection, named after Dr. Carlos Sialer, the person who collected them) [12], we performed a genome analysis of 12 of those strains and compared them to model organisms *Streptomyces coelicolor*, *Streptomyces albidoflavus*, *Streptomyces clavuligerus* and *Streptomyces arvemitillis*. We also analysed the genome of *Pseudonocardia* sp. Ae150A_Ps1, which was isolated from the same ecological niche as the *Streptomyces* CS strain collection. We generated a similarity network using BiG-SCAPE tool to detect those BGCs shared by or specific for each strain, comparing them with the MIBiG database to identify the already known gene clusters. Furthermore, we manually analysed the antiSMASH predictions to refine the results obtained from the network, and we complemented the study with data from dereplication analysis of ethyl acetate extracts from cultures of these strains grown in R5A medium. 51.5% of the gene clusters detected were unknown, and half of them were strain-specific, therefore showing the great potential that this collection of strains has for novel drug discovery and how this combination of methods can help to select gene clusters to work with.

## 2. Materials and Methods

### 2.1. Bacterial Strains and Culture Conditions

*Streptomyces* sp. CS014, CS057, CS065a, CS081a, CS090a, CS113, CS131, CS147, CS149, CS159, CS207 and CS227 strains were isolated from leaf-cutting ants (collected in Lambayeque, Peru), as previously described [12]. To obtain spore stocks, these strains were grown in mannitol-soya (MS) agar medium for 7–10 days at 30 °C and spores were scratched from the surface and stored at −80 °C in 20% glycerol. For metabolite production, spores were added to flasks containing 30 mL TSB medium and grown at 30 °C and 250 rpm. After 24 h, this seed culture was used to inoculate 50 mL of R5A medium [13] to a final OD_600nm_ = 0.2.

### 2.2. Genome Analysis

Genomes of the twelve CS strains were previously sequenced using Illumina MiSeq Sequencing technology from 2 × 300 bp insert TruSeq PCR-free library (paired-end reads) by the Department of Biochemistry at the University of Cambridge (Cambridge, United Kingdom). [12,14,15]. The sequencing reads were processed and assembled using default parameters in Newbler assembler software version 2.9. Genome annotation was performed using the NCBI Prokaryotic Genome Annotation Pipeline (https://submit.ncbi.nlm.nih.gov/subs/genome/; accessed on 25 October 2021). To verify the quality of the sequence assemblies, the genome completeness was checked with BUSCO v.5.1.3 pipeline using the lineage dataset “bacteria_odb10” (OrthoDB v.10.1) and the assessment mode “genome” [16]. Commonly used actinomycete strains were also included for comparative purposes: *Streptomyces coelicolor* A3(2) (NC_003888.3; SCP1: NC_003903.1; SCP2: NC_003904.1), *Streptomyces albidoflavus* J1074 (NC_020990.1), *Streptomyces avermitilis* MA-4680 = NBRC14893 (NC_003155.5; SAP1: NC_004719.1), *Streptomyces clavuligerus* ATCC27064 (NZ_CM000913.1; pSCL4: NZ_CM000914.1) and *Pseudonocardia* sp. Ae150A_Ps1 (NZ_MCIJ00000000.1; [17]).

### 2.3. Phylogenetic Analysis

A maximum-likelihood tree was generated by using the online tool autoMLST [18]. Genbank files containing the genomic sequences from the strains under study were used as inputs for phylogenetic inference using ‘de novo mode’ pipeline. The nearest reference organisms were selected from the NCBI RefSeq database using the MASH ANI (Average Nucleotide Identity) estimator [19]. Conserved genes with low phylogenetic noise (dN/dS value < 1) were searched in all the genomes to perform a Multi-Locus Sequence Analysis (MLSA). Among all housekeeping genes used for single copy gene screening, 82 were selected (Table S1). DNA alignments were obtained using MAFFT [20]. The final tree was inferred via a partitioned concatenated alignment of selected genes. IQ-TREE Ultrafast Bootstrap (1000 replicates) [21] and Model Finder analysis [22] were carried out.

### 2.4. BGC Prediction and Cluster Curation

Prediction of BGCs was carried out with the bioinformatic tool antiSMASH v5.2 [23], configuring the detection strictness parameter as relaxed. The KnownClusterBlast feature from antiSMASH, that compares each gene cluster with MiBiG database [24,25], was used to detect gene clusters artificially predicted as hybrids but which are instead different gene clusters. These clusters were then manually separated. In the case of the hybrid butyrolactone gene clusters, we only separated them in those cases where it was clear they belonged to different BGCs by homology to other strains that also contained these clusters but separated in the chromosome. All manually curated clusters are listed in Table S2.

### 2.5. Similarity Network Generation

The program BiG-SCAPE [26] was used to generate the secondary metabolite similarity network. Genbank files corresponding to all curated antiSMASH predicted BGCs were used as input files. BiG-SCAPE was run using the options “MIBiG” that compares all clusters with MIBiG database 1.4, “Mix” that displays all gene cluster families together, and “include_singletons” that shows all clusters that are not similar to any other in the network (singletons). Different raw distance cut-off values were also tested (0.25; 0.26; 0.27; 0.28; 0.29 and 0.3). Results were then analyzed with Cytoscape [27].

### 2.6. Metabolite Extraction and Analysis

Seven-day-old whole culture samples were extracted with an equal volume of ethyl acetate and analysed by HRMS-based dereplication against MEDINA Foundation in-house library [28] and the *Dictionary of Natural Products* version 26:2 [29] to identify already known compounds.

### 2.7. Heatmap Generation

All known BGCs predicted by homology or detected by dereplication of the strain’s cultures were represented on a heatmap. The heatmap was generated using the Python module Seaborn. A complete method was used to calculate the hierarchical clustering and the Euclidean method to calculate pairwise distances. Strains were ordered according to the phylogenetic tree.

## 3. Results and Discussion

### 3.1. DNA Sequencing and Analysis

High quality sequences are required to perform accurate and non-sesgated comparative genomic analysis. In this work, high quality genome assemblies from CS strains were used with an approximate coverage of 92% and low number of scaffolds, ranging from 2 to 9. Genome size and G + C content (%) averages were 7.8 Kb and 72%, respectively. Data regarding genome assemblies are summarized in Table 1. The benchmarking universal single-copy orthologs (BUSCO) analysis based on 124 genes validated the completeness of the genome assemblies (Figure S1).

### 3.2. Phylogenetic Analysis

To locate specific niche selection of bacterial communities, we carried out a phylogenetic analysis. 16S RNA locus has been traditionally used to group bacteria into operational taxonomic units (OTUs), but lately some inaccuracies have been reported using this method due to horizontal transfer events and variable rates of evolution. Nowadays, a broader spectrum analysis based on several housekeeping genes, not only on 16S RNA, has been stablished as the method of choice [30,31].

**Figure 1:**
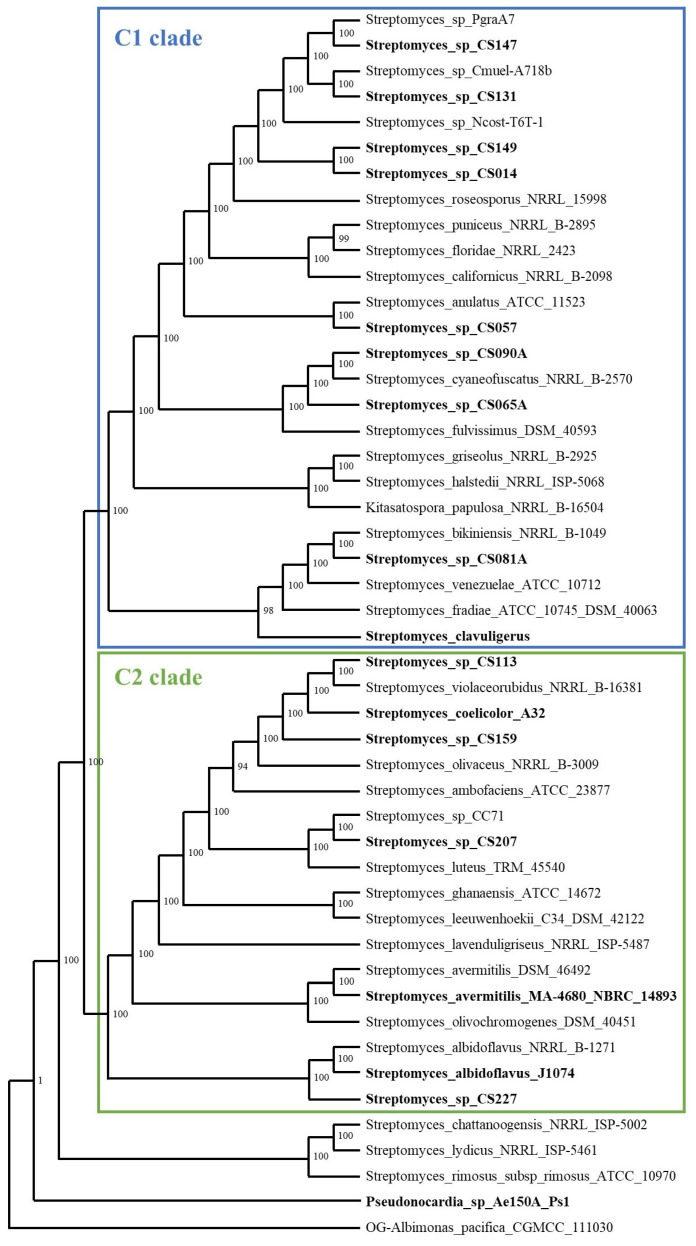
Phylogenetic tree of the concatenated nucleotide sequences of 82 housekeeping genes generated with autoMLST. Strains used in this work are highlighted in bold.

We performed a multi-locus sequence analysis (MLSA) based on 82 housekeeping genes, including the twelve CS strains under study, four *Streptomyces* strains generally used as reference organisms, one *Pseudonocardia* strain that shared the ecological niche with the CS strains and some closely related soil bacteria selected by the online tool autoMLST (Figure 1). Surprisingly, the CS strains did not cluster together and were spread across the phylogenetic tree. These results could suggest these CS isolates were ubiquitous to the surrounding soil and randomly colonized the ants, so they were not specific to the leaf-cutting ant nests. Similarly, the association between Actinobacteria and fungus-farming termites is more likely to be due to an unspecific event rather than a real symbiotic relationship [32].

Two different clades could be inferred from the tree: C1 included most of the CS strains and the reference strain *S. clavuligerus*, and C2 that comprised CS113, CS159, CS207 and CS227 strains together with the well-known reference strains *S. coelicolor*, *S. avermitilis* and *S. albidoflavus*. According to this data, strains from the C1 clade are potentially more attractive candidates for new drug discovery screenings since they were less related to *Streptomyces* strains commonly used for secondary metabolite analysis (Figure 1).

### 3.3. AntiSMASH Prediction of Secondary Metabolite BGCs

AntiSMASH alone is a powerful tool to predict secondary metabolite gene clusters in bacterial genomes. The KnownClusterBlast functionality, included in antiSMASH, compares all predicted gene clusters with the MIBiG database, which is extremely useful to identify already described BGCs. AntiSMASH, however, does not always correctly delimit gene clusters, resulting in a lower similarity with its corresponding MIBiG entry. On occasions, two or more gene clusters are predicted to be as one large hybrid gene cluster, as it is the case of candicidin and antimycin, which are generally predicted to be a single PKS/NRPS/lanthipeptide/T3PKS cluster. Only when entering the “KnowClusterBlast” tab in antiSMASH results display, the cluster is divided showing one part with 100% similarity to the MIBiG entry for candicidin (BGC0000034) and another part with 100% homology with the MIBiG entry to antimycin (BGC0000958), clearly showing that they are two completely different BGCs. Thus, a manual curing of the results was needed to improve gene cluster delimitation. A total of 36 BGCs were manually curated (Table S2).

**Figure 2:**
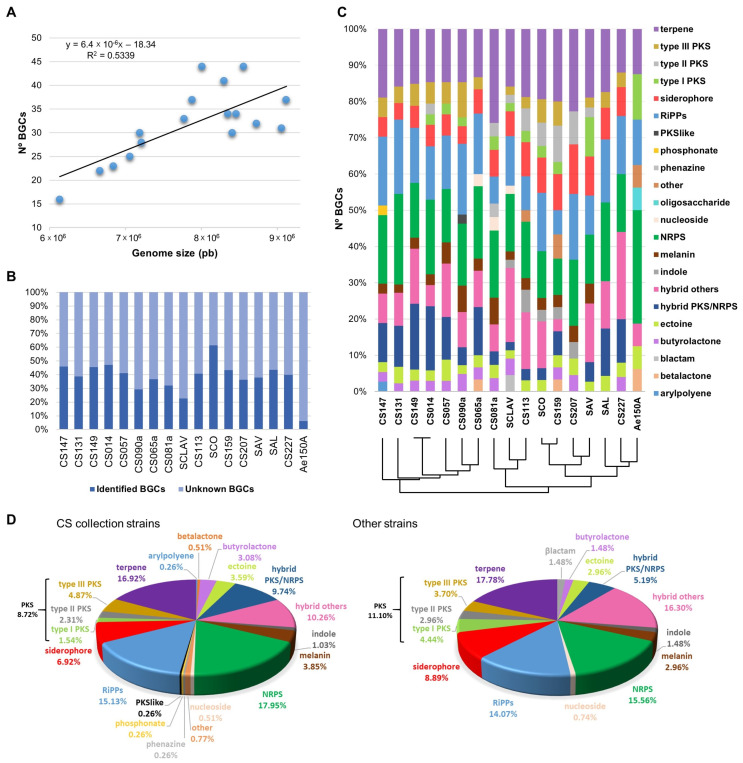
Analysis of the BGCs detected in the 17 strains under study. (**A**) Relationship between genome size and the number of BGCs per genome. (**B**) Frequency of unknown BGCs versus identified BGCs per genome. (**C**) Inter-strain distribution of the 22 major classes of BGCs. (**D**) BGC type distribution among the ‘CS collection group” and the rest of the reference Streptomycete strains.

AntiSMASH analysis based on homology to known BGCs deposited in the MIBiG database (and after curation of artificially classified hybrid clusters) identified a total of 541 BGCs in the 17 strains under study (Tables S3–S19), distributed in 22 major classes. These bacterial strains harbored between 16–44 BGCs per genome (mean = 31.82; s.d. = 7.66). Interestingly, a moderate trend was observed between genome size and the number of BGCs per strain (Figure 2A), with the largest genomes usually containing more BGCs. We stablished a threshold of ≥85% similarity (based on antiSMASH analysis) to identify a BGC as involved in the biosynthesis of the predicted compound. We chose a rather conservative threshold to limit the number falsely identified BGCs. According to this, all genomes showed more than 50% of BGCs related to putatively novel compounds (mean = 61.87%; s.d. = 11.67), being the only exception *S. coelicolor* A3(2) (38.71%; Figure 2B). The long lasting and extensive research carried out on this model strain would be the reason behind this deep knowledge about its biosynthetic potential. On the contrary, in the less studied *Pseudonocardia* strain only 6.25% of its BGCs were linked to a described compound. Regarding the distribution of each BGC type per genome (Figure 2C), a common pattern can be described in general terms, being hybrid compounds, non-ribosomal peptides (NRPs), terpenes, RiPPs (comprising bacteriocins, lanthipeptides, lassopeptides and linear azol(in)e-containing peptides (LAPs)) and polyketides (PKs) the most representative types of BGCs. Noticeable, *Pseudonocardia* showed the most different BGC distribution, which is in accordance with the phylogenetic results that placed this strain outside the common group of Streptomycetes. Within this group, CS081a, CS113 and CS159 strains stood out as the most different ones, with lower percentages of hybrid compounds and PKS BGCs.

To identify a possible relationship between the ecological niche and secondary metabolite production profile, we studied the Streptomycete strains clustered into two different groups: one comprising the twelve CS strains and the other with the rest of the reference strains (Figure 2D). In the CS group, 20 BGC classes were detected being the most represented hybrid compounds (38 BGCs for hybrids PKS/NRPS and 40 for other hybrids), NRPSs (70 BGCs), terpenes (66 BGCs), RiPPs (59 BGCs) and PKSs (six, nine and 19 BGCs for type I, type II and type III PKS, respectively). We found a similar pattern analysing the ‘reference strain group’. Interestingly, the CS strains were enriched in BGC types as arylpolyene, β-lactone, other, phenazine, phosphonate and PKS like classes, which were only present within this group. The only BGC type exclusive of the ‘other strains’ group was β-lactam, corresponding to the clavulanic acid/cephamycin D cluster of *S. clavuligerus*.

From the total of 390 BGCs detected in the CS strains, 155 were predicted as already known gene clusters (homology ≥ 85%), 33 presented a moderate homology (50–84%, suggesting likely similar biosynthetic function) and 201 were putatively novel BGCs without significative homology to any BGC in the MIBiG repository (Tables S3–S19). These data indicated that 51.5% of the predicted BGCs coded for putatively unknown compounds. Together with the higher diversity of BGC type described above, these results pointed out the great potential of these CS strains for new drug discovery studies.

From the 155 known BGCs detected by antiSMASH in the 17 genomes used in this study, the production of 60 different already described compounds (e.g., desferrioxamine B and isorenieratene) could be inferred. Among them, 16 BGCs were shared by the CSs and the reference strains, 18 were exclusive of the reference strains and 26 were unique in the CS collection. Notably, most of the shared BGCs were classified as siderophores, terpenes, butyrolactones, ectoine and melanin, and the great majority of the exclusive BGCs fell into the PKS, NRPS and RiPP classes. This scenario is similar to that described by Murphy and co-workers [32], suggesting the idea of an ancient actinomycete carrying BGCs involved in the biosynthesis of metabolites with crucial roles in the establishment and survival in soil environments (e.g., photoprotectants as terpenes, inter-, intra-species signaling compounds such as butyrolactones and stress resistance ones like ectoine or siderophores). The ability to produce “more specialized” compounds (PKS, NRPS, RiPPs, nucleosides, etc.) could be acquired during several evolutionary events to fulfil new challenging conditions found in more specific niches.

### 3.4. Secondary Metabolite Analysis

**Figure 3:**
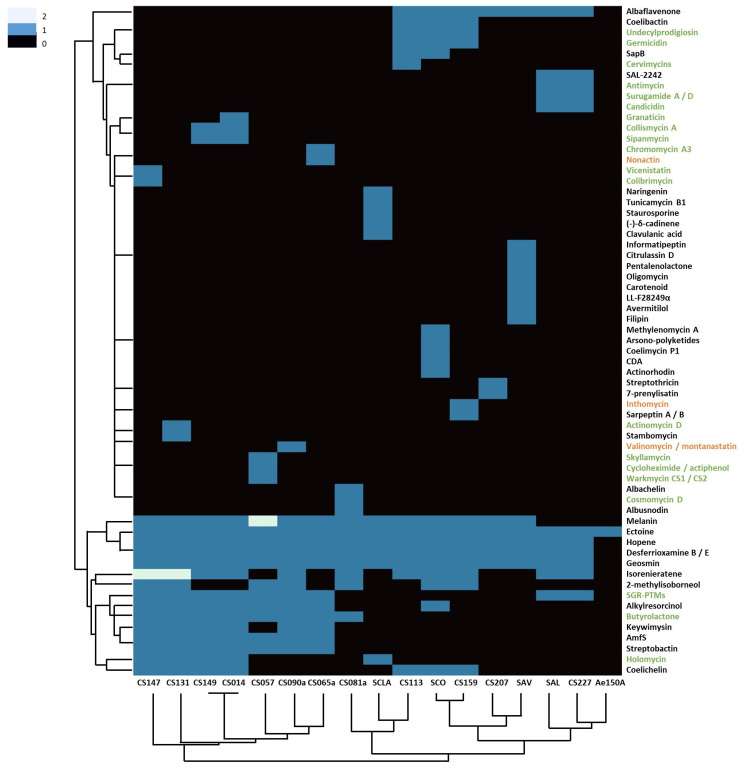
Heatmap of compounds produced by the strains used in this work. Coloured scale represents the number of times a BGC is present in the genome. These data include compounds detected by dereplication (orange), predicted by genetic homology ≥85% of BGCs according to antiSMASH v5.2 (black) or both (green).

In order to complement the results obtained by antiSMASH prediction, we created a heatmap containing all BGCs that have a predicted homology over 85% to a known BGC and metabolites that had been detected by dereplication (Figure 3).

**Figure 4:**
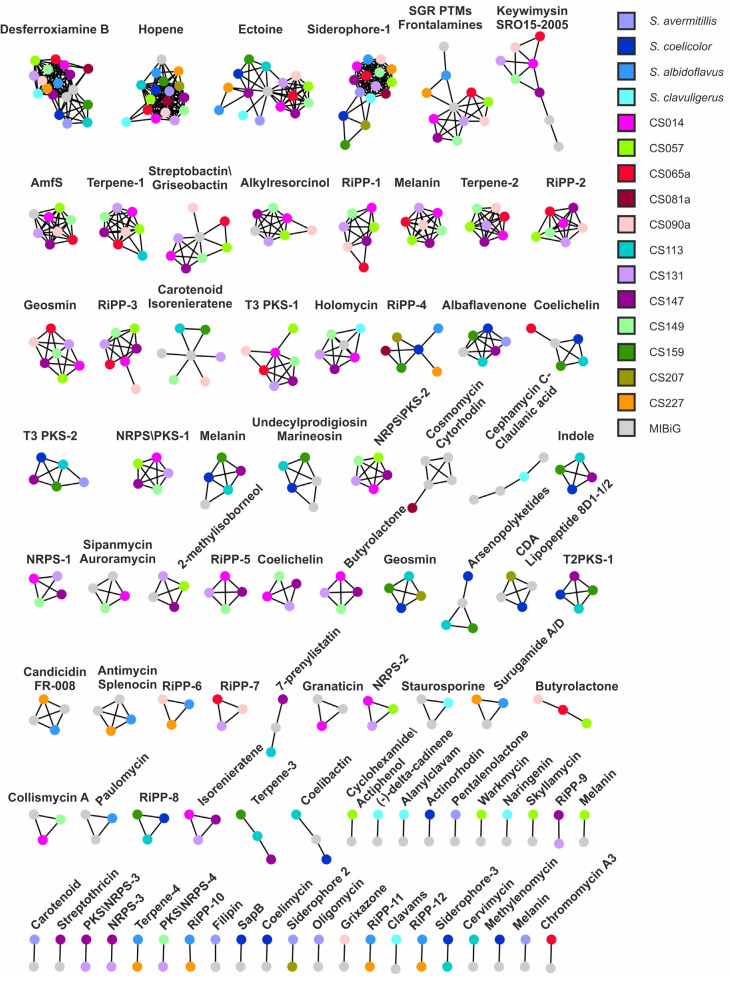
Similarity network generated with all secondary metabolite clusters detected by antiSMASH after manual curation. Strains are represented by dots of different colors.

Four BGCs were found in every *Streptomyces* strain, those being ectoine, hopene, desferrioxamine and geosmin, possibly due to their vital function in highly competitive soil environments and preventing osmotic and nutrient stresses (Figure 3). Melanin was only absent in *S. albidoflavus* and CS227 Streptomycete strains, which is in accordance with their characteristic non-pigmented colonies. Interestingly, some strains seemed to contain two copies of the same BGC: melanin (in strain CS057) and isorenieratene (in CS131 and CS147 strains), both presumably involved in photoprotection. Surprisingly, *Pseudonocardia* sp. only shared the ectoine BGC with the rest of the strains, although it is phylogenetically related and shares the ecological niche with the CS isolates. Moreover, this ectoine BGC does not cluster together with the *Streptomyces* ones in the similarity network (Figure 4).

The fact that, based on bioinformatic analysis, only one of the 16 BGCs present across the *Pseudonocardia* sp. genome could be linked to its biosynthetic product might be explained by the little knowledge accumulated about the biosynthetic potential of *Pseudonocardia* sp. (compared to *Streptomyces* sp.)

With the aim of obtaining a more visual representation of shared and specie-specific BGCs, we generated a similarity network using the program BiG-SCAPE, which bound every BGC (represented by nodes) to others that have high similarity and to its corresponding MIBiG BGC entry (Figure 4). Different distance cut-off values were tested to find the best conditions so that gene clusters that may have high similarity but produce different metabolites (such as NRPS or PKSs), were not all clustered together in one or few group of nodes. We also verified that clusters responsible for the biosynthesis of the same product but that may have slight differences in their arrangement were not scattered throughout the network. We selected a raw distance cut-off of 0.29, considering that the standard value (0.3) was creating groups of nodes consisting of only of MIBiG clusters (Figure 4). Most of the known gene clusters were bound to one or more MIBiG nodes in the similarity network, facilitating their identification. The network was also key to identify gene clusters improperly assigned as hybrids since they were clearly visible as two groups of separated nodes bound by one hybrid node which contains both BGCs classes. When these hybrid nodes were further studied in the antiSMASH environment, it was clear that they had been wrongfully predicted as hybrids. Thus, this kind of similarity networks could represent a good complement to the antiSMASH analysis to gain a deeper insight into the biosynthetic potential of a given strain.

Surprisingly, six different node groups only included the same seven CS strains, CS014, CS057, CS065a, CS090a, CS131, CS147 and CS149. These groups of nodes corresponded to the BGCs of AmfS, griseobactin, RiPP-1, terpene-2, RiPP-2 and RiPP-3. These seven strains are close related according to the phylogenetic tree (Figure 1) which could explain why they share many BGCs.

Only nodes corresponding to desferroxiamine and hopene BGCs were present in all *Streptomyces* strains. Ectoine and geosmin BGCs were also detected as present in all *Streptomyces* strains in the antiSMASH analysis previously reported in this work (Figure 3) but oddly they were not grouped all together. In some cases, BGCs identified by antiSMASH include not related surrounding genes. For example, regarding geosmin, where only the terpene cyclase is needed for the biosynthesis of the compound [33], BGCs were grouped in the network depending on the similarity of neighbouring genes, creating an artefact in the similarity network that resulted in the impossibility of grouping all these BGCs in one single node group. In the case of the ectoine node cluster, only *Streptomyces* CS081A and *Pseudonocardia* sp. were misplaced, even though they showed 100% homology to ectoine BGC [34]. Ectoine BGC surrounding genes are quite conserved in all *Streptomyces* strains included in this work, except in CS081A. The same happens with the ectoine BGC from *Pseudonocardia* sp. therefore indicating that this was the reason why these two clusters have not been properly grouped with the rest.

Similarly, the melanin BGC, which is present in every strain except *Streptomyces albidoflavus*, *Streptomyces* sp. CS227 and *Pseudonocardia* sp., was also scattered throughout the network. They were bound to different melanin MIBiG entries, which were introduced together with other adjacent genes. Therefore, each BGC was bound to the MIBiG entry that has more similar neighbouring genes, showing the importance of delimiting the best as possible the BGCs uploaded into MIBiG database.

On the other hand, hopene BGCs were grouped in one single node group (Figure 4). In most strains, the hopene gene cluster showed similarity lower than 85% in antiSMASH results, which based on the criteria applied on this work, it would mean that most of the strains did not contain this BGC. However, in this case, the gene cluster and neighbouring genes were much conserved and, therefore, they were all grouped together in the network. All of them contained a squalene-hopene cyclase, essential for the synthesis of hopene [35], therefore we considered this BGC to be present even though antiSMASH similarities are quite low.

Another interesting observation was that the BGC for γ-butyrolactone is usually predicted to cluster together with other BGCs. γ-Butyrolactones are quorum-sensing molecules that have been described in different strains of *Streptomyces* [36]. These molecules are known to regulate secondary metabolism in *Streptomyces* [37,38,39,40] and its biosynthetic genes are widely extended throughout this genus [36]. In the case of coelimycin gene cluster, genes for γ-butyrolactone biosynthesis are located and included within the coelimycin BGC, since they participate in its regulation [41,42,43,44,45]. Thus, this BGC was not contemplated in our heatmap as butyrolactone BGC when predicted to be linked to another cluster. These clusters were included in the hybrid-other category.

An additional advantage obtained by the similarity network analysis was that we were able to identify which gene clusters were present in only one strain and from those, which ones do not correspond to any described compound. Interestingly, 52.7% of all unknown gene clusters were strain-specific (singletons), which represented a total of 106 BGCs (data not included in Figure 4 for clarity purposes; a graphical representation of each singleton is available in Figures S2–S13). One of the biggest setbacks faced when looking for novel metabolites is rediscovering compounds that had previously been described in other strains [46]. Singletons are, therefore, excellent targets for mining the occurrence of new compounds, since they do not seem to be spread across the *Streptomyces* genus and therefore lowering the risk of rediscovering already known compounds. Most of the singletons found in the CS strains were larger than 20 Kb and contained many genes, facts that may be indicative of their true biosynthetic potential. Only 12 singletons (marked with an asterisk in Tables S3–S19) were shorter than 20 Kb thus they should be treated carefully because they could be fragmented BGCs leading to false hits. Interestingly, the most abundant gene cluster family (GCF) in all singletons is NRPS, comprising 30 of the 106 singletons. NRPSs are the source of important bioactive compounds such as penicillins, daptomycin, cyclosporins or bleomycin [47]. NRPSs synthetize peptides independently from ribosomes and can use more than 500 substrates, hence, allowing a wide variety of structures [48] and making this GCF an important niche for the discovery of novel bioactive compounds. These 30 strain-specific NRPS clusters are therefore a promising target for further studies.

### 3.5. Correlation between BGCs Detected by antiSMASH Analysis and Dereplication Results

A total of 24 already known compounds were detected in samples extracted with ethyl acetate from whole cultures of CS strains grown in R5A medium (Table S20). Interestingly, all of them (except the γ-butyrolactone SCB) were polyketides and/or non-ribosomal peptides confirming the R5A as a highly-valuable culture medium to produce this kind of compounds. A few more metabolites were detected during dereplication assays but they did not produce any positive matches in the databases, making them very good candidates for new drug discovery. Thus more in-depth analysis is in progress attempting to link these compounds to their BGC so we could characterize their biosynthetic pathways.

Among the 42 known different metabolites potentially produced by the CS strains (according to antiSMASH), 21 were detected and identified by dereplication. The remaining 21 compounds that were not detected by dereplication were mainly classified as siderophores, terpenes, lanthipeptides, lasso peptides, lipopeptides, ectoine, butyrolactone and melanin. Our inability to detect these compounds could be due to two different reasons: (i) they were not being produced under our laboratory conditions or (ii) the extraction method using ethyl acetate is not suitable for this type of metabolites, and other solvents (e.g., *n*-butanol or ethanol) should be used instead [49,50,51,52].

Interestingly, three compounds were detected by dereplication but not predicted by antiSMASH: the oxazole-containing inthomycin polyketides, the atypical nonactin polyketides and the non-ribosomal peptide valinomycin. Here we faced three different scenarios that could explain these discrepancies. The BGC involved in inthomycin biosynthesis was recently described in *Streptomyces* sp. strain SYP-A7193 [53] but, as far as we were concerned, it was not deposited in the MIBiG repository (or it is not yet accessible) thus it could not be predicted. That fact pointed out the importance of making publicly accessible the data about new described BGCs for the new drug discovery field. The only NRPS-transatPKS BGC predicted in CS159 might be responsible for inthomycin production based on the homology of the core genes and in the 20% similarity to the related phthoxazolin BGC (MIBiG accession BGC0001740; [54]). Nonactins comprise a group of compounds synthetized by an atypical type II PKS system (with two ketoacyl synthases and two ketoreductases but lacking the corresponding acyl carrier protein). Probably due to this special configuration, antiSMASH was not able to predict the putative cluster responsible for their biosynthesis. As described by Matarrita-Carranza and co-workers [55], the nonactin BGC could only be detected using the antiSMASH loose strictness parameter. If we ran an antiSMASH analysis set with this new configuration, we were able to find a BGC classified as fatty acid that retrieved an 100% similarity to the macrotetrolide BGC (BGC0000243) and 57% to the nonactin BGC (BGC0000252). As nonactins are the parent compounds of macrotetrolides [56] and both clusters were usually found together [55], we could assign this BGC as the responsible for the nonactin production detected by dereplication in CS065a culture samples. Finally, mining through the CS090a genome sequence we were only able to find one NRPS type BGC with moderate similarity (56%) to the valinomycin/montanastatin BGC (BGC0001846; [57]) and to the valinomycin BGC (BGC0000453; [58]). In-depth analysis of the described valinomycin gene cluster and a literature revision pointed out that only two genes within the cluster (both encoding NRPSs) were necessary to synthetize the compound and that another thioesterase coding gene improved its production [59,60]. Homologues of these three “core” genes were found within the putative BGC predicted by antiSMASH in the CS090a genome thus we could link this BGC with the valinomycin production in this Streptomycete strain.

As a result of the analysis performed in this work, we were able to identify the strains CS081a and CS090a as the most promising candidates within the CS collection to continue the search on novel bioactive compounds. These strains belong to clade C1, which, as mentioned before, only contains one of the model strains used in this study (*S. clavuligerus*) (Figure 1) and harbor in their genomes more singletons than the rest of the strains analyzed (15 and 18, respectively). Also, over half of these singletons belong to the NRPS, PKS or hybrid NRPS/PKS BGC classes, which are well known as sources of bioactive compounds [61,62].

## 4. Conclusions

Sometimes it is difficult to prioritize the best strains or BGCs candidates in new drug discovery. Here, we highlighted the importance of a careful bioinformatic study at the first stages of secondary metabolite studies based on genome mining. We proposed the combination of genome analysis by antiSMASH, the generation of a similarity network, phylogenetic analysis and sample dereplication as a useful approach to find the secondary metabolite wealth of our CS strain collection. We were able to predict known and unknown BGCs as well as the frequency of appearance in the genomes, and a way to identify which gene clusters could be the better candidates for new drug discovery. We were also able to identify two strains (CS081a and CS090a) that showed the best potential for novel bioactive compound discovery within the CS collection.

## Figures and Tables

**Table 1:** Genomic assembly data summary.

Strain	Size (Mb)	% G + C	No. Scaffold	Scaffold N50	Scaffold L50	No. Contig	Contig N50	Contig L50	Genome Coverage	No. Coding Genes	No. RNA	GenBank Accesssion
CS014	8.46	71.5	5	7,805,634	1	67	314,402	9	127.85x	7178	70	QBHV00000000.1
CS057	8.35	71.5	2	8,333,859	1	48	318,741	8	265.32x	6938	78	NEVF00000000.1
CS065a	7.19	71.4	2	6,921,137	1	34	377,645	6	540.60x	6016	70	QBHW00000000.1
CS081a	7.21	71.6	9	1,199,078	3	19	595,322	5	114.86x	6206	75	QBHX00000000.1
CS090a	8.30	71.7	8	7,527,415	1	53	323,608	8	372.20x	7098	73	QBHY00000000.1
CS113	8.73	73.1	3	8,695,358	1	28	823,625	4	78.17x	7558	79	NEVC00000000.1
CS131	8.01	72.3	4	7,968,990	1	26	805,396	4	88.88x	6697	81	QBHZ00000000.1
CS147	7.88	71.6	2	7,866,616	1	39	444,639	7	101.71x	6753	75	QBIA00000000.1
CS149	7.77	71.6	2	7,754,255	1	80	224,771	12	556.87x	6629	80	PVZY00000000.1
CS159	8.41	72.2	6	8,122,180	1	75	275,233	11	252.62x	7301	85	NEVD00000000.1
CS207	6.66	72.6	4	4,214,913	1	22	532,902	4	177.89x	5774	73	QBIB00000000.1
CS227	7.06	73.4	5	6,794,013	1	50	548,106	5	370.61x	5695	84	NEVE01000000.1

## Data Availability

The data presented in this study are available in the article and the Supplementary Material. All genome sequences were deposited at NCBI’s GenBank database (see Table 1).

## Supplementary Materials

The following are available online at https://www.mdpi.com/article/10.3390/microorganisms9112225/s1: Figure S1: Validation of genome completeness of the 12 CS strains using BUSCO tool; Figures S2–S13: graphical representation of singletons present in each CS strain. Table S1: Housekeeping genes used in MLSA analysis; Table S2: Curated gene clusters; Tables S3–S19: BGC prediction by antiSMASH v.5.2 in *Streptomyces* sp. CS strains; Table S20: Compounds detected by dereplication.
