# Supplementary material for: A Multidisciplinary Approach to Unraveling the Natural Product Biosynthetic Potential of a Streptomyces Strain Collection Isolated from Leaf-Cutting Ants
Source: Microorganisms. 2021 Oct 26;9(11):2225. doi: 10.3390/microorganisms9112225 (PMC8621525; doi:10.3390/microorganisms9112225)
Supplement: Supplementary file 1 [file microorganisms-09-02225-s001.zip › Table S20. Dereplication.pdf]

Table S20. Compounds detected by dereplication.

Family of compounds	Type of metabolite	Producer strain
Actinomycin	NRP	CS131
Actiphenol*	Polyketide	CS057
Antimycin	Hybrid NRP-polyketide	CS227
Candicidin	Type I polyketide - glycosylated	CS227
Cervimycin	Type II polyketide - glycosylated	CS113
Colibrimycin	NRP	CS147
Chromomycin	Type II polyketide - glycosylated	CS065a
Cycloheximide*	Polyketide	CS057
Collismycin	Hybrid NRP / polyketide	CS014, CS149
Cosmomycin	Type II polyketide - glycosylated	CS081a
Germicidin	Type III polyketide	CS113
Granaticin	Type II polyketide	CS014
Holomycin	NRP	CS014, CS147, CS149
Inthomycin	Hybrid NRP / polyketide	CS159
Nonactin	Polyketide	CS065a
SCBs	Butyrolactone	CS131
SGR-PTMs	Hybrid NRP / polyketide	CS057, CS065a, CS090a, CS147, CS149, CS227
Sipanmycin	Type I polyketide - glycosylated	CS014, CS149
Skyllamycin	Hybrid NRP / polyketide	CS057
Surugamide	NRP	CS227
Undecylprodigiosin	Hybrid NRP / polyketide	CS113, CS159
Valinomycin	NRP	CS090a
Vicenistatin	Type I polyketide - glycosylated	CS147
Warkmycin	Type II polyketide - glycosylated	CS057

*The biosynthesis of both compounds is directed by the same BGC.
