# Supplementary material for: A Multidisciplinary Approach to Unraveling the Natural Product Biosynthetic Potential of a Streptomyces Strain Collection Isolated from Leaf-Cutting Ants
Source: Microorganisms. 2021 Oct 26;9(11):2225. doi: 10.3390/microorganisms9112225 (PMC8621525; doi:10.3390/microorganisms9112225)
Supplement: Supplementary file 1 [file microorganisms-09-02225-s001.zip › Figures S2-S13. Singletons.pdf]

Singletons – CS014

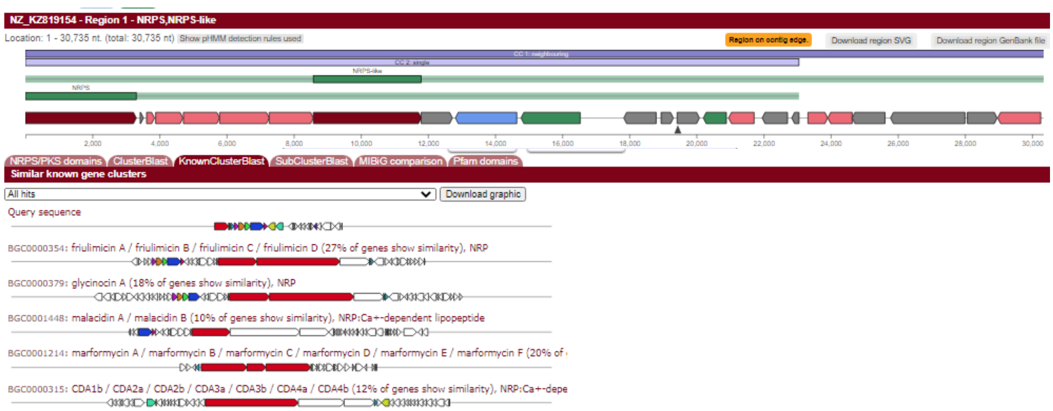

14.1

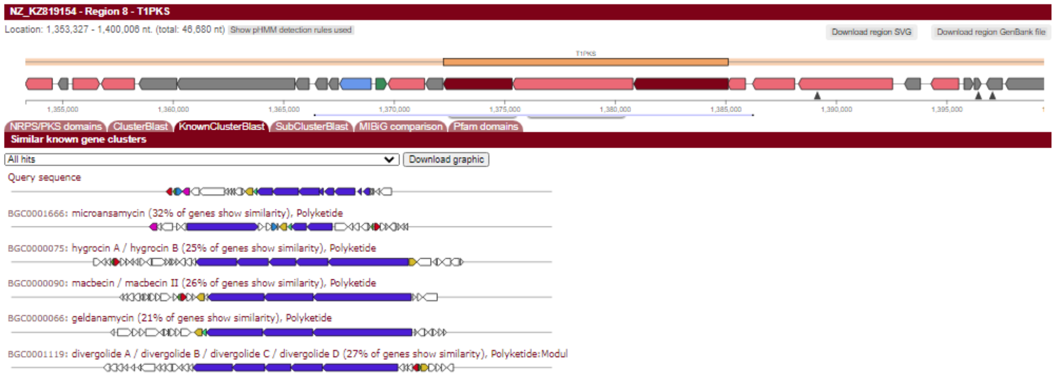

14.8

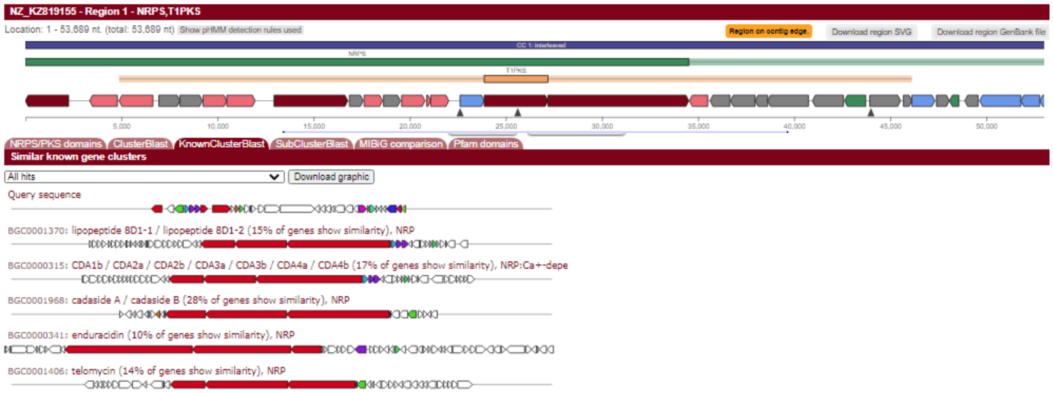

14.26

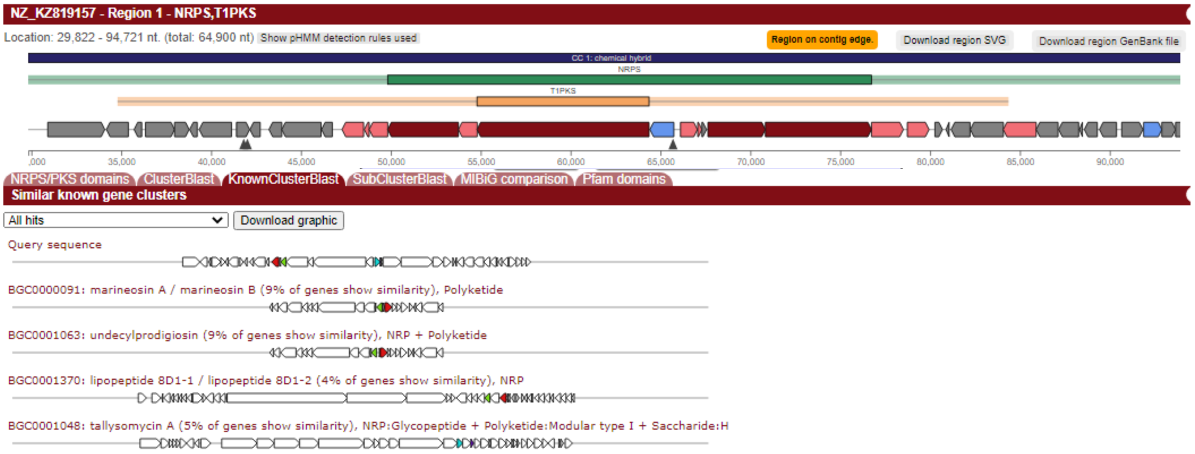

14.31

14.32

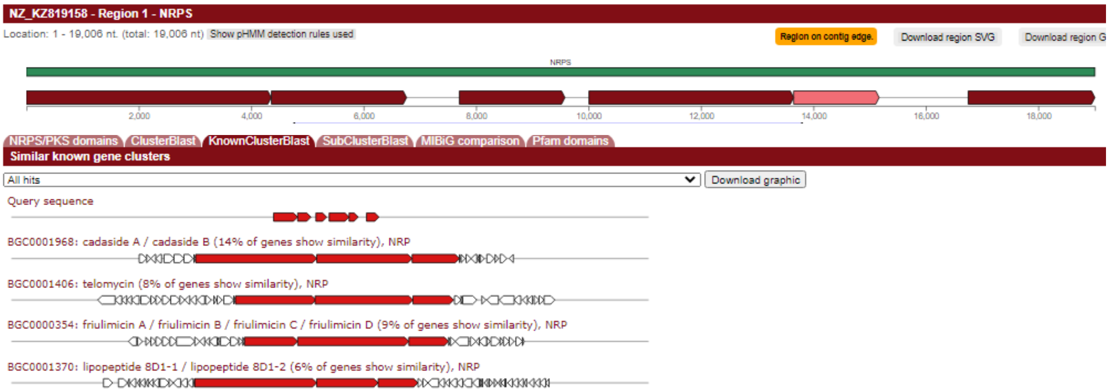

Figure S2. Genetic organization of BGCs classified as singletons in CS014. The KnownClusterBlast results (if any) are included for comparative purposes. Data obtained from antiSMASH v.5.2.

Singletons – CS057

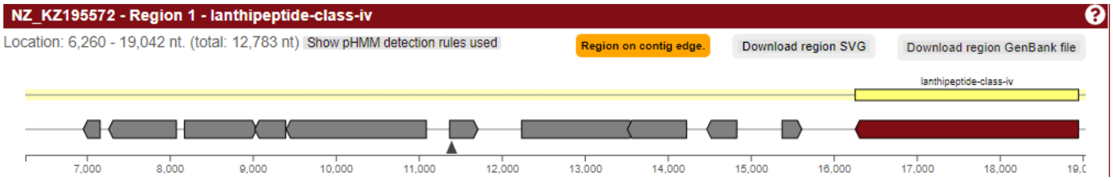

57.2a

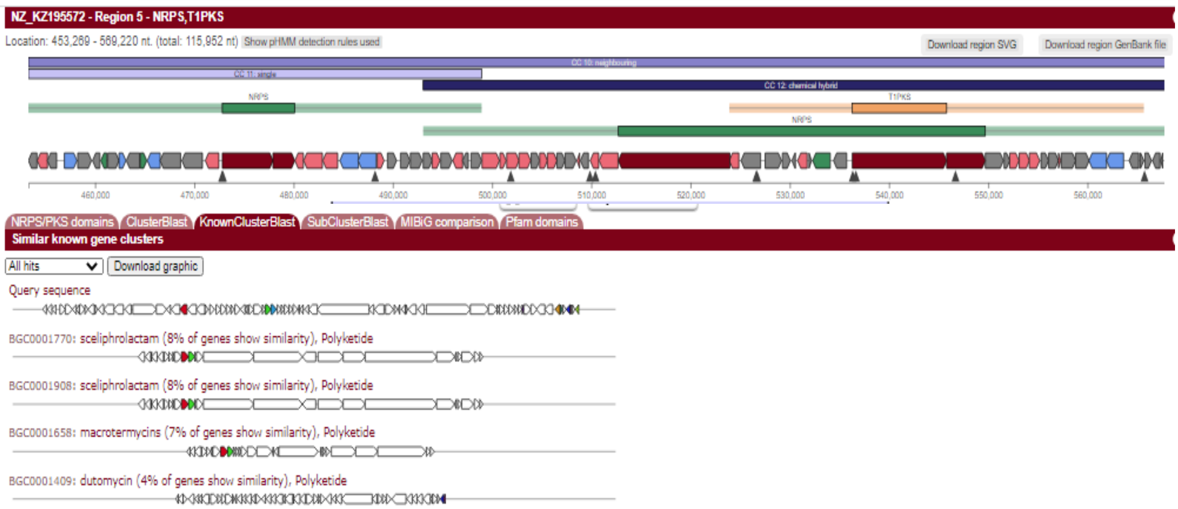

57.5

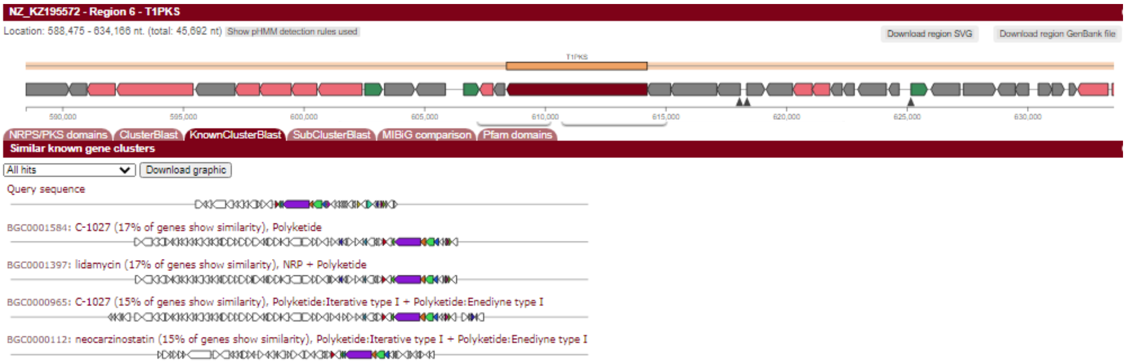

57.6

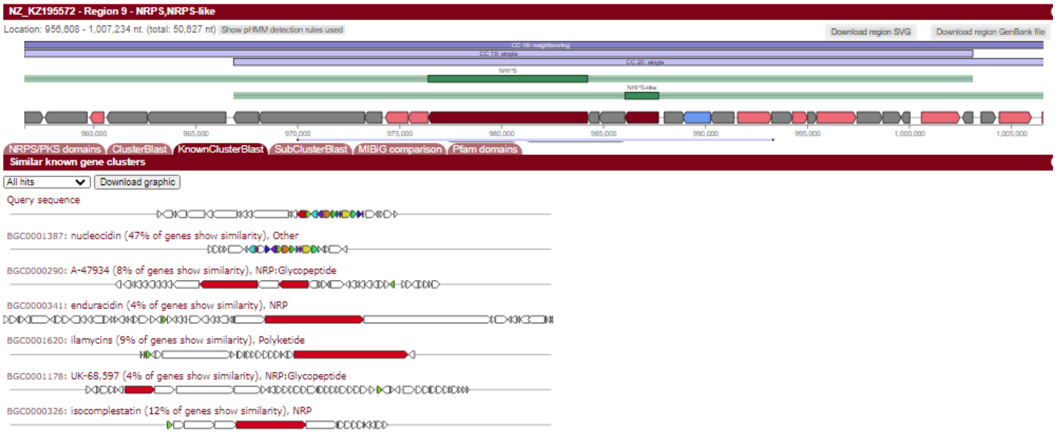

57.9

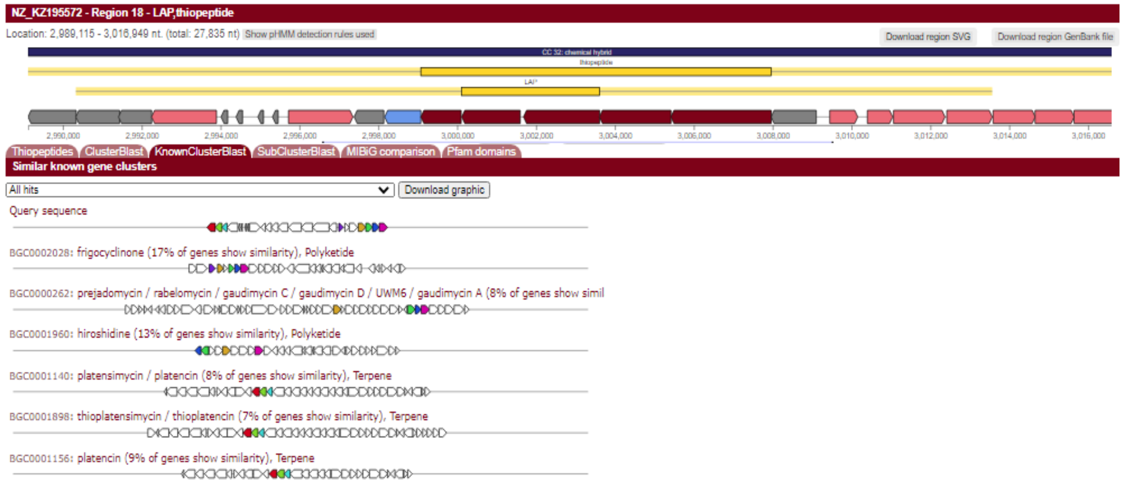

57.18

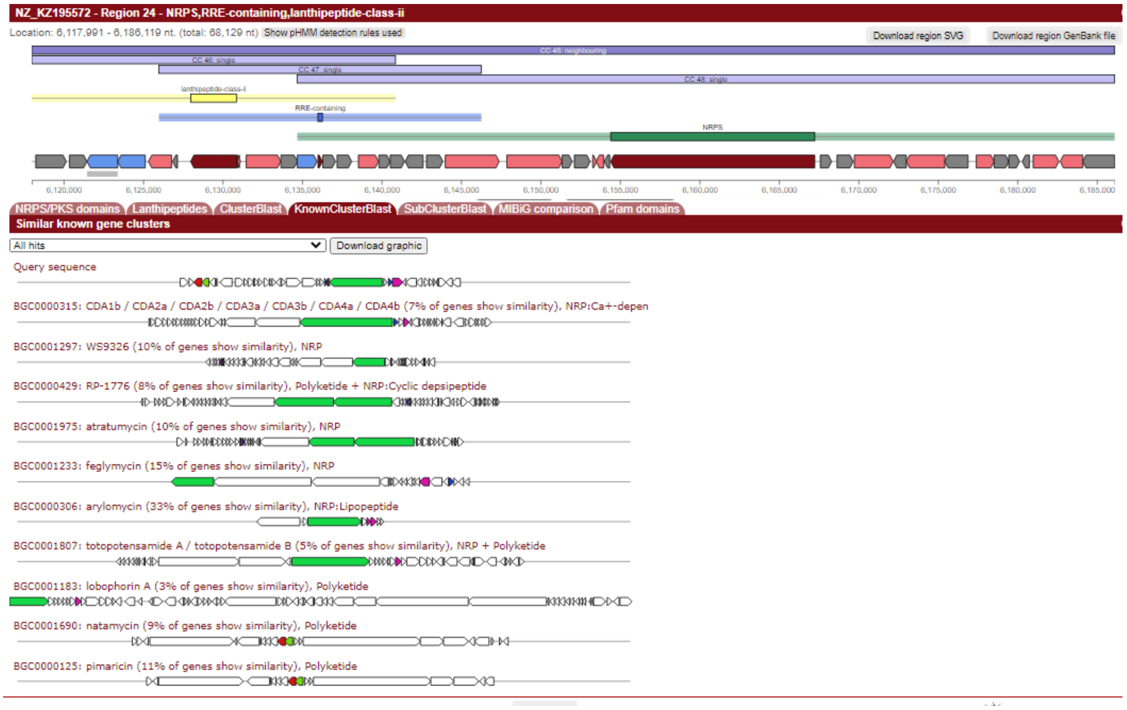

57.24

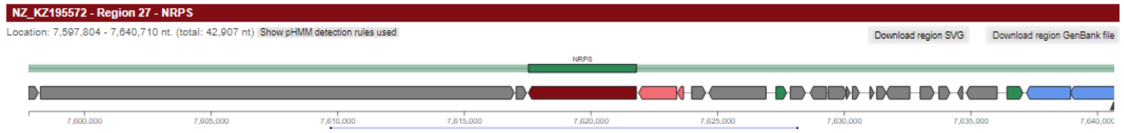

57.27

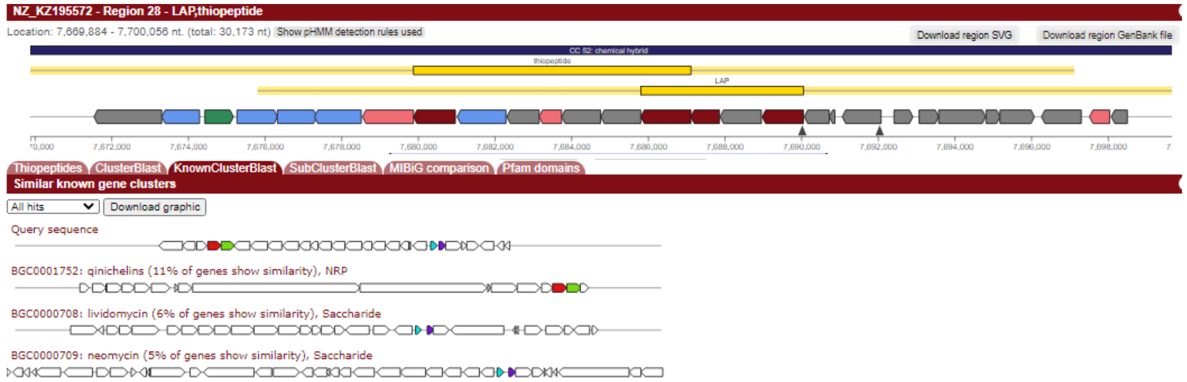

57.28

Figure S3. Genetic organization of BGCs classified as singletons in CS057. The KnownClusterBlast results (if any) are included for comparative purposes. Data obtained from antiSMASH v.5.2.

Singletons – CS065a

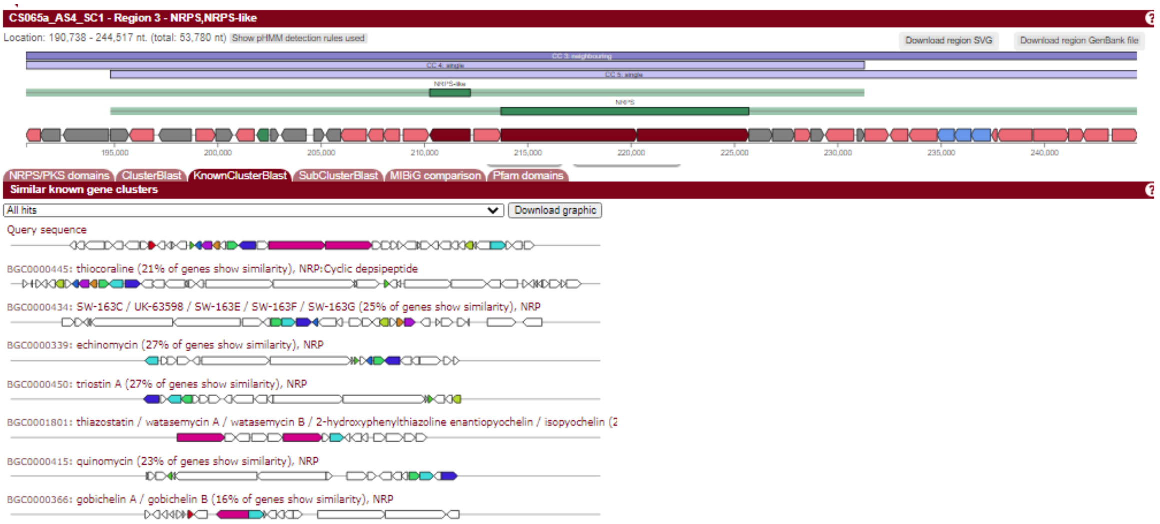

65a.3

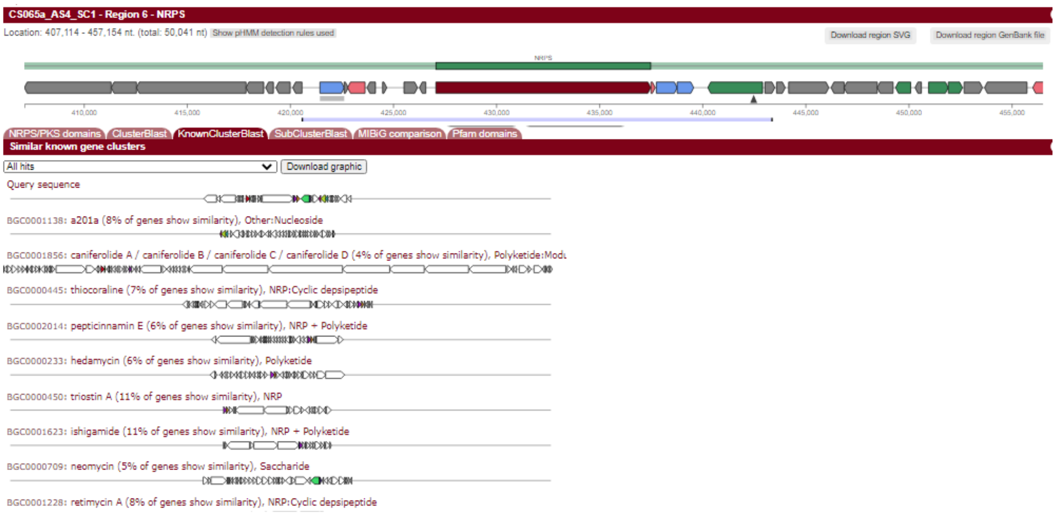

65a.6

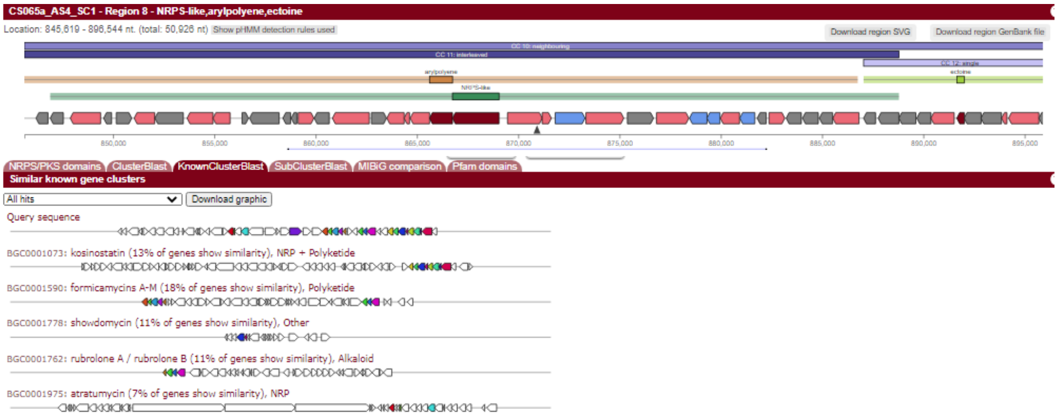

65a.8

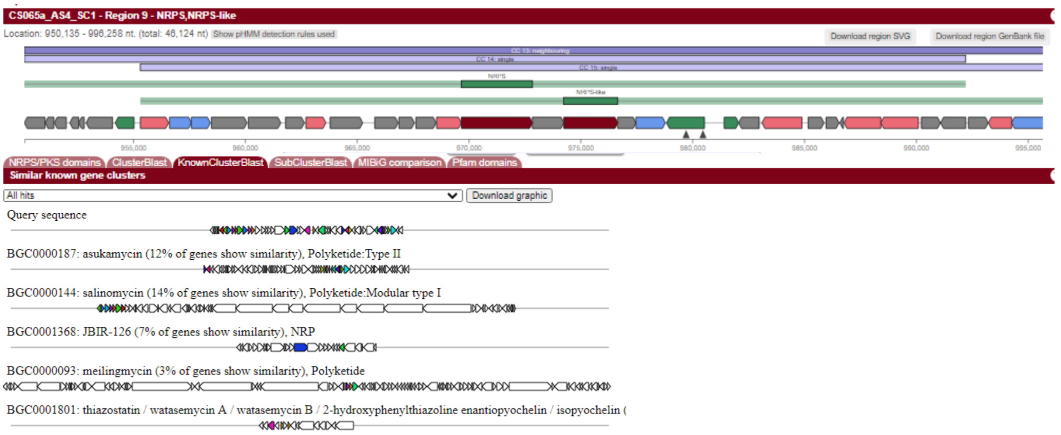

65a.9

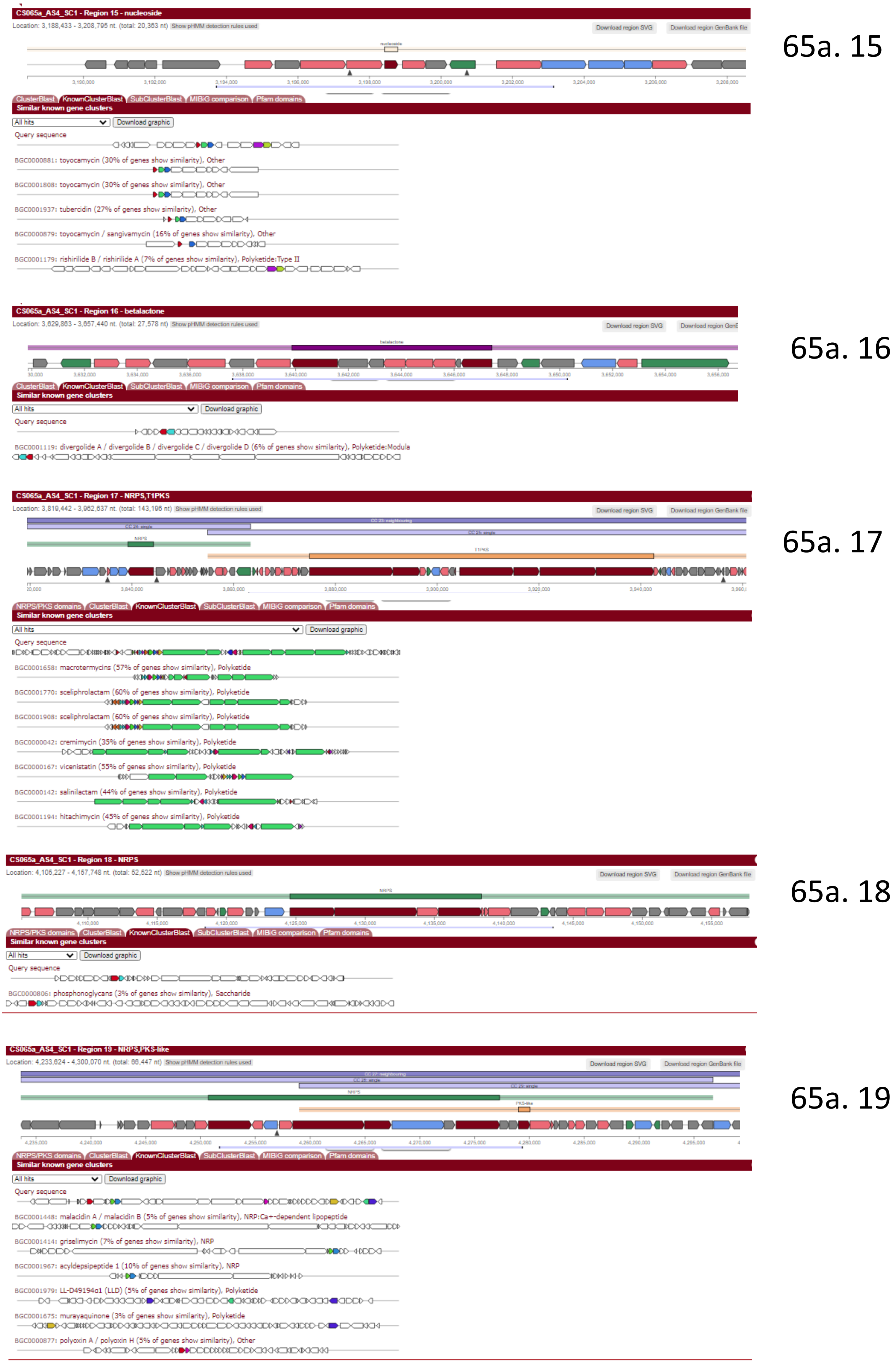

Figure S4. Genetic organization of BGCs classified as singletons in CS065a. The KnownClusterBlast results (if any) are included for comparative purposes. Data obtained from antiSMASH v.5.2.

Singletons – CS081a

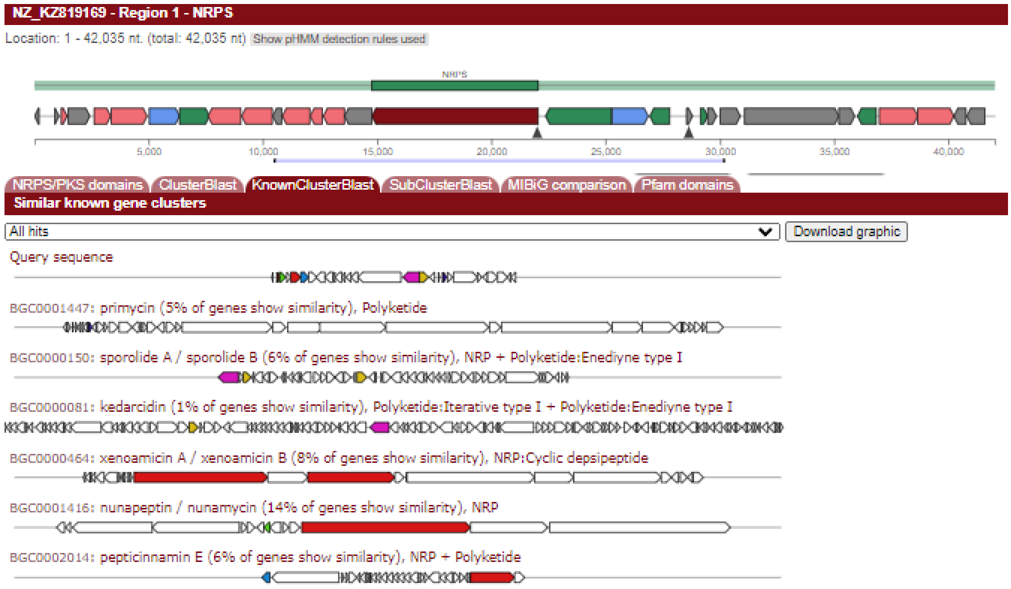

81a. 4b

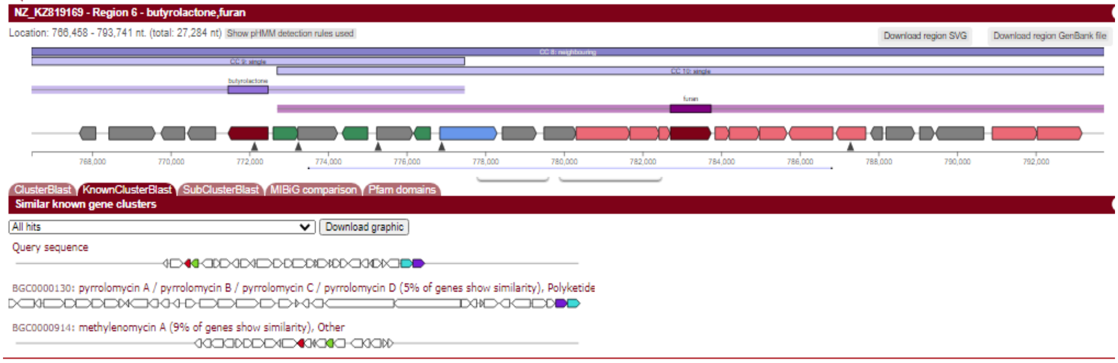

81a. 6

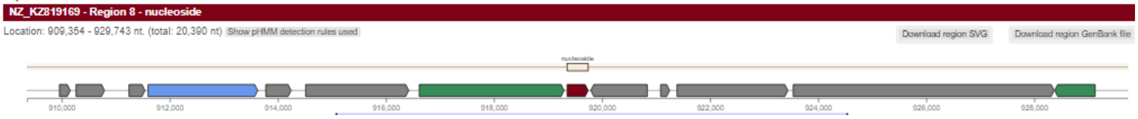

81a. 8

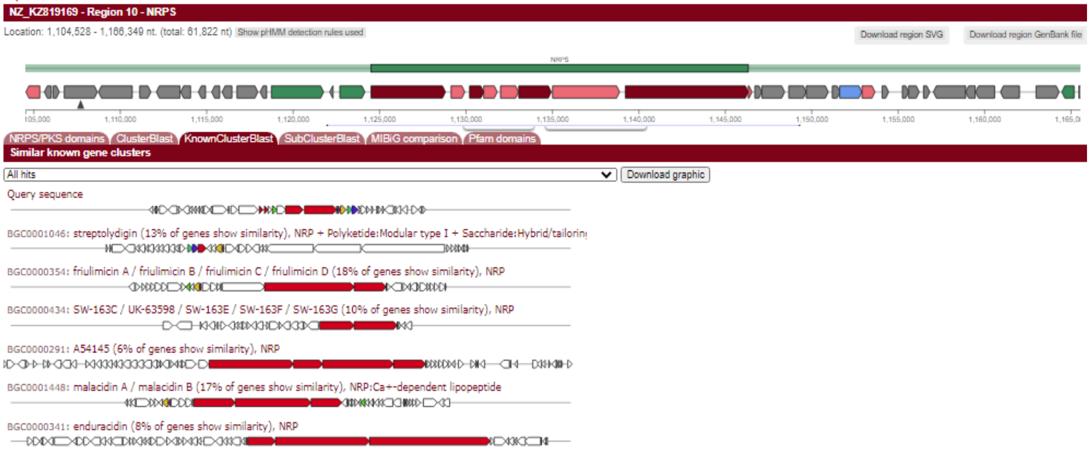

81a. 10

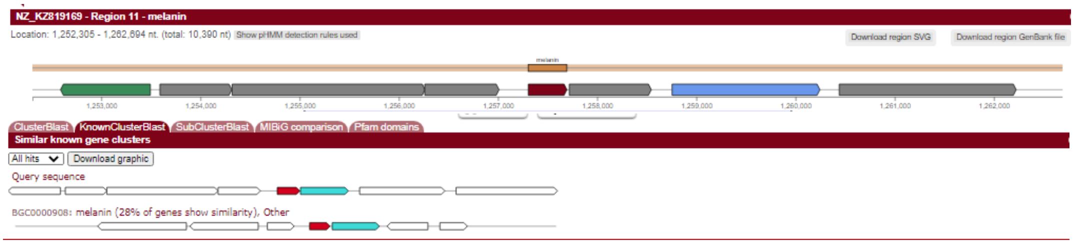

81a. 11

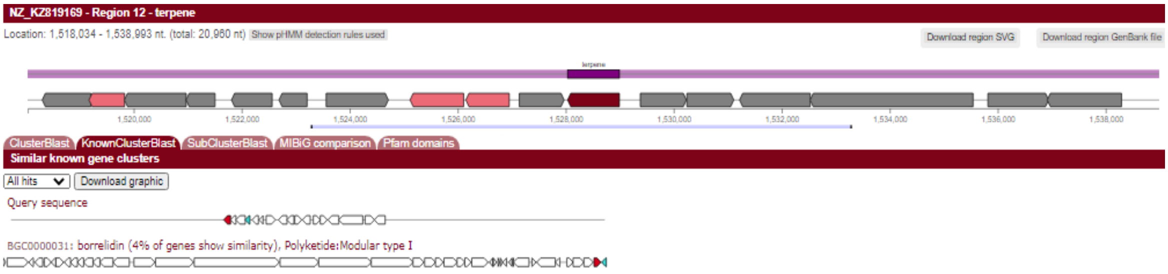

81a.12

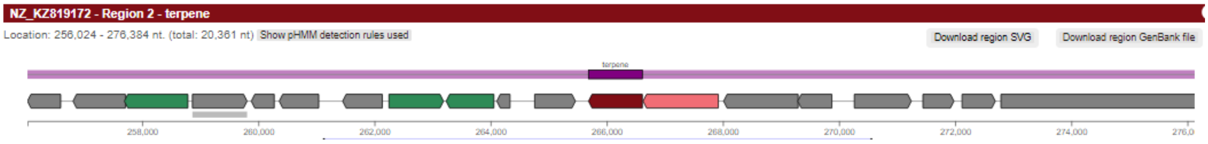

81a. 16

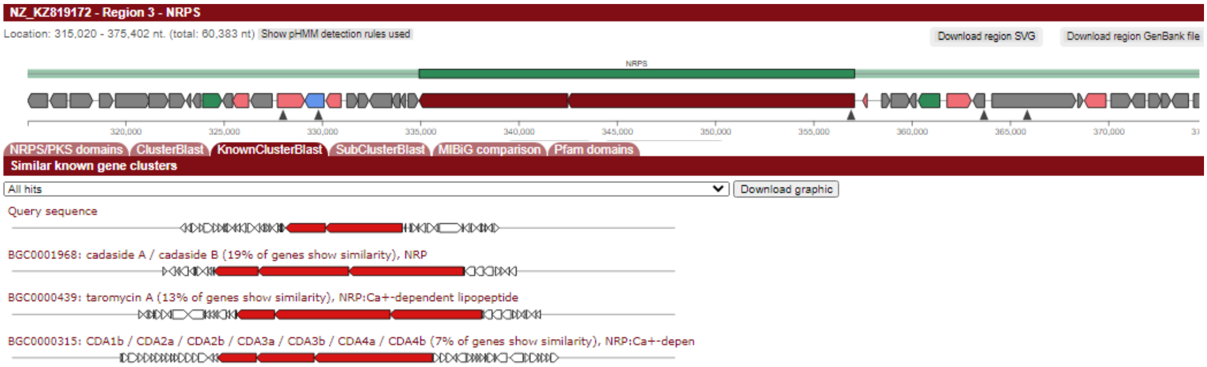

81a. 17

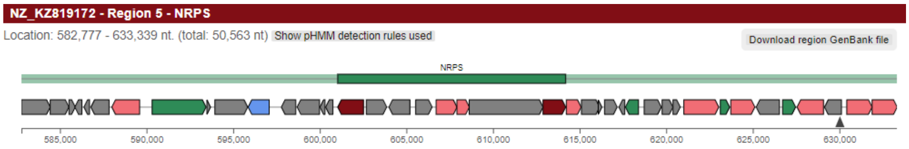

81a. 20

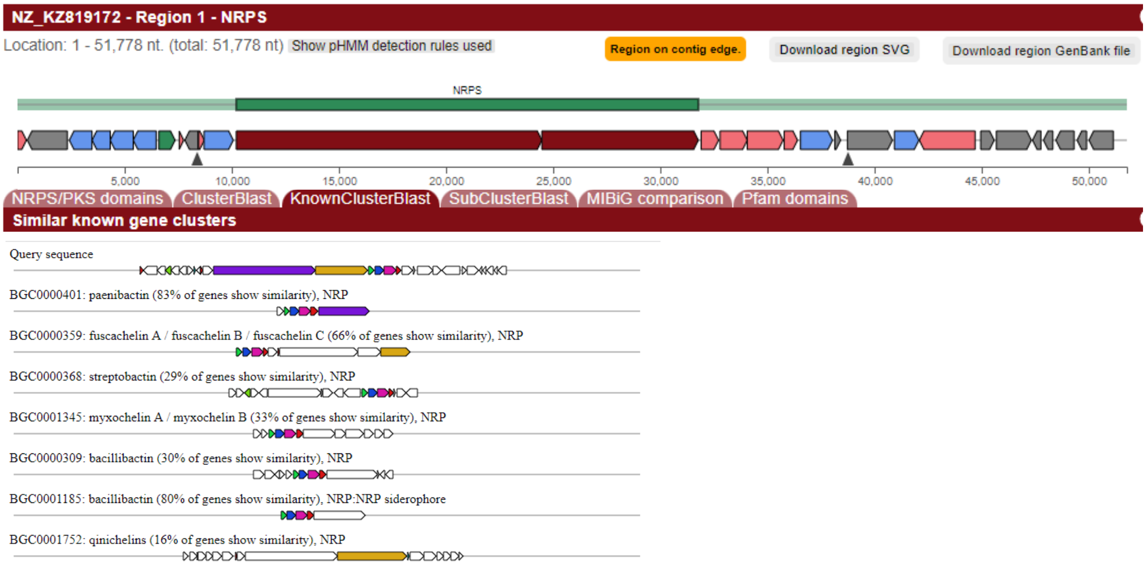

81a. 21b

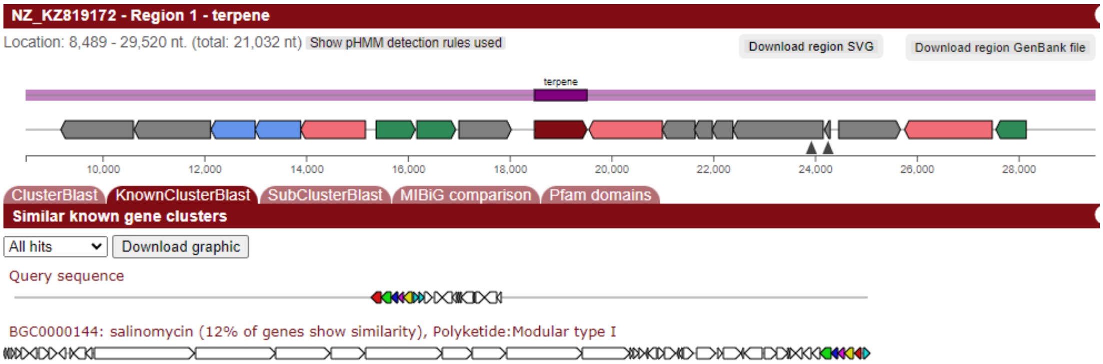

81a. 22a

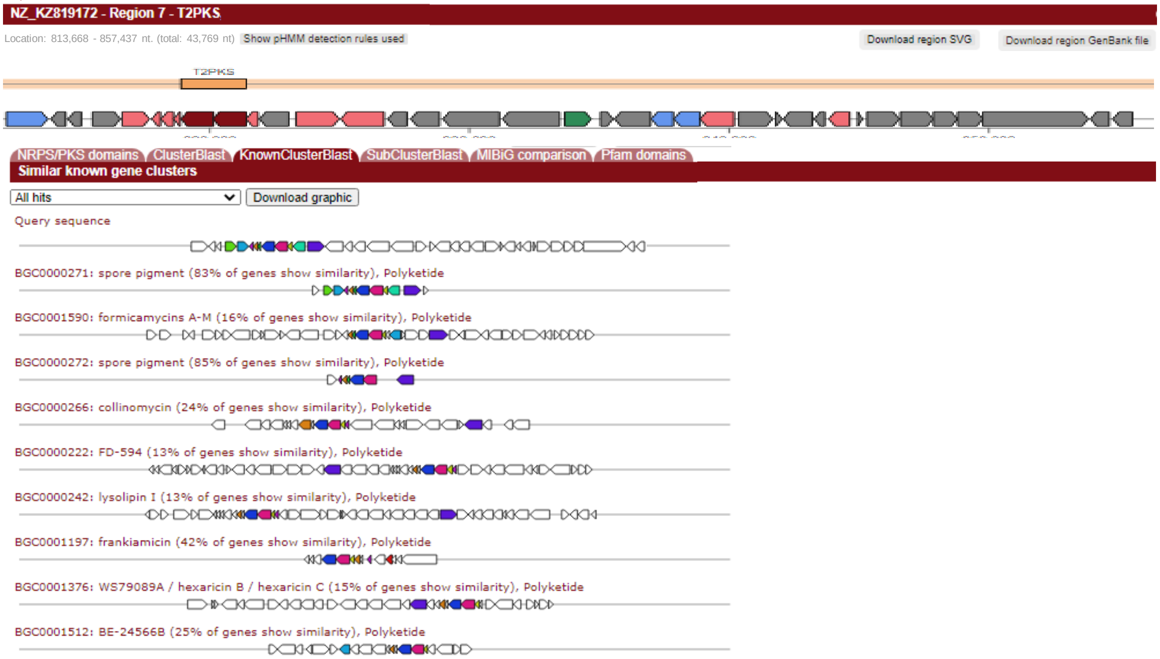

81a. 22b

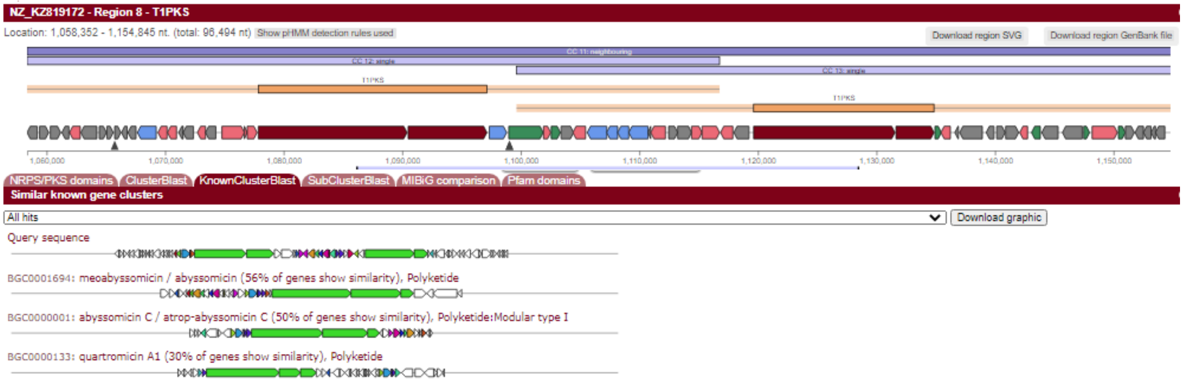

81a. 23

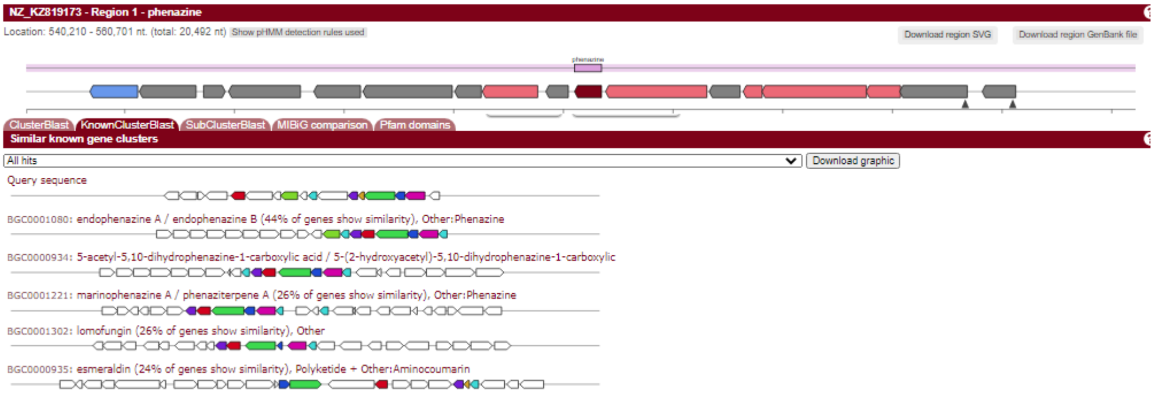

81a. 24

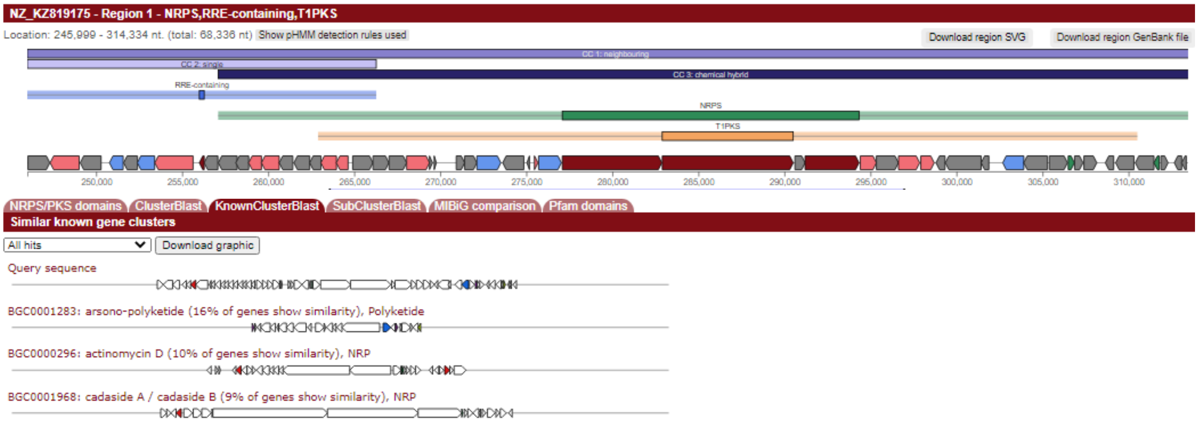

81a. 25

Figure S5. Genetic organization of BGCs classified as singletons in CS081a. The KnownClusterBlast results (if any) are included for comparative purposes. Data obtained from antiSMASH v.5.2.

Singletons - CS090a

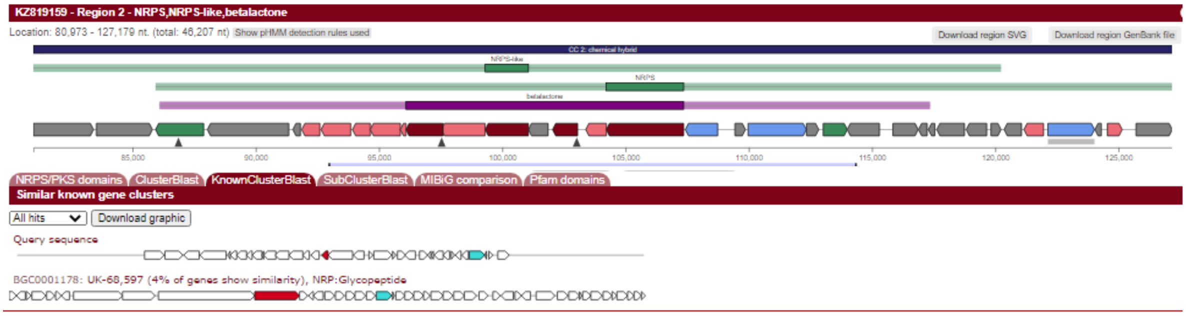

90a.1b

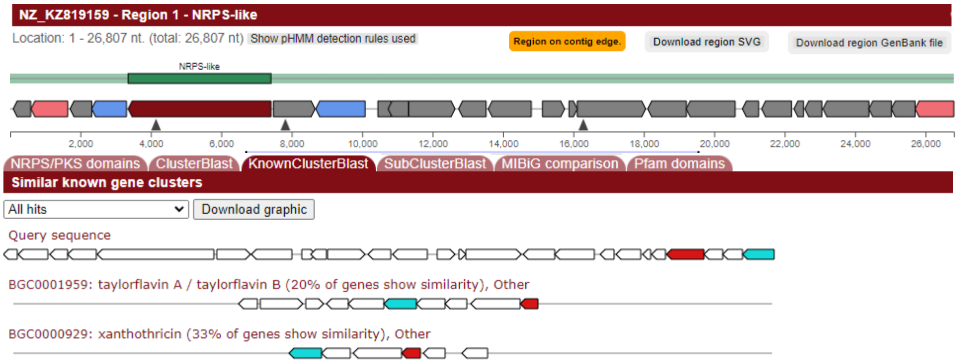

90a.2b

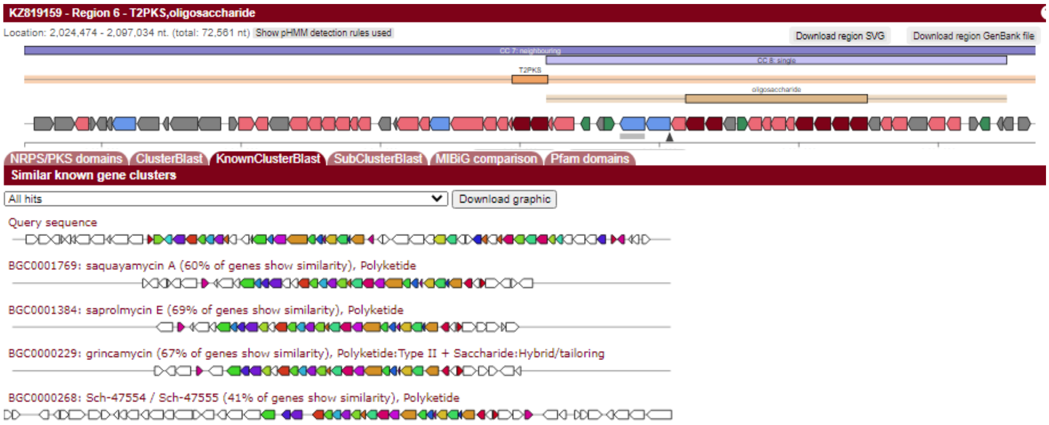

90a.5

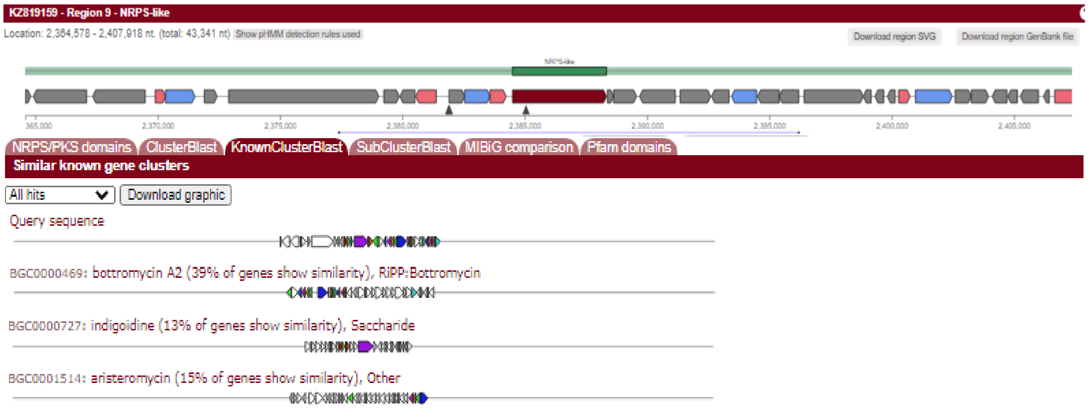

90a.8

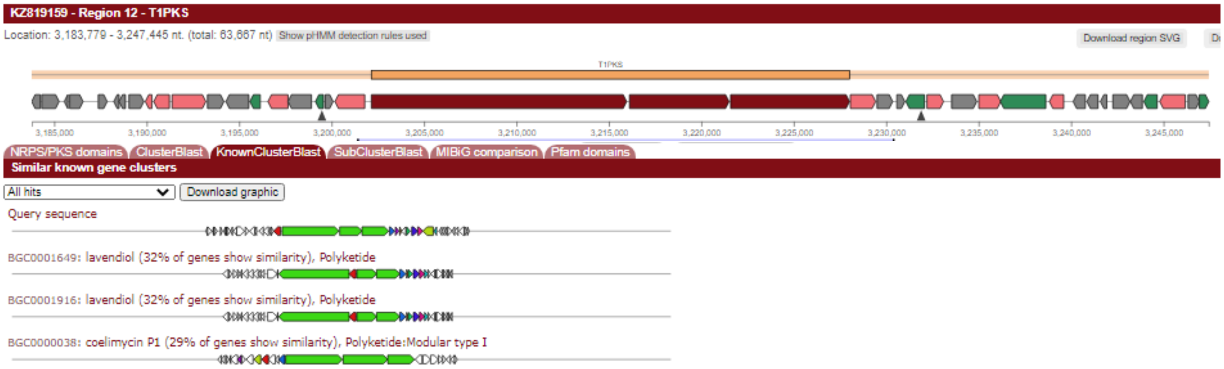

90a.11

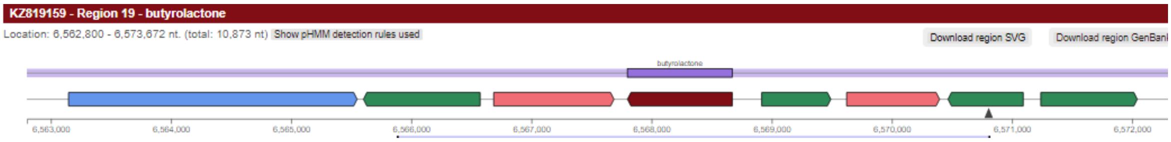

90a.18

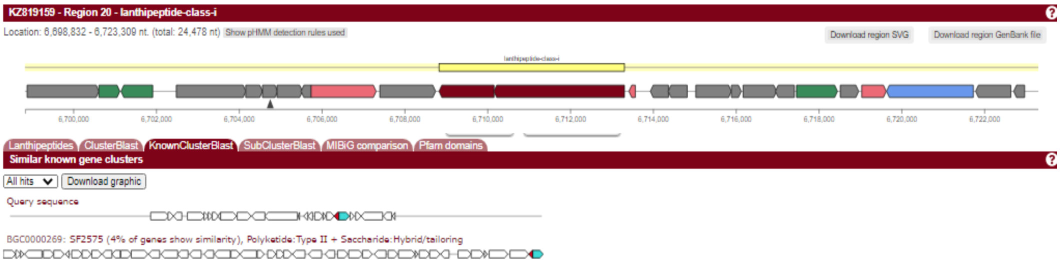

90a.19

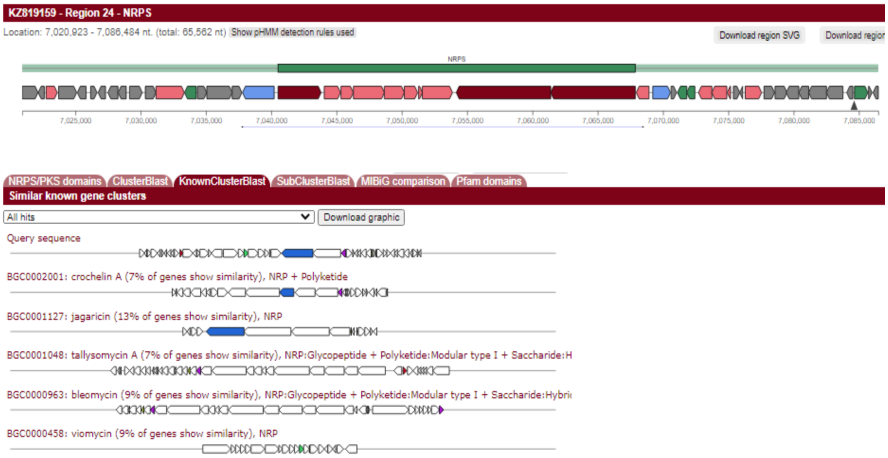

90a.23

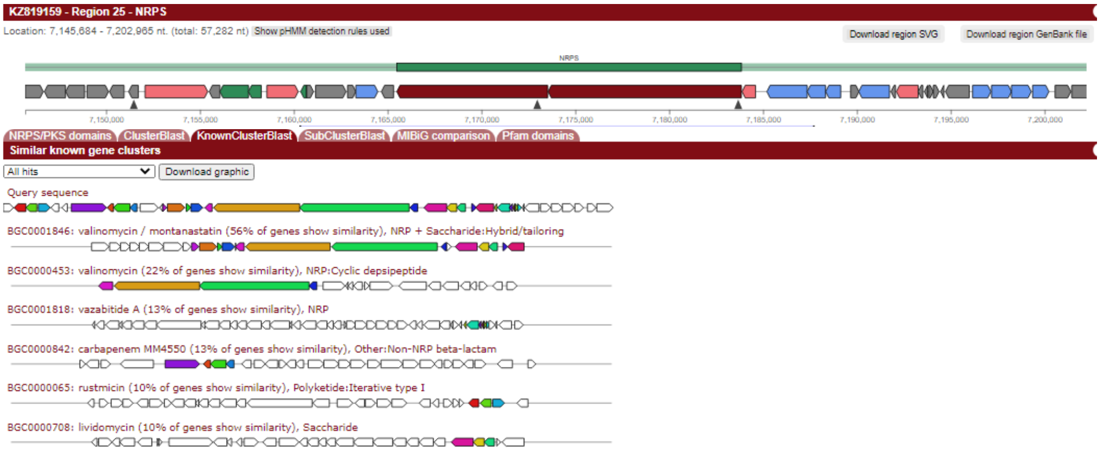

90a.24

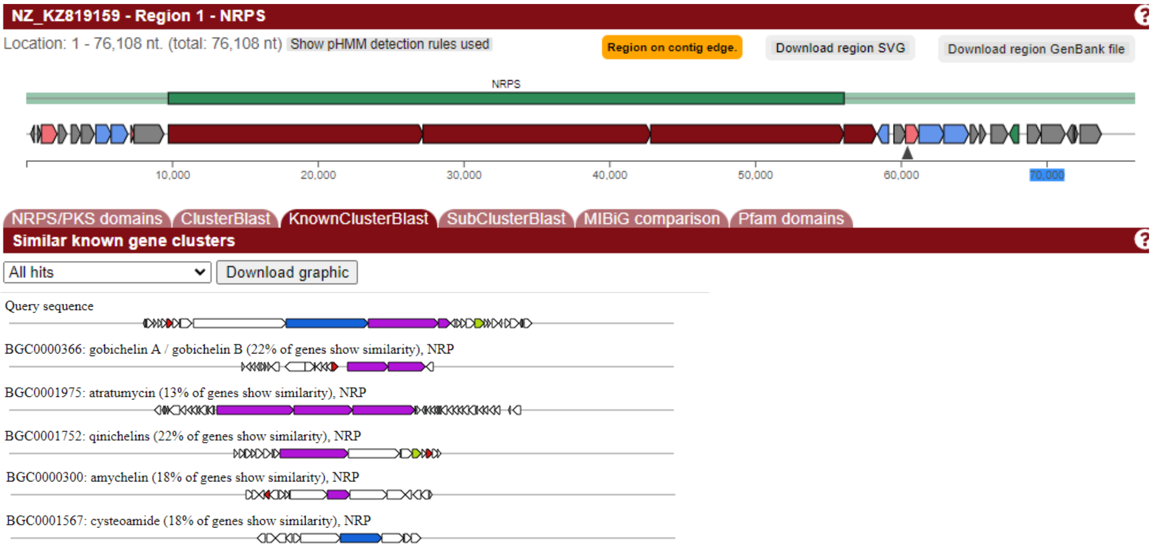

90a.26b

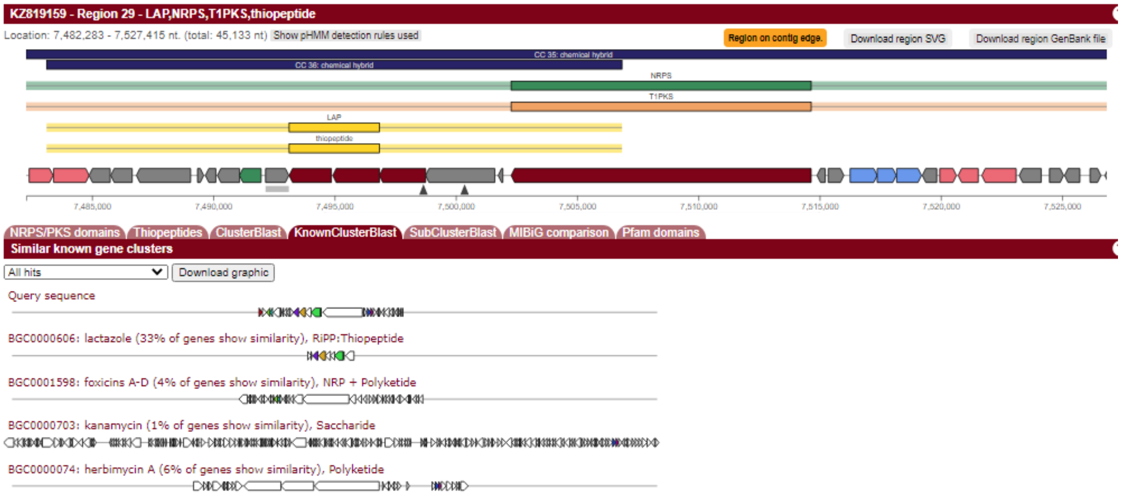

90a.28

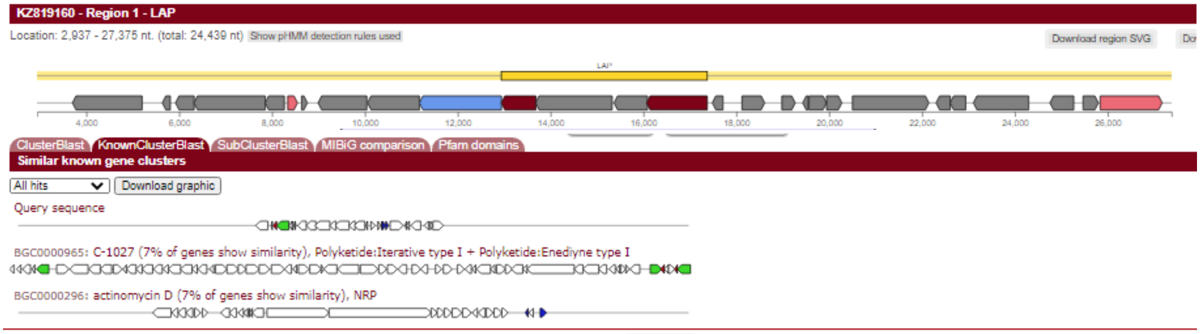

90a.29

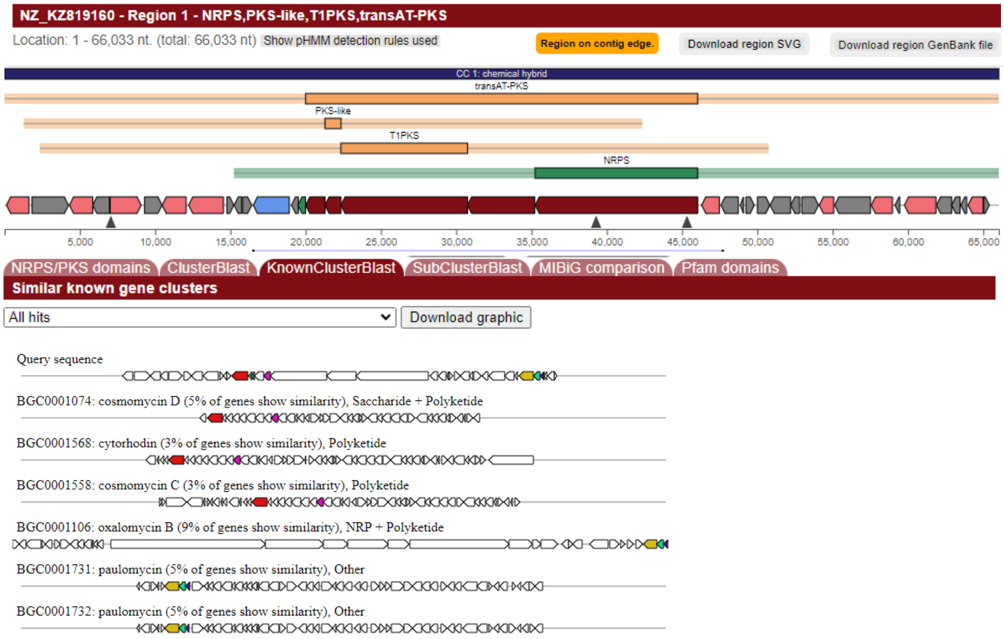

90a.32a

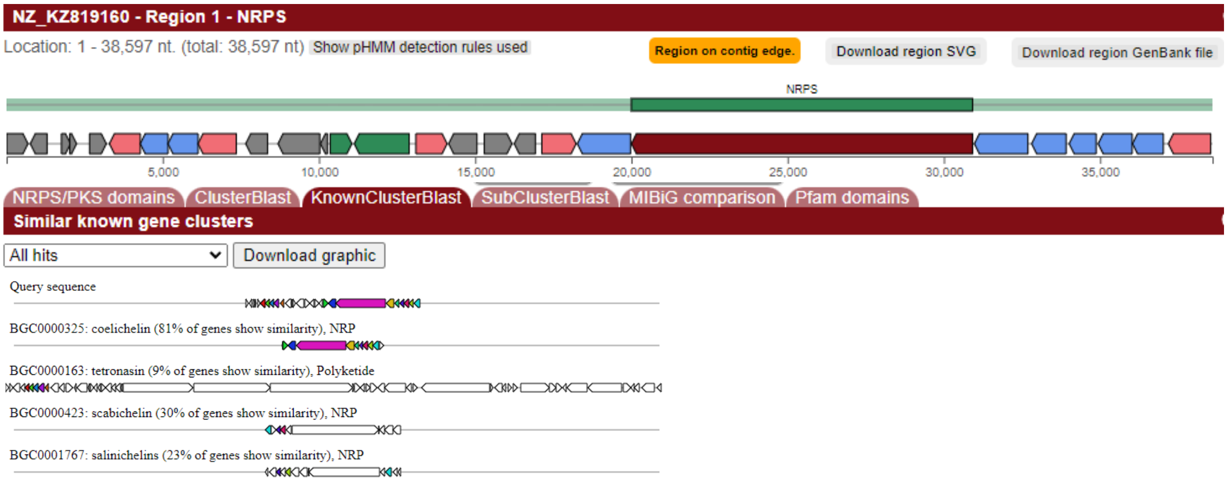

90a.33a

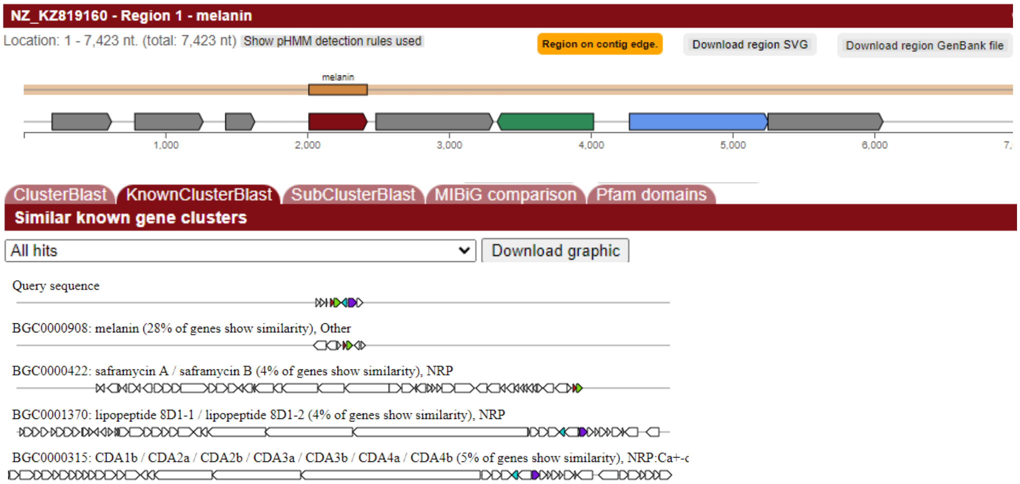

90a.33b

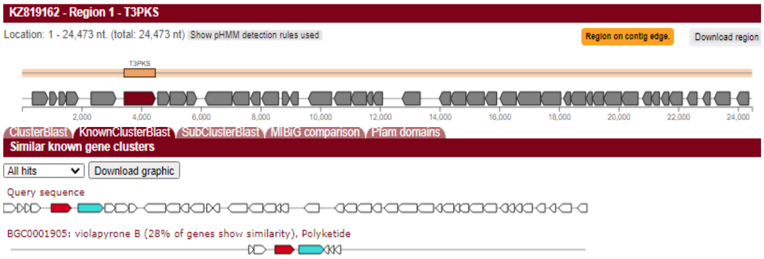

90a.34

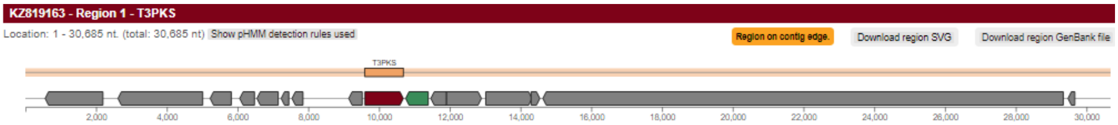

90a.35

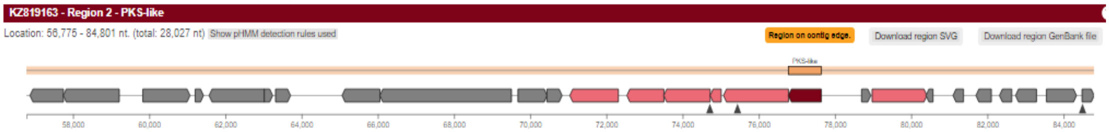

90a.36

Figure S6. Genetic organization of BGCs classified as singletons in CS090a. The KnownClusterBlast results (if any) are included for comparative purposes. Data obtained from antiSMASH v.5.2.

Singletons – CS113

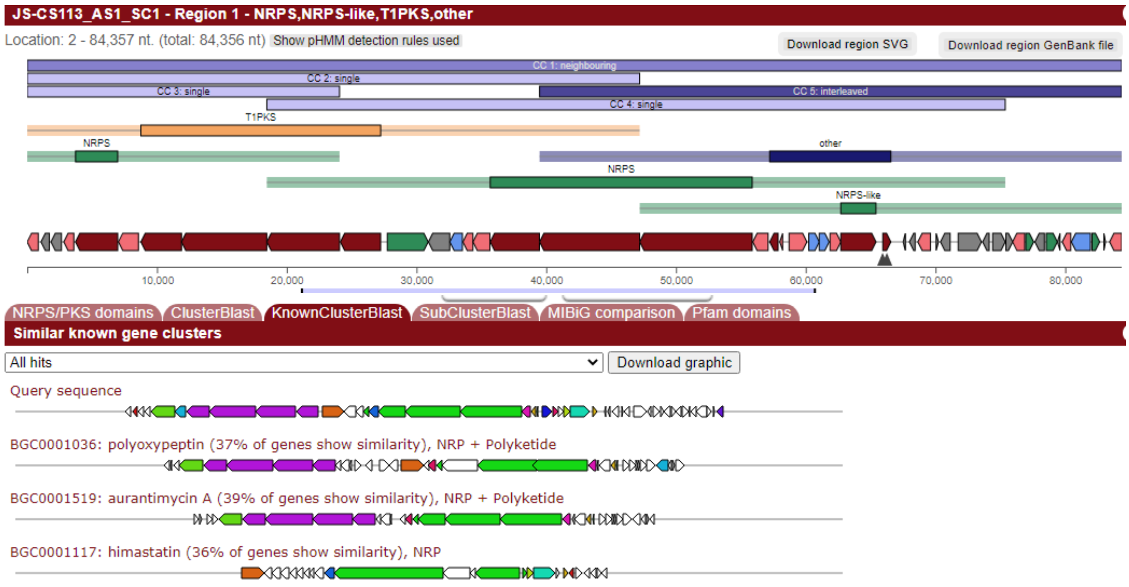

113.1

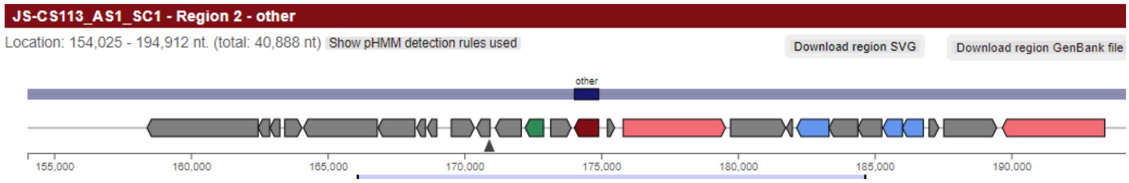

113.2

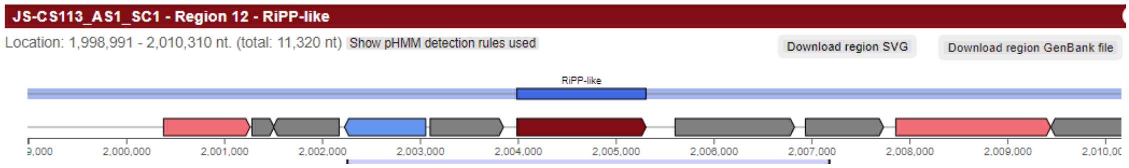

113.12

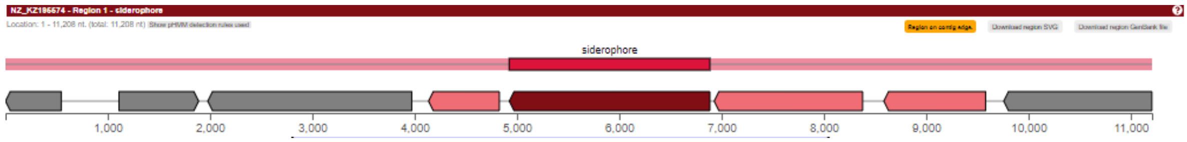

113.14a

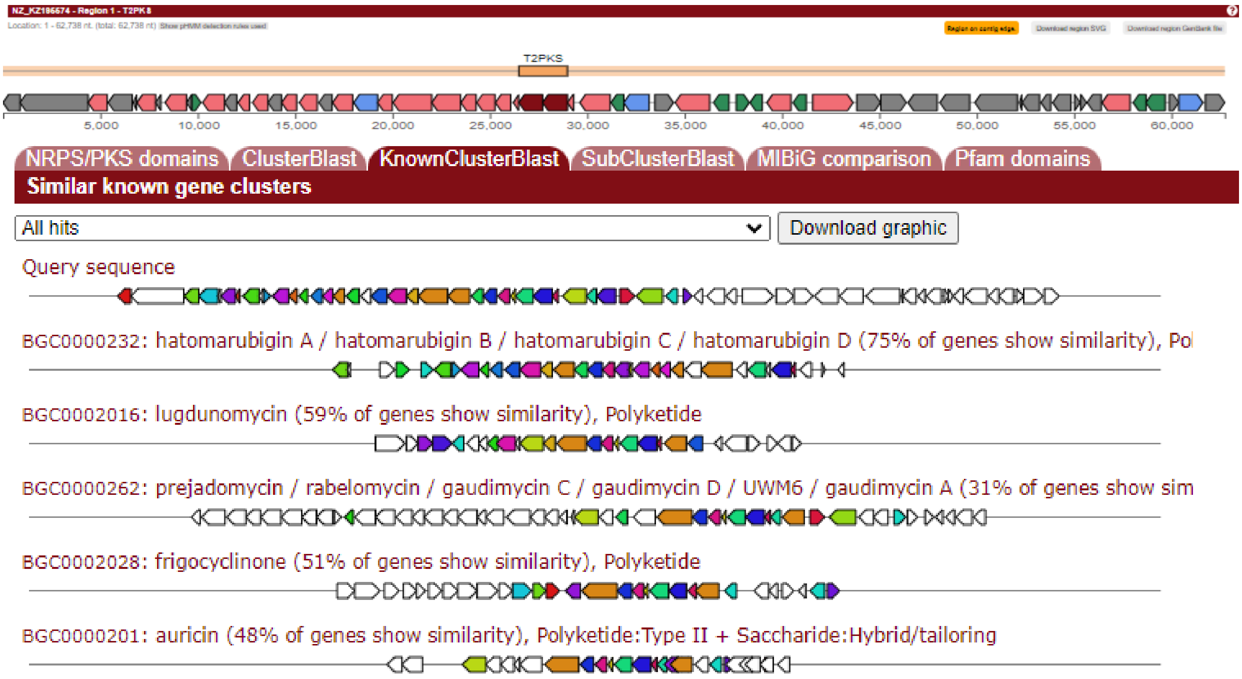

113.14b

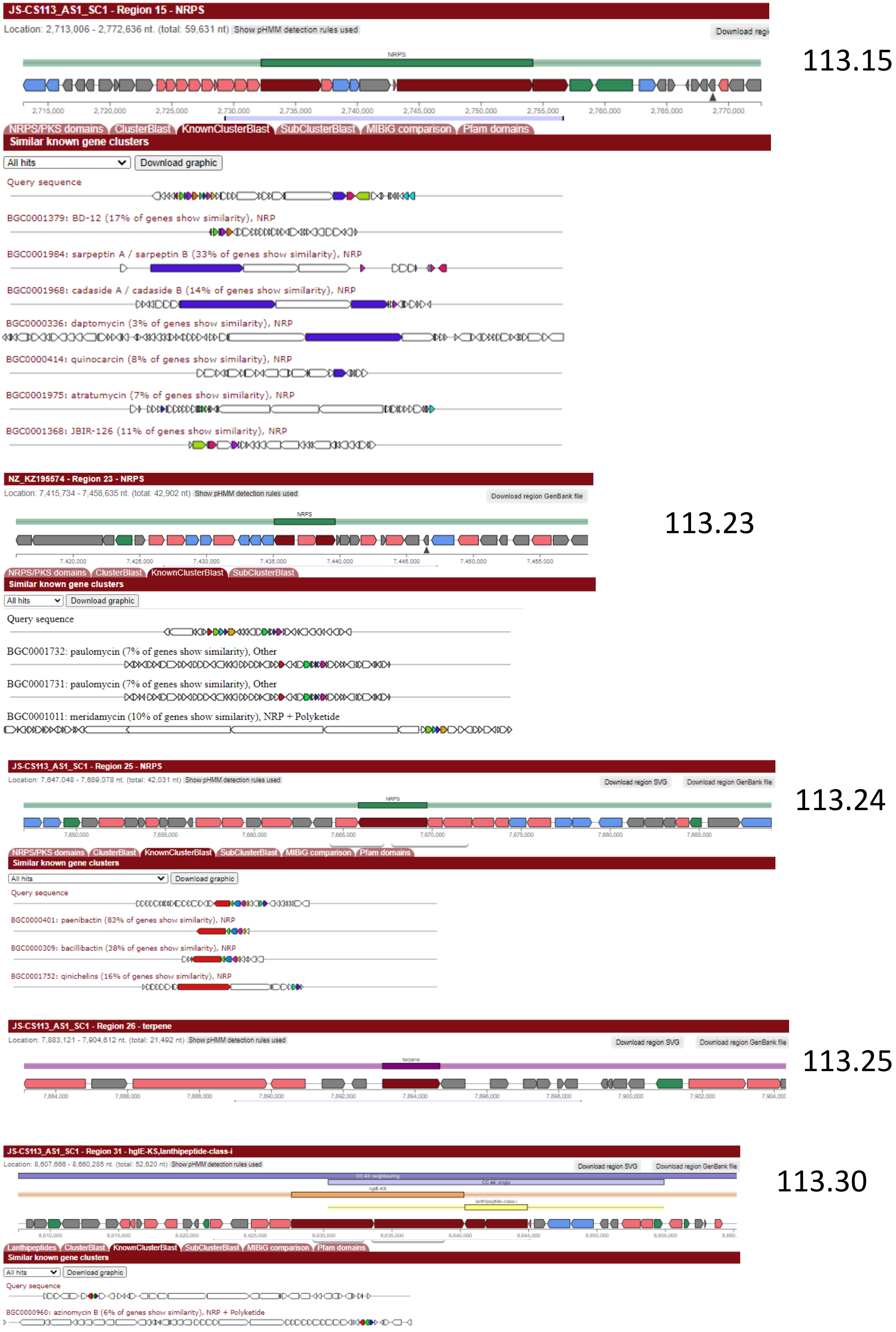

Figure S7. Genetic organization of BGCs classified as singletons in CS113. The KnownClusterBlast results (if any) are included for comparative purposes. Data obtained from antiSMASH v.5.2.

Singletons – CS131

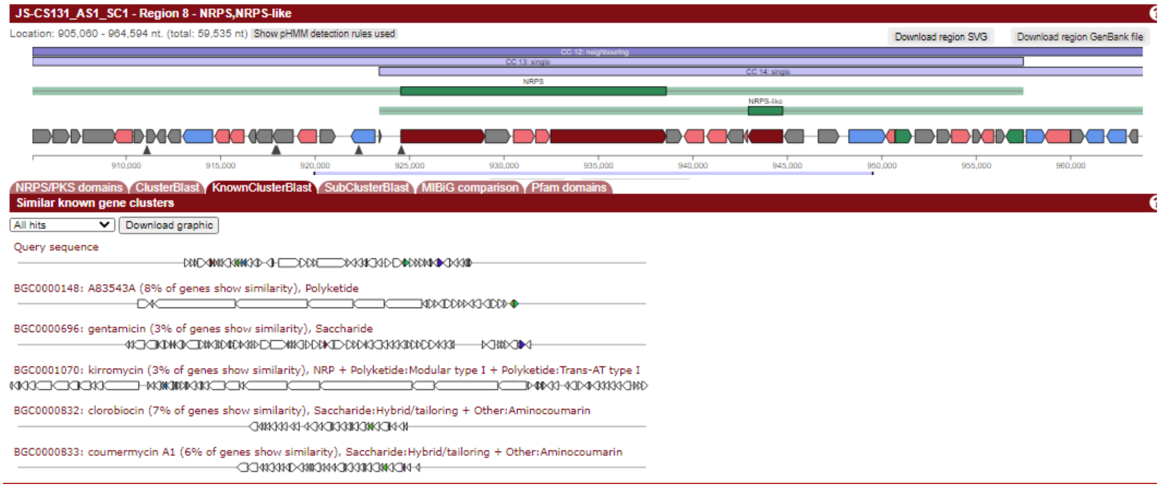

131.8

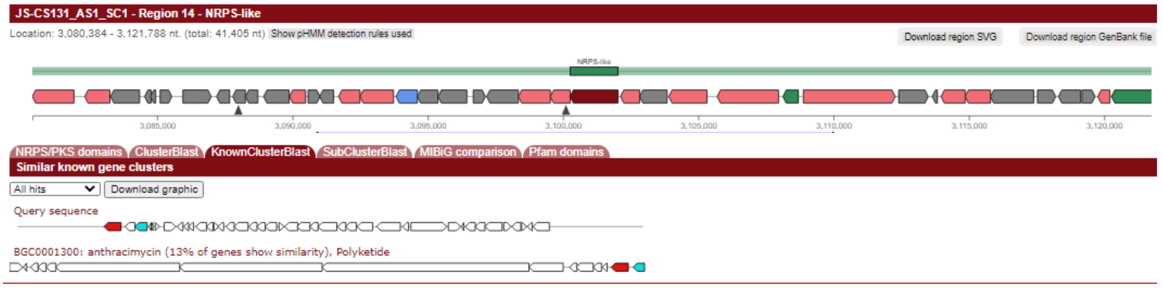

131.14

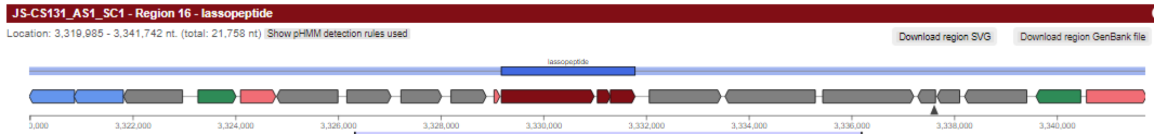

131.16

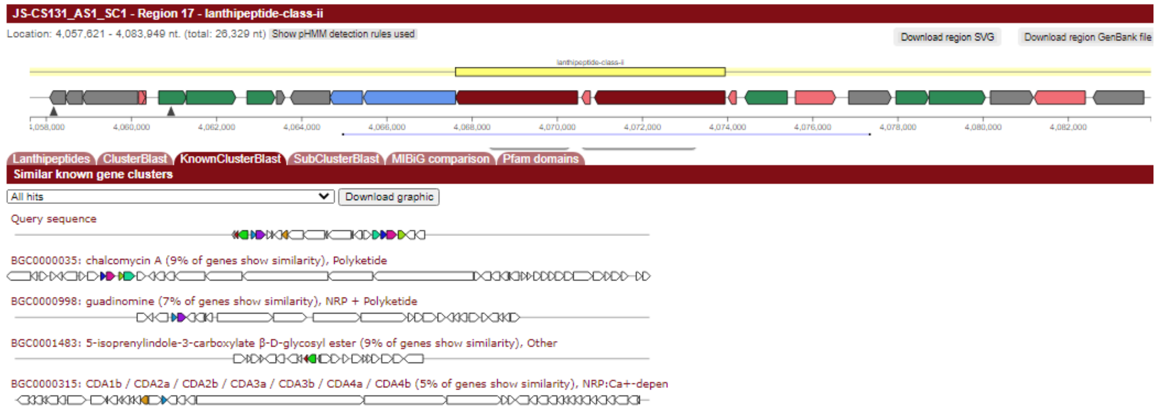

131.17

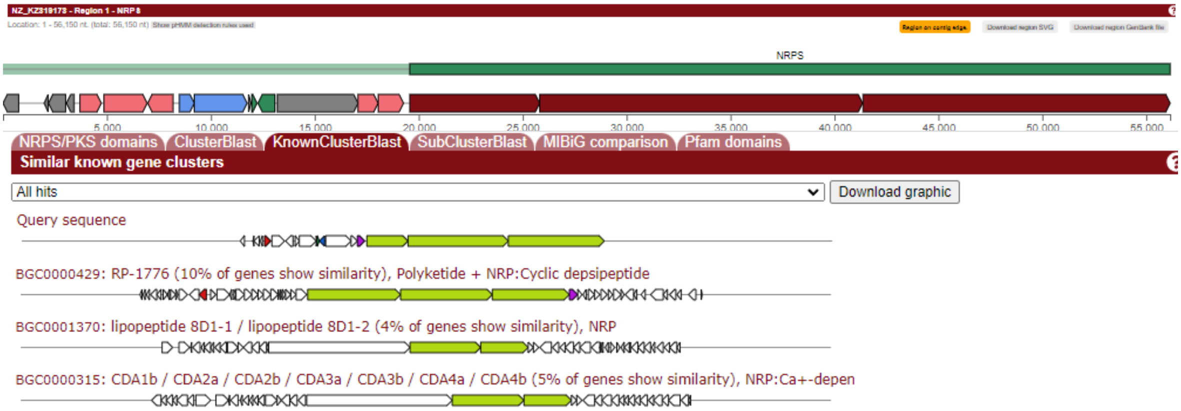

131.18a

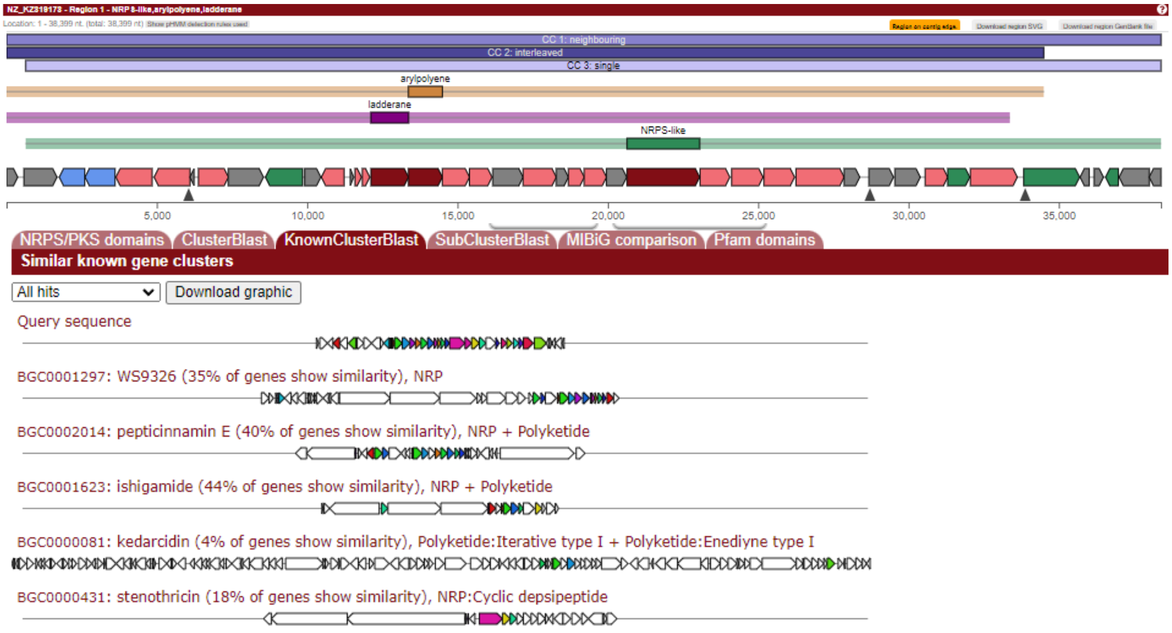

131.18b

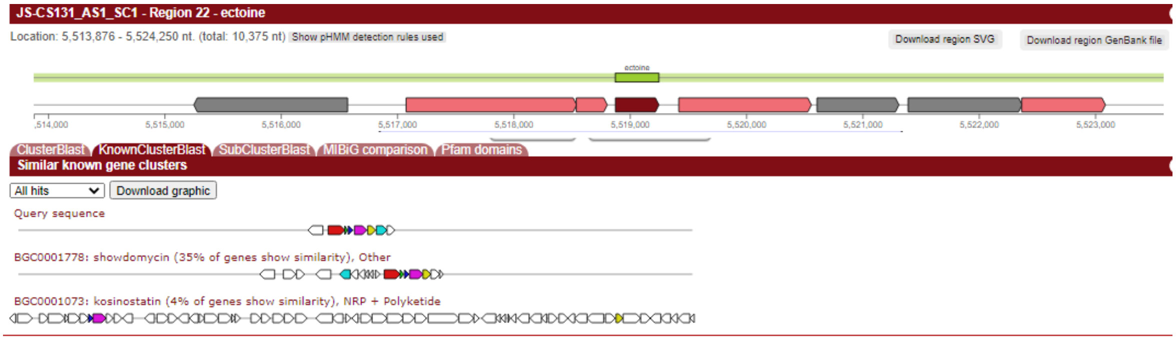

131.22

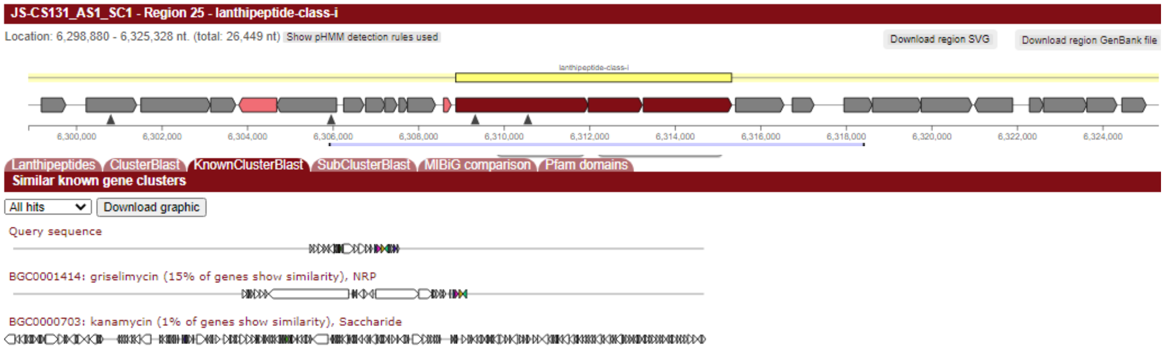

131.25

Figure S8. Genetic organization of BGCs classified as singletons in CS131. The KnownClusterBlast results (if any) are included for comparative purposes. Data obtained from antiSMASH v.5.2.

Singletons – CS147

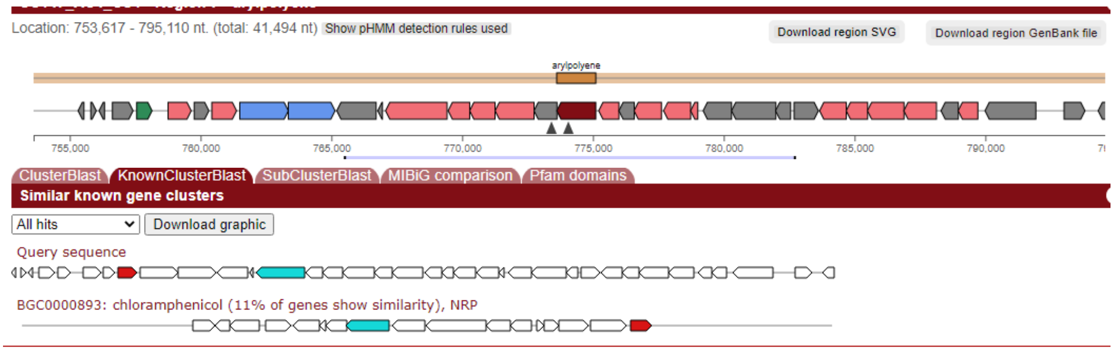

147.6

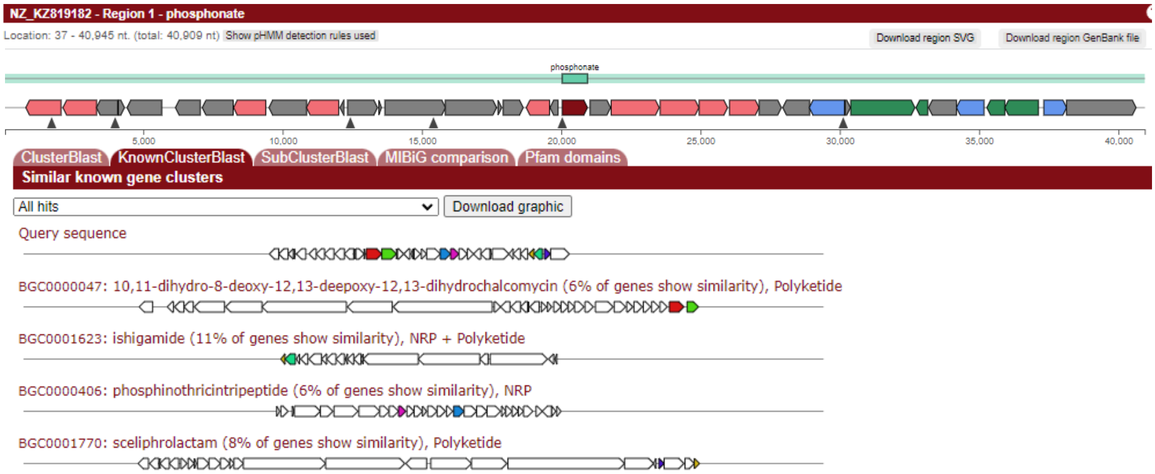

147.13a

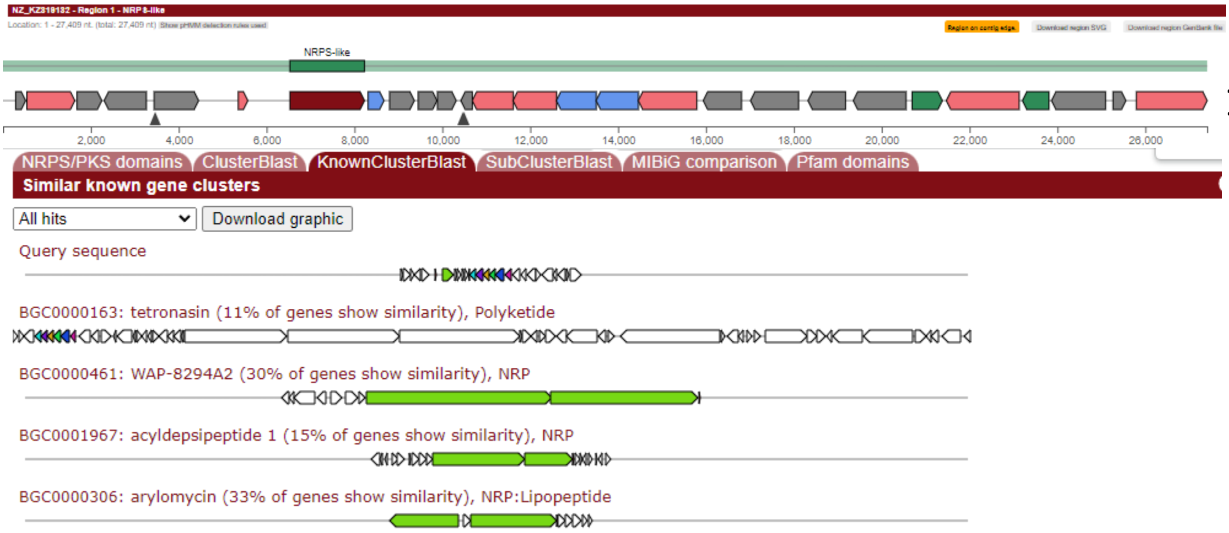

147.29b

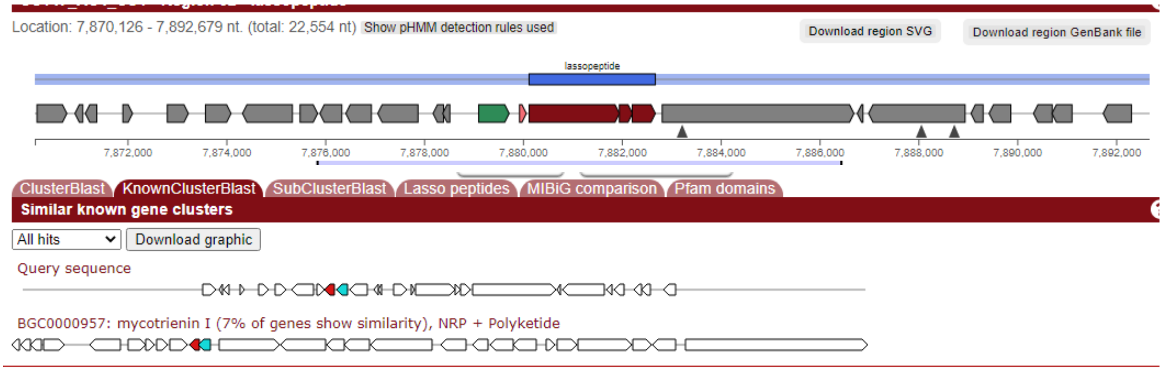

147.31

Figure S9. Genetic organization of BGCs classified as singletons in CS147. The KnownClusterBlast results (if any) are included for comparative purposes. Data obtained from antiSMASH v.5.2.

Singletons – CS149

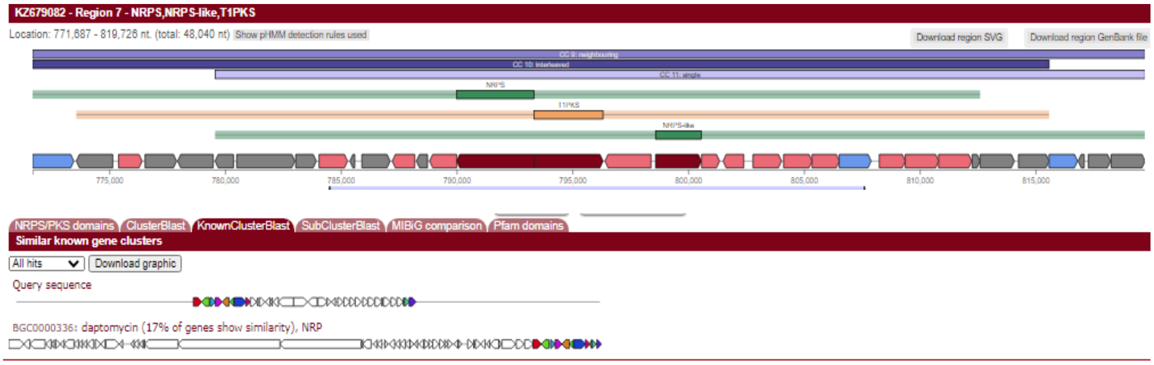

149.7

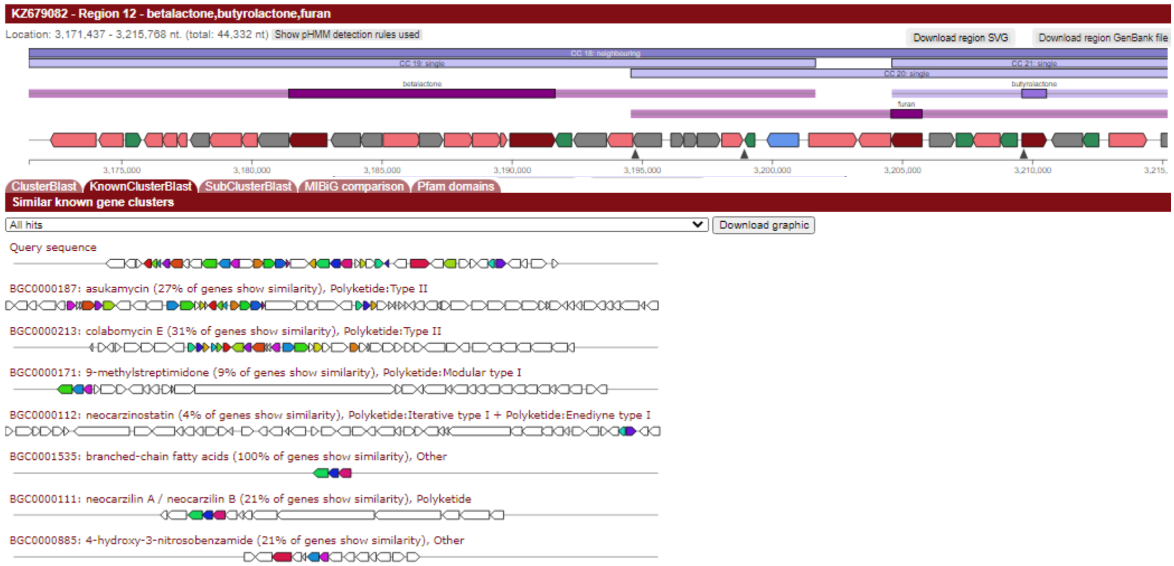

149.12

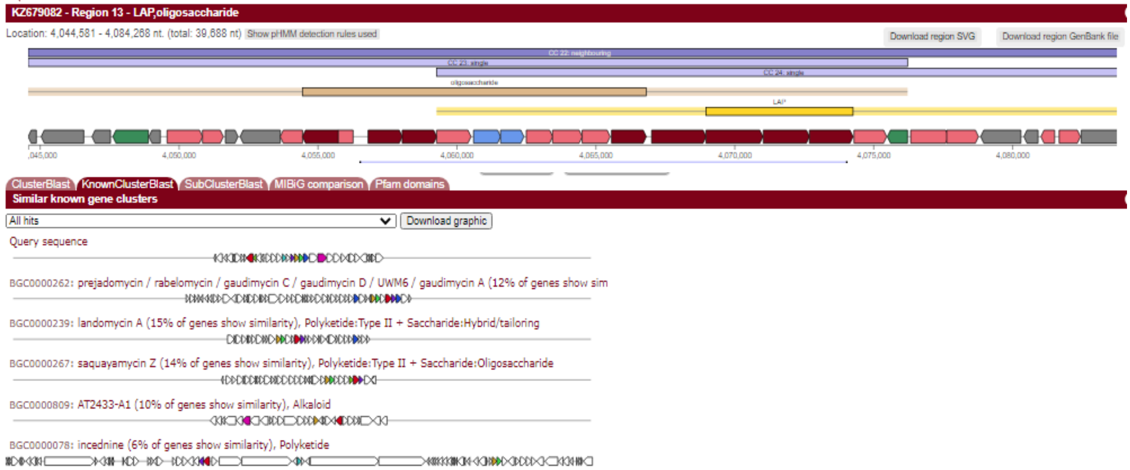

149.13

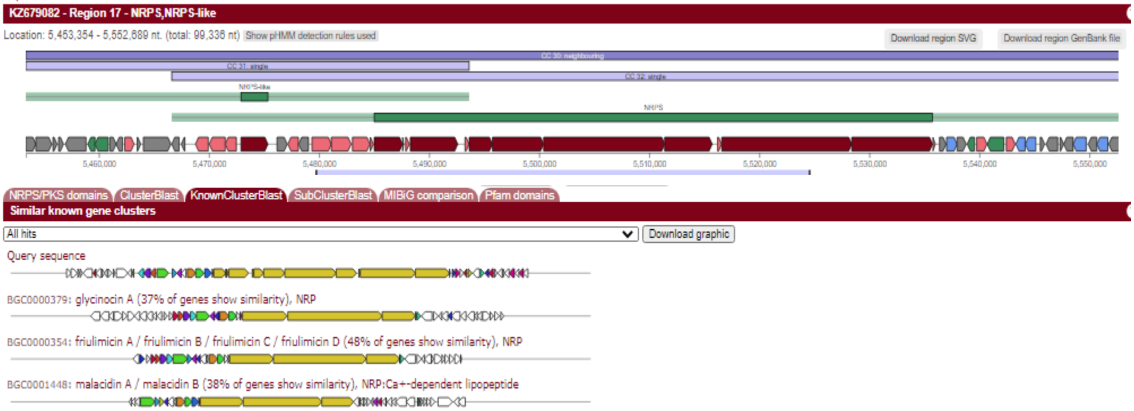

149.17

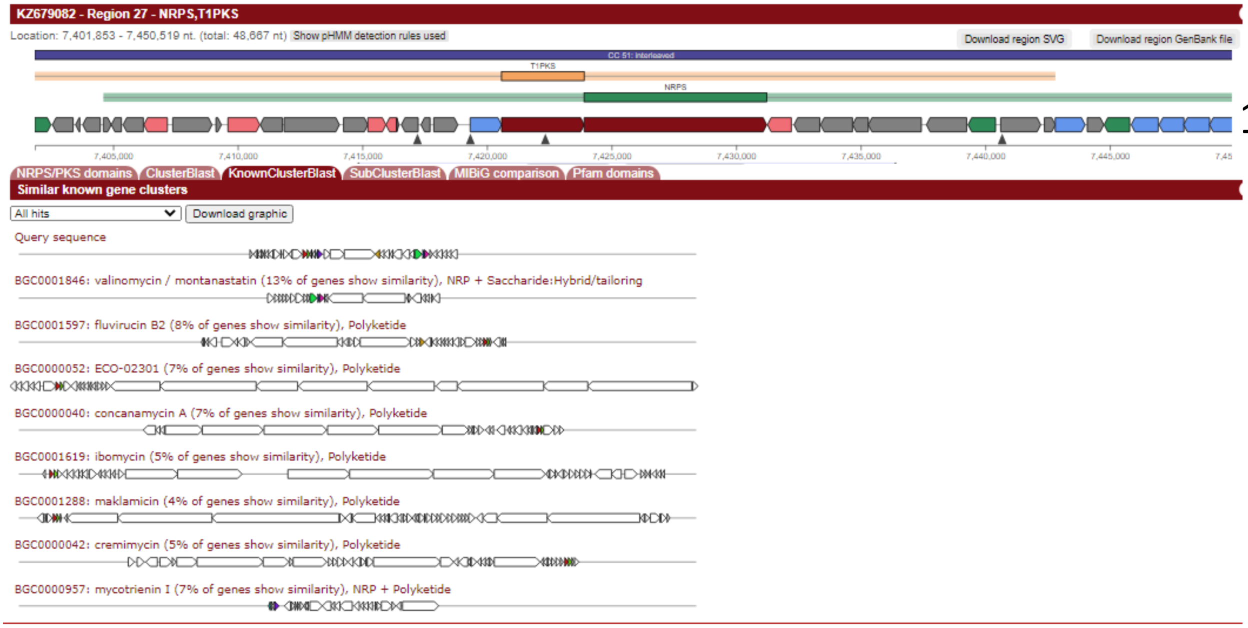

149.27

Figure S10. Genetic organization of BGCs classified as singletons in CS149. The KnownClusterBlast results (if any) are included for comparative purposes. Data obtained from antiSMASH v.5.2.

Singletons – CS159

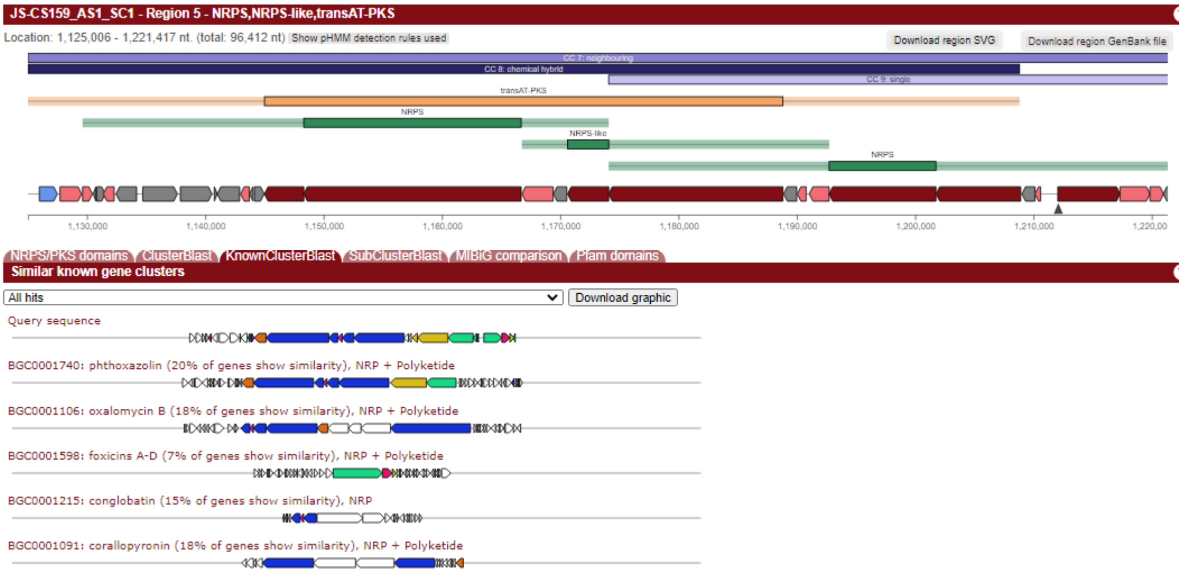

159.5

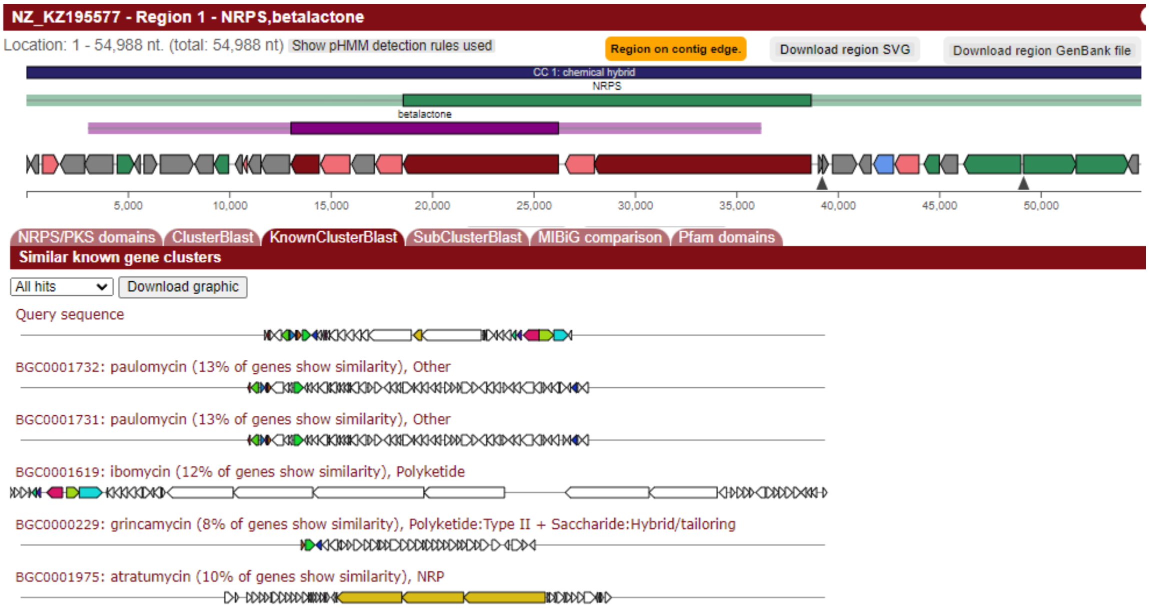

159.7a

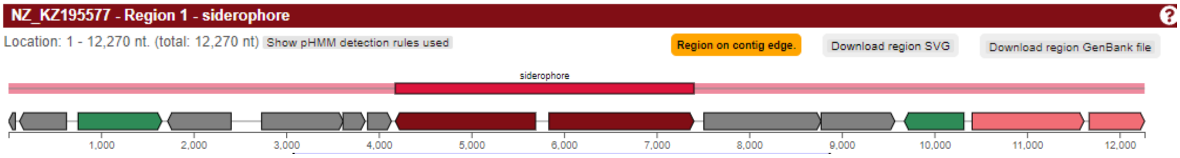

159.7b

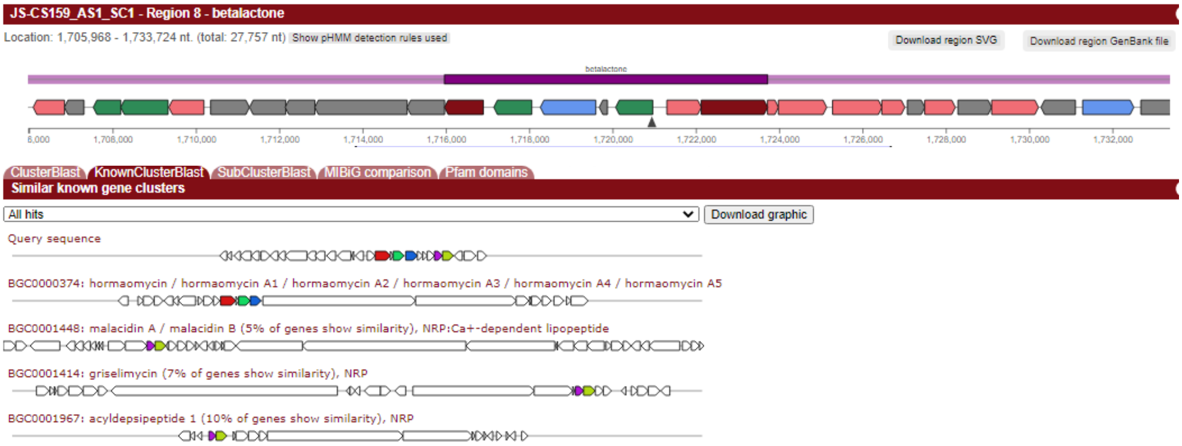

159.8

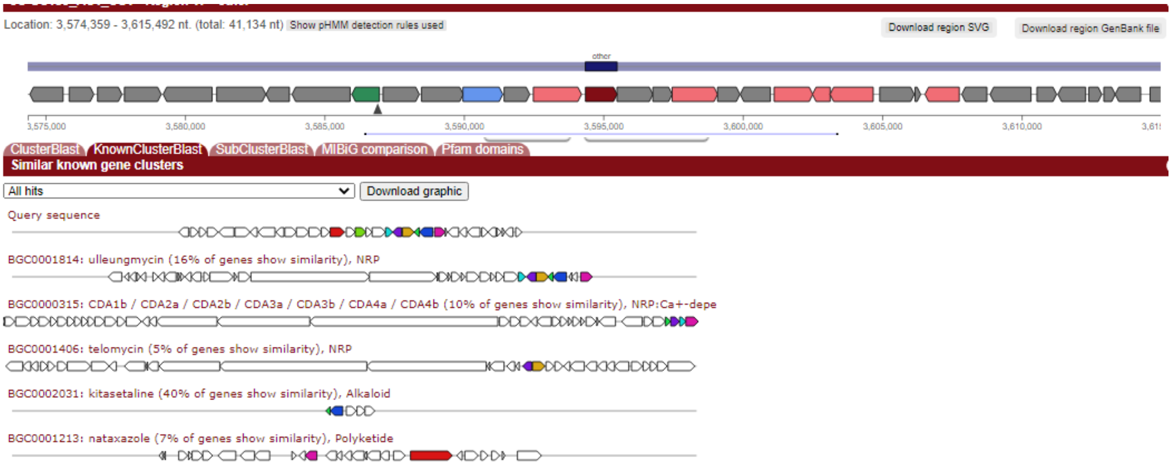

159.15

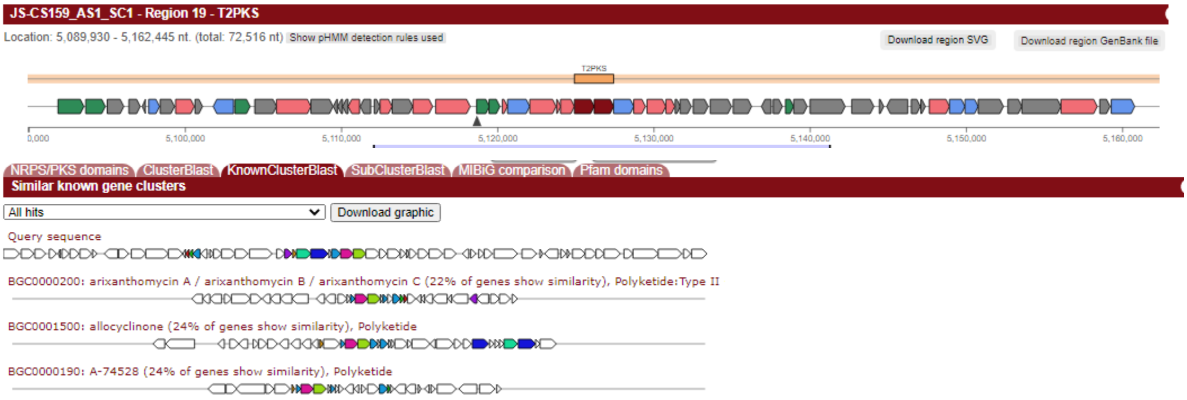

159.17

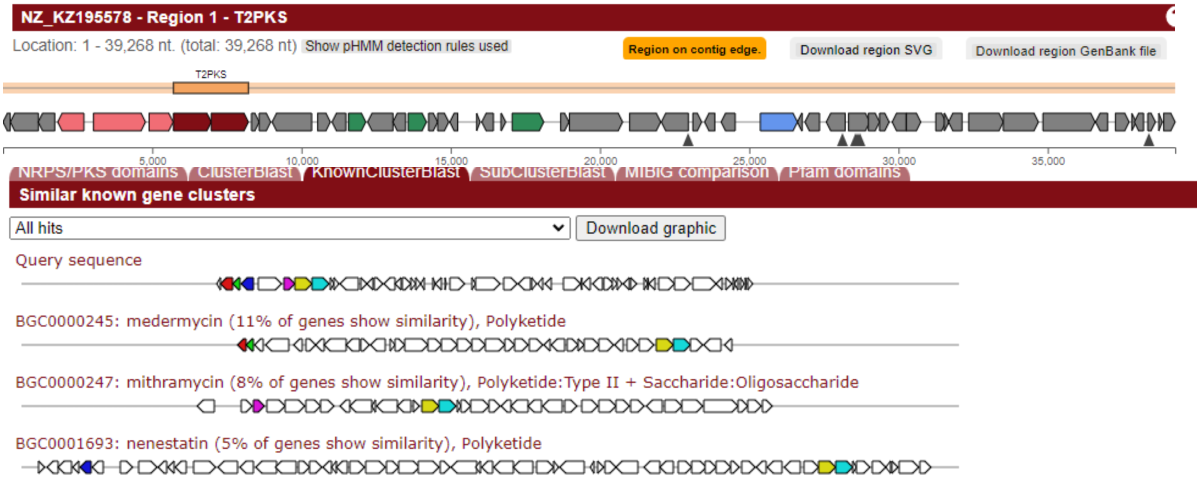

159.26

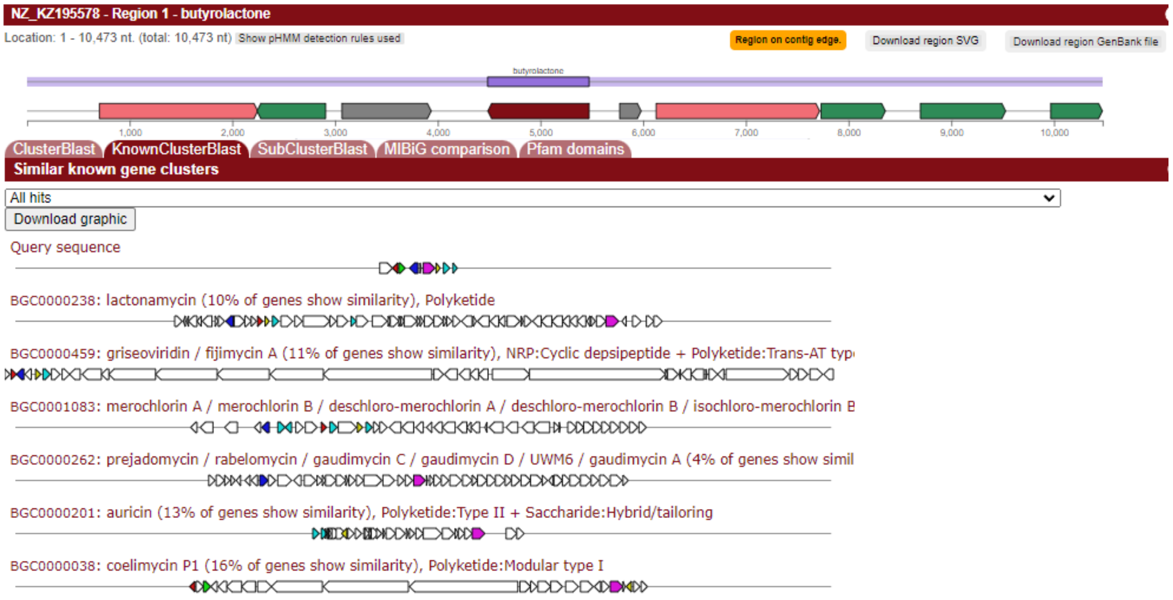

159.27

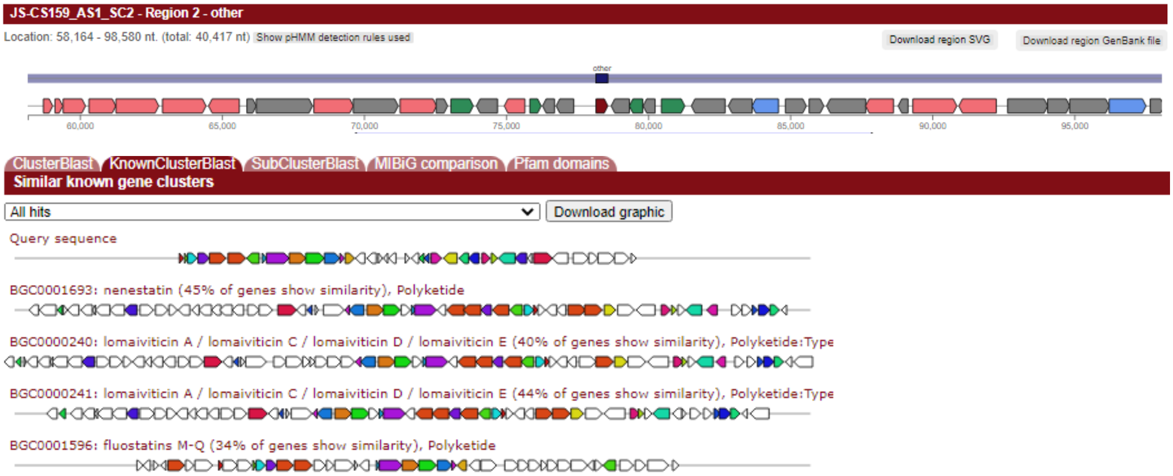

159.28

Figure S11. Genetic organization of BGCs classified as singletons in CS159. The KnownClusterBlast results (if any) are included for comparative purposes. Data obtained from antiSMASH v.5.2.

Singletons – CS207

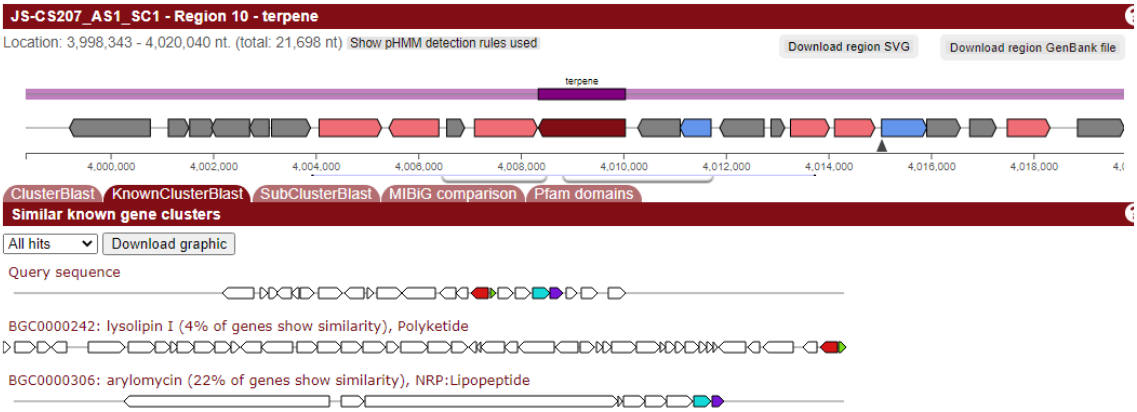

207.10

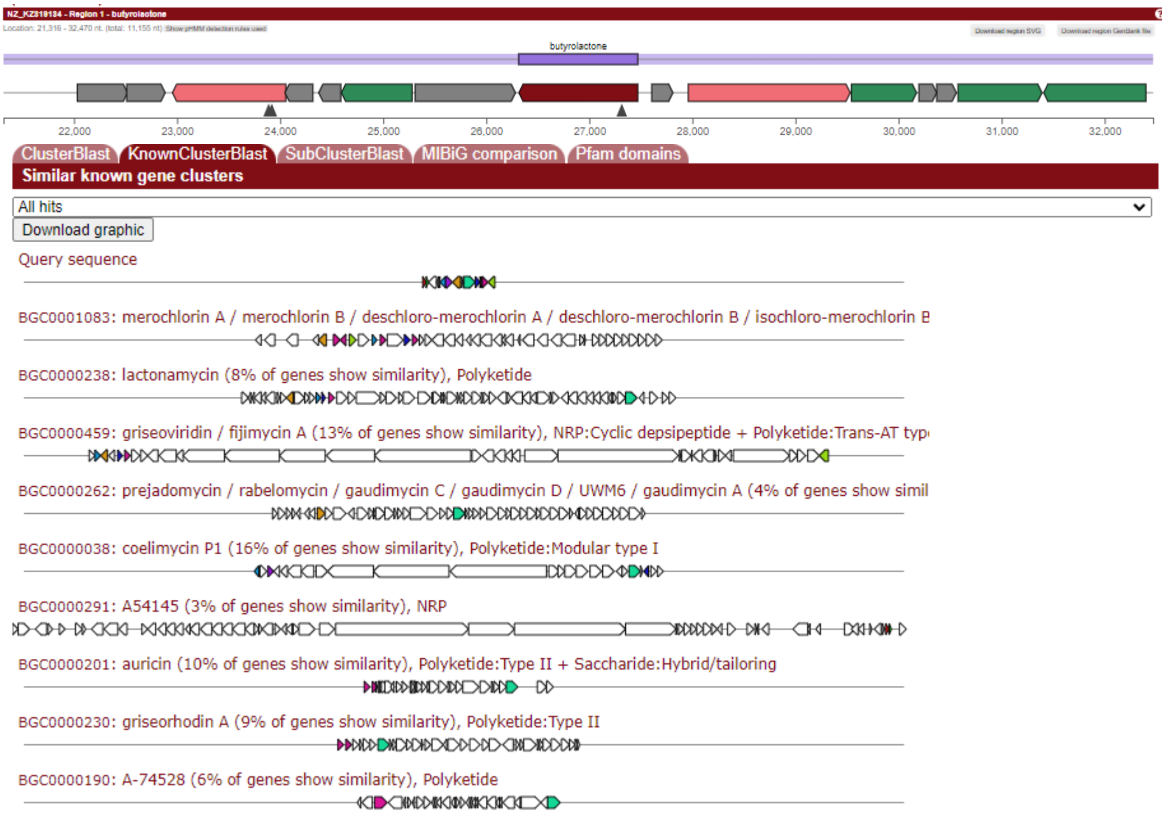

207.12a

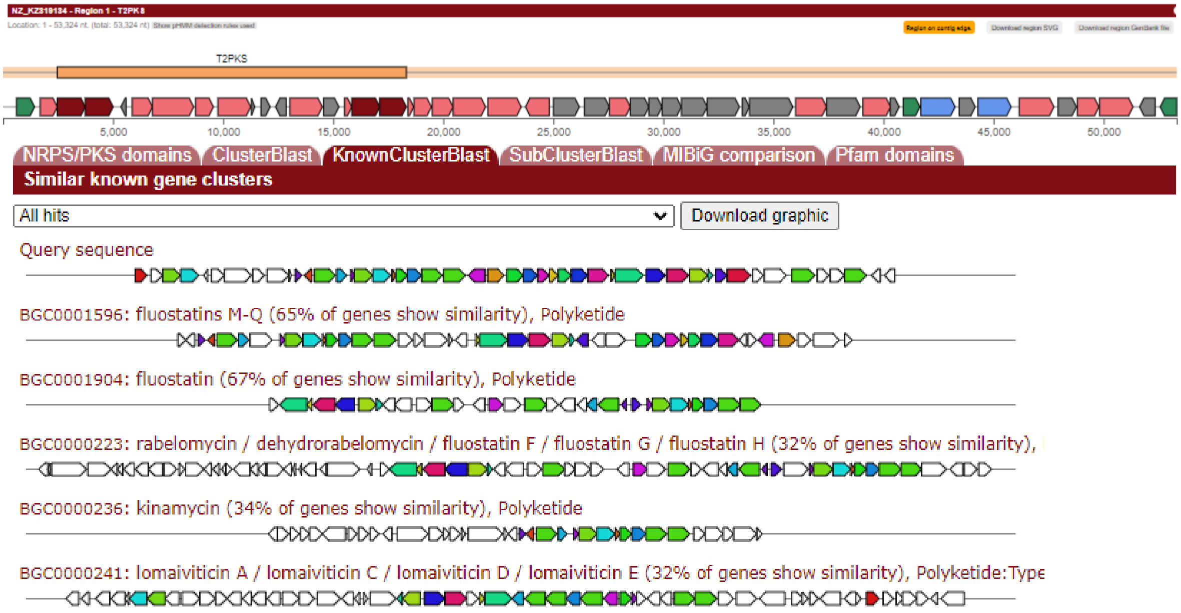

207.12b

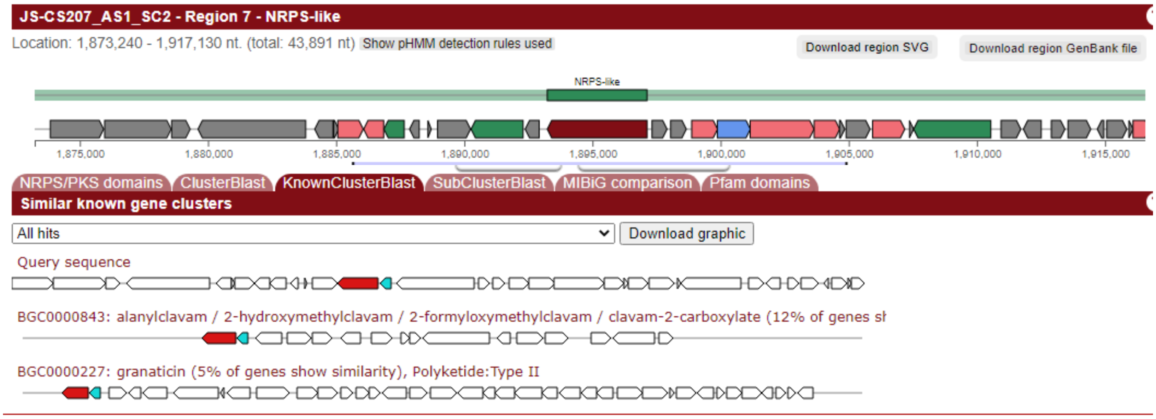

207.19

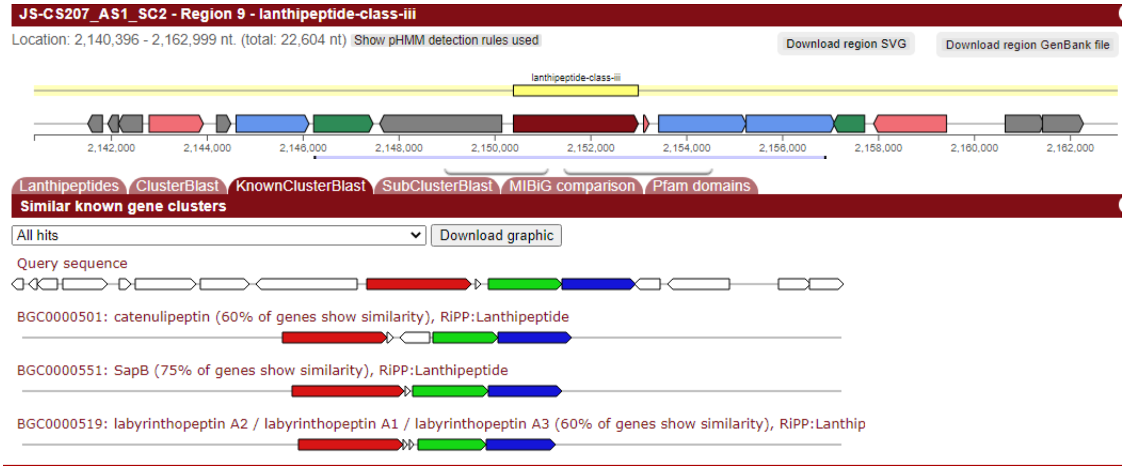

207.20

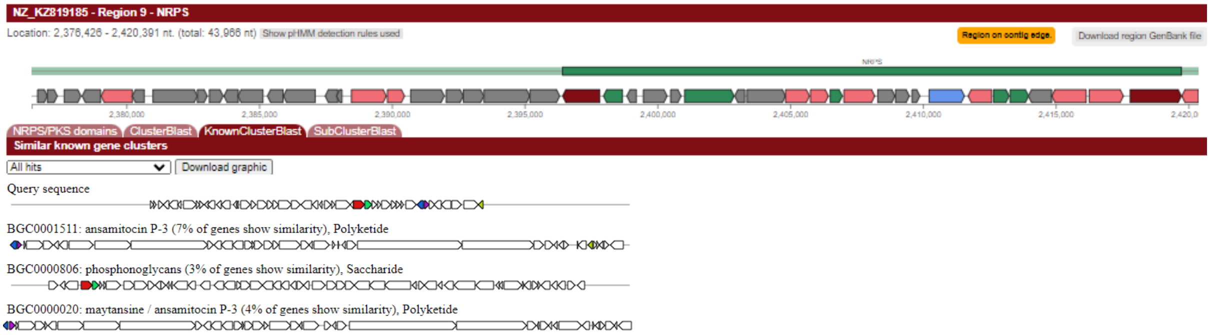

207.21

Figure S12. Genetic organization of BGCs classified as singletons in CS207. The KnownClusterBlast results (if any) are included for comparative purposes. Data obtained from antiSMASH v.5.2.

Singletons – CS227

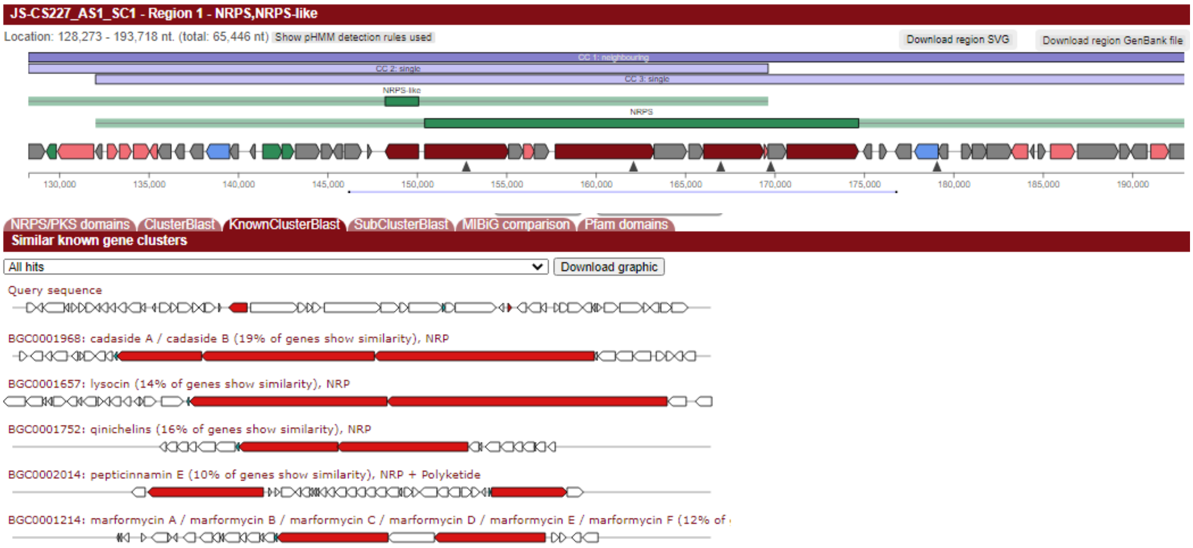

227.1

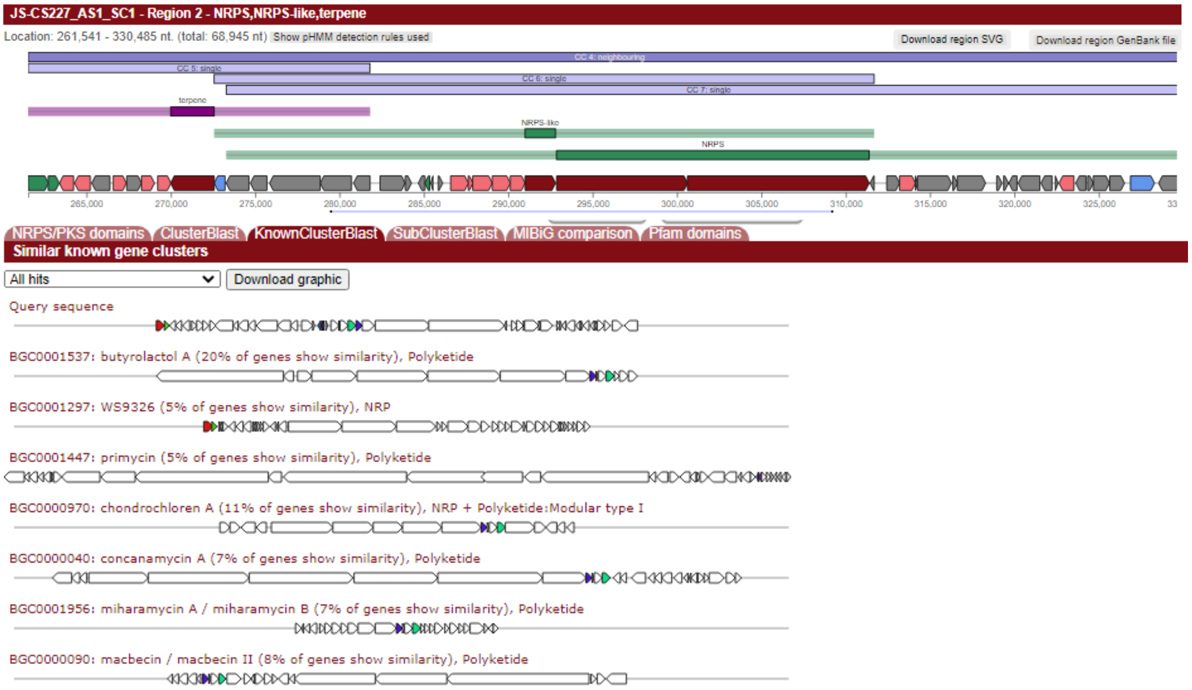

227.2

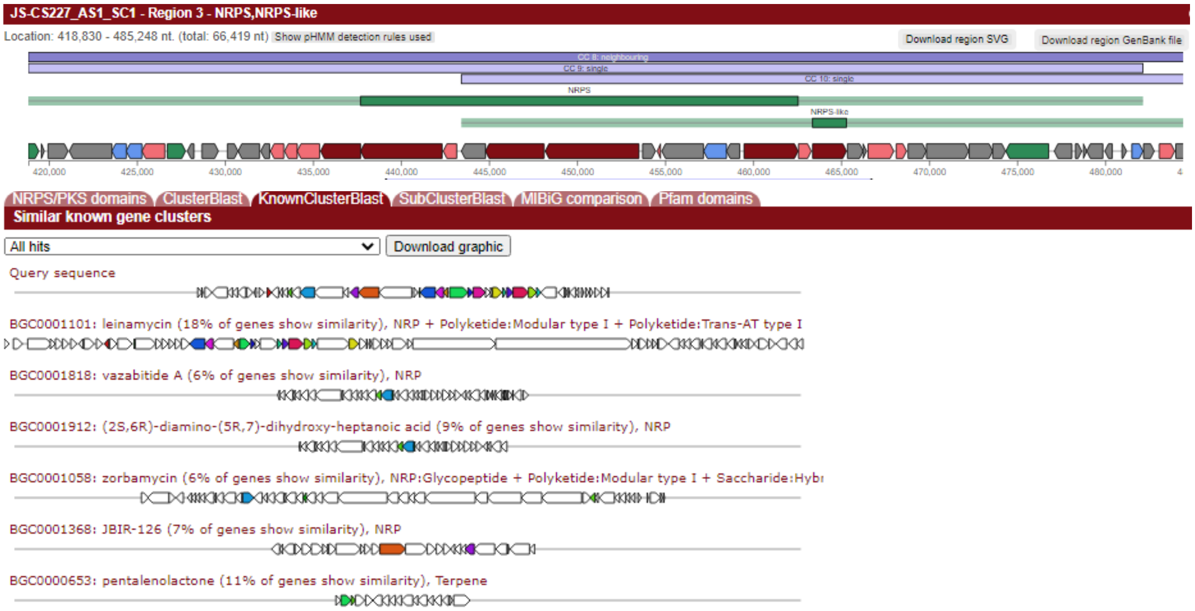

227.3

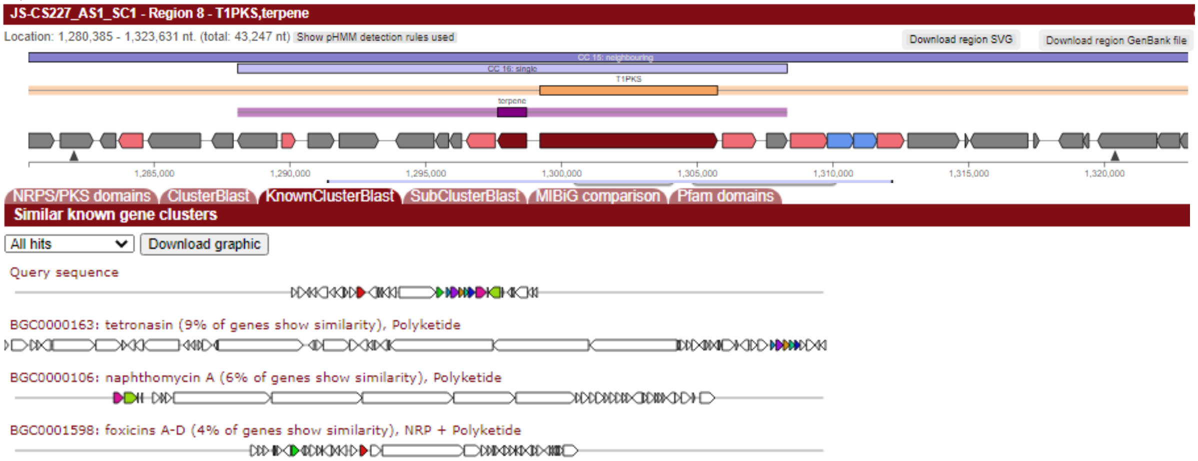

227.8

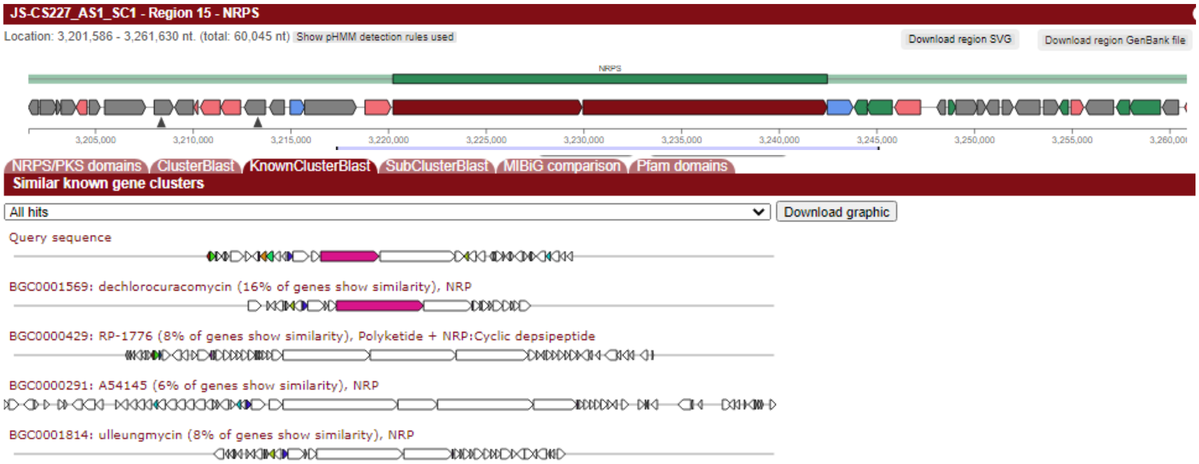

227.15

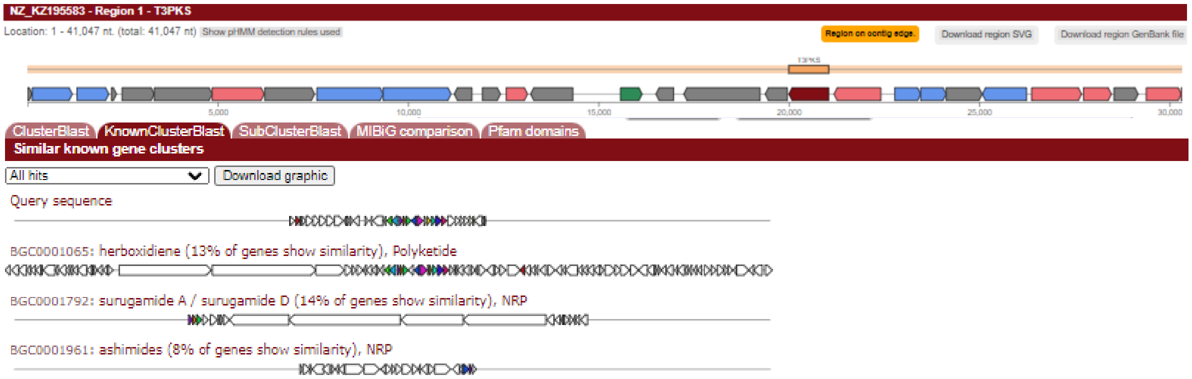

227.20a

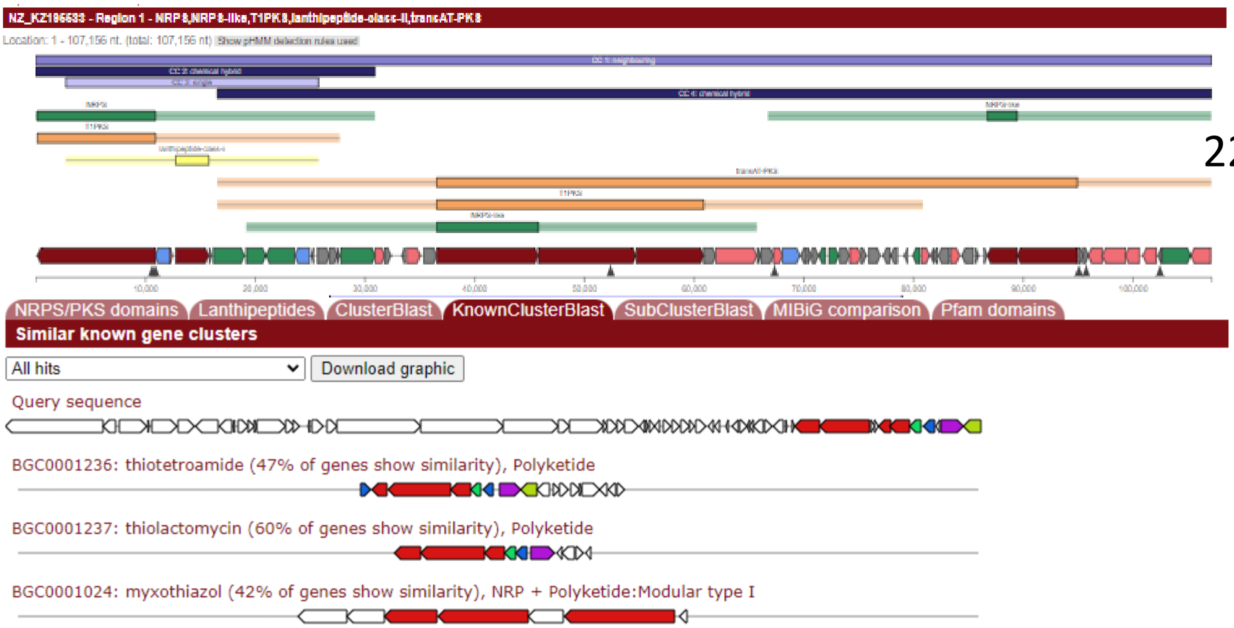

227.20d

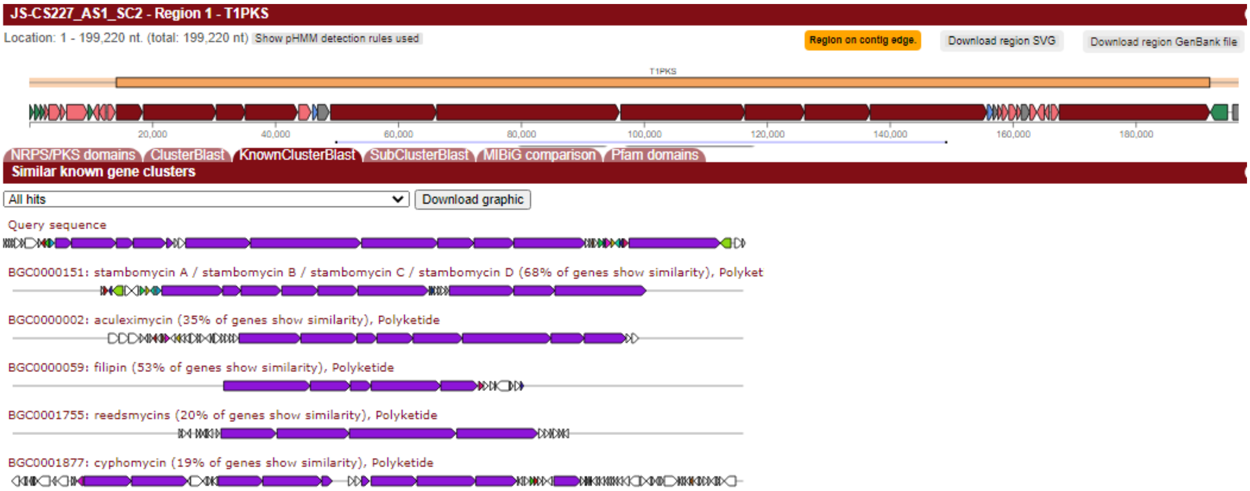

227.21

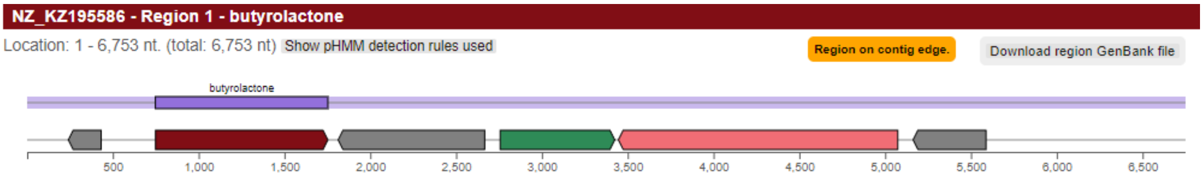

227.22

Figure S13. Genetic organization of BGCs classified as singletons in CS227. The KnownClusterBlast results (if any) are included for comparative purposes. Data obtained from antiSMASH v.5.2.
