# Supplementary material for: A Multidisciplinary Approach to Unraveling the Natural Product Biosynthetic Potential of a Streptomyces Strain Collection Isolated from Leaf-Cutting Ants
Source: Microorganisms. 2021 Oct 26;9(11):2225. doi: 10.3390/microorganisms9112225 (PMC8621525; doi:10.3390/microorganisms9112225)
Supplement: Supplementary file 1 [file microorganisms-09-02225-s001.zip › Table S1. Housekeeping genes used in MLSA.pdf]

Table S1. Housekeeping genes used in MLSA analysis.

Accession	Name	Cellular process	Description
TIGR01798	cit_synth_I	Energy metabolism	citrate (Si)-synthase
TIGR00019	prfA	Protein synthesis	peptide chain release factor 1
TIGR03953	rplD_bact	Protein synthesis	50S ribosomal protein uL4
TIGR00119	acolac_sm	Amino acid biosynthesis	acetolactate synthase, small subunit
TIGR01855	IMP_synth_hisH	Amino acid biosynthesis	imidazole glycerol phosphate synthase, glutamine amidotransferase subunit
TIGR01959	nuoF_fam	Energy metabolism	NADH oxidoreductase (quinone), F subunit
TIGR00114	lumazine-synth	Biosynthesis of cofactors, prosthetic groups, and carriers	6,7-dimethyl-8-ribityllumazine synthase
TIGR00138	rsmG_gidB	Protein synthesis	16S rRNA (guanine(527)-N(7))-methyltransferase RsmG
TIGR00431	TruB	Protein synthesis	tRNA pseudouridine(55) synthase
TIGR00244	TIGR00244	Regulatory functions	transcriptional regulator NrdR
TIGR00150	T6A_YjeE	Protein synthesis	tRNA threonylcarbamoyl adenosine modification protein YjeE
TIGR00174	miaA	Protein synthesis	tRNA dimethylallyltransferase
TIGR00521	coaBC_dfp	Biosynthesis of cofactors, prosthetic groups, and carriers	phosphopantothenoylcysteine decarboxylase / phosphopantothenate--cysteine ligase
TIGR00222	panB	Biosynthesis of cofactors, prosthetic groups, and carriers	3-methyl-2-oxobutanoate hydroxymethyltransferase
TIGR00313	cobQ	Biosynthesis of cofactors, prosthetic groups, and carriers	cobyric acid synthase CobQ
TIGR00038	efp	Protein synthesis	translation elongation factor P
TIGR00763	lon	Protein fate	endopeptidase La
TIGR03725	T6A_YeaZ	Protein synthesis	tRNA threonylcarbamoyl adenosine modification protein YeaZ
TIGR00090	rsfS_iojap_ybeB	Protein synthesis	ribosome silencing factor
TIGR00518	alaDH	Energy metabolism	alanine dehydrogenase
TIGR00445	mraY	Cell envelope	phospho-N-acetylmuramoyl-pentapeptide-transferase
TIGR00331	hrcA	Regulatory functions	heat-inducible transcription repressor HrcA
TIGR00096	TIGR00096	Protein synthesis	16S rRNA (cytidine(1402)-2'-O)-methyltransferase
TIGR00482	TIGR00482	Biosynthesis of cofactors,	nicotinate (nicotinamide) nucleotide adenylyltransferase

		prosthetic groups, and carriers	
TIGR00355	purH	Purines, pyrimidines, nucleosides, and nucleotides	phosphoribosylaminoimidazolecarboxamide formyltransferase/IMP cyclohydrolase
TIGR01973	NuoG	Energy metabolism	NADH dehydrogenase (quinone), G subunit
TIGR01736	FGAM_synth_II	Purines, pyrimidines, nucleosides, and nucleotides	phosphoribosylformylglycinamidine synthase II
TIGR00338	serB	Amino acid biosynthesis	phosphoserine phosphatase SerB
TIGR00420	trmU	Protein synthesis	tRNA (5-methylaminomethyl-2-thiouridylate)-methyltransferase
TIGR01066	rplM_bact	Protein synthesis	ribosomal protein uL13
TIGR00962	atpA	Energy metabolism	ATP synthase F1, alpha subunit
TIGR01063	gyrA	DNA metabolism	DNA gyrase, A subunit
TIGR00720	sda_mono	Energy metabolism	L-serine ammonia-lyase
TIGR01127	ilvA_1Cterm	Amino acid biosynthesis	threonine ammonia-lyase
TIGR01009	rpsC_bact	Protein synthesis	ribosomal protein uS3
TIGR01280	xseB	DNA metabolism	exodeoxyribonuclease VII, small subunit
TIGR01021	rpsE_bact	Protein synthesis	ribosomal protein uS5
TIGR01024	rplS_bact	Protein synthesis	ribosomal protein bL19
TIGR00168	infC	Protein synthesis	translation initiation factor IF-3
TIGR03705	poly_P_kin	Central intermediary metabolism	polyphosphate kinase 1
TIGR01966	RNasePH	Transcription	ribonuclease PH
TIGR00382	clpX	Protein fate	ATP-dependent Clp protease, ATP-binding subunit ClpX
TIGR01203	HGPRTase	Purines, pyrimidines, nucleosides, and nucleotides	hypoxanthine phosphoribosyltransferase
TIGR00065	ftsZ	Cellular processes	cell division protein FtsZ
TIGR00048	rRNA_mod_RlmN	Protein synthesis	23S rRNA (adenine(2503)-C(2))-methyltransferase
TIGR00281	TIGR00281	DNA metabolism	segregation and condensation protein B
TIGR02729	Obg_CgtA	Protein synthesis	Obg family GTPase CgtA
TIGR00639	PurN	Purines, pyrimidines, nucleosides, and nucleotides	phosphoribosylglycinamide formyltransferase
TIGR01683	thiS	Biosynthesis of cofactors, prosthetic groups, and carriers	thiamine biosynthesis protein ThiS
TIGR01394	TypA_BipA	Regulatory functions	GTP-binding protein TypA/BipA

TIGR02075	pyrH_bact	Purines, pyrimidines, nucleosides, and nucleotides	UMP kinase
TIGR00416	sms	DNA metabolism	DNA repair protein RadA
TIGR00228	ruvC	DNA metabolism	crossover junction endodeoxyribonuclease RuvC
TIGR02386	rpoC_TIGR	Transcription	DNA-directed RNA polymerase, beta' subunit
TIGR00755	ksgA	Protein synthesis	ribosomal RNA small subunit methyltransferase A
TIGR00952	S15_bact	Protein synthesis	ribosomal protein uS15
TIGR03631	uS13_bact	Protein synthesis	ribosomal protein uS13
TIGR00071	hisT_truA	Protein synthesis	tRNA pseudouridine(38-40) synthase
TIGR03263	guanyl_kin	Purines, pyrimidines, nucleosides, and nucleotides	guanylate kinase
TIGR00302	TIGR00302	Purines, pyrimidines, nucleosides, and nucleotides	phosphoribosylformylglycinamidine synthase, purS protein
TIGR02257	cobalto_cobN	Biosynthesis of cofactors, prosthetic groups, and carriers	cobaltochelatase, CobN subunit
TIGR00187	ribE	Biosynthesis of cofactors, prosthetic groups, and carriers	riboflavin synthase, alpha subunit
TIGR01455	glmM	Central intermediary metabolism	phosphoglucosamine mutase
TIGR00461	gcvP	Energy metabolism	glycine dehydrogenase
TIGR03594	GTPase_EngA	Protein synthesis	ribosome-associated GTPase EngA
TIGR01464	hemE	Biosynthesis of cofactors, prosthetic groups, and carriers	uroporphyrinogen decarboxylase
TIGR00615	recR	DNA metabolism	recombination protein RecR
TIGR00362	DnaA	DNA metabolism	chromosomal replication initiator protein DnaA
TIGR00086	smpB	Protein synthesis	SsrA-binding protein
TIGR00496	frr	Protein synthesis	ribosome recycling factor
TIGR03654	L6_bact	Protein synthesis	ribosomal protein uL6
TIGR00263	trpB	Amino acid biosynthesis	tryptophan synthase, beta subunit
TIGR01171	rplB_bact	Protein synthesis	ribosomal protein uL2
TIGR01039	atpD	Energy metabolism	ATP synthase F1, beta subunit
TIGR01134	purF	Purines, pyrimidines, nucleosides, and nucleotides	amidophosphoribosyltransferase

TIGR00184	purA	Purines, pyrimidines, nucleosides, and nucleotides	adenylosuccinate synthase
TIGR01737	FGAM_synth_I	Purines, pyrimidines, nucleosides, and nucleotides	phosphoribosylformylglycinamidine synthase I
TIGR00670	asp_carb_tr	Purines, pyrimidines, nucleosides, and nucleotides	aspartate carbamoyltransferase
TIGR01032	rplT_bact	Protein synthesis	ribosomal protein bL20
TIGR01051	topA_bact	DNA metabolism	DNA topoisomerase I
TIGR01011	rpsB_bact	Protein synthesis	ribosomal protein uS2
TIGR01036	pyrD_sub2	Purines, pyrimidines, nucleosides, and nucleotides	dihydroorotate dehydrogenase (fumarate)
