# Supplementary material for: A Multidisciplinary Approach to Unraveling the Natural Product Biosynthetic Potential of a Streptomyces Strain Collection Isolated from Leaf-Cutting Ants
Source: Microorganisms. 2021 Oct 26;9(11):2225. doi: 10.3390/microorganisms9112225 (PMC8621525; doi:10.3390/microorganisms9112225)
Supplement: Supplementary file 1 [file microorganisms-09-02225-s001.zip › Table S3-S19. BGC prediction.pdf]

Table S3. BGC prediction by antiSMASH v.5 in *Streptomyces* sp. CS014. Clusters with similarity ≥85% with already known BGCs were highlighted in bold. BGCs curated by hand were numbered with a letter after the cluster number. BGCs shorter than 20Kb are marked with an asterisk.

Cluster	Type	Most similar known cluster	% similarity	MIBiG BGC-ID
1	NRPS	Friulimicin	27	BGC0000354
2	Bacteriocin	Tetronasin	3	BGC0000163
**3**	**NRPS-t1PKS**	**SGR_PTMs**	**100**	**BGC0001043**
4	Terpene	Hopene	69	BGC0000663
**5**	**NRPS**	**Holomycin**	**92**	**BGC0000373**
6	Bacteriocin	-	-	-
**7**	**NRPS-t1PKS**	**Collismycin A**	**92**	**BGC0000973**
8	t1PKS	Microansamycin	32	BGC0001666
**9**	**Oligosaccharide-t1PKS**	**Sipanmycin**	**100**	**BGC0001459**
10	Siderophore	-	-	-
11	Terpene	-	-	-
**12**	**Lanthipeptide**	**AmfS**	**100**	**BGC0000496**
13	t1PKS-NRPS	Enduracidin	10	BGC0000341
**14**	**Lassopeptide**	**Keywimysin**	**100**	**BGC0001634**
**15**	**t2PKS**	**Granaticin**	**89**	**BGC0000227**
**16**	**Siderophore**	**Desferrioxamine_B**	**100**	**BGC0000941**
17	Lanthipeptide	-	-	-
**18**	**Ectoine**	**Ectoine**	**100**	**BGC0000853**
19	Terpene	Steffimycin D	19	BGC0000273
**20**	**Terpene**	**Isorenieratene**	**87**	**BGC0000664**
21	t3PKS	Herboxidiene	6	BGC0001065
**22a**	**NRPS**	**Coelichelin**	**90**	**BGC0000325**
**22b**	**NRPS**	**Streptobactin**	**94**	**BGC0000368**
**23**	**Terpene**	**Geosmin**	**100**	**BGC0001181**
**24**	**Butyrolactone**	**SCB1 / SCB2 / SCB3**	**100**	**BGC0000849**
25	NRPS-like	Nocathiacin	4	BGC0000609
26	NRPS-t1PKS	Lipopeptide 8D1-1 / lipopeptide 8D1-2	15	BGC0001370
**27**	**Melanin**	**Melanin**	**100**	**BGC0000911**
28	LAP-Thiopeptide	Lactazole	22	BGC0000606
**29a**	**t3PKS**	**Alkylresorcinol**	**100**	**BGC0000282**
29b	NRPS	Tetronasin	11	BGC0000163
30	NRPS-t1PKS	Daptomycin	6	BGC0000336
31	NRPS-t1PKS	Marienosin	9	BGC0000091
32*	NRPS	Cadaside A / Cadaside B	14	BGC0001968

Table S4. BGC prediction by antiSMASH v.5 in *Streptomyces* sp. CS057. Clusters with similarity ≥85% with already known BGCs were highlighted in bold. BGCs curated by hand were numbered with a letter after the cluster number. BGCs shorter than 20Kb are marked with an asterisk.

Cluster	Type	Most similar known cluster	% similarity	MIBiG BGC-ID
1	NRPS-t1PKS	Kanamycin	2	BGC0000703
2a*	Lanthipeptide	-	-	-
**2b**	**NRPS-transAT-PKS**	**Cycloheximide / actiphenol**	**94**	**BGC0000175**
**3a**	**t3PKS**	**Alkylresorcinol**	**100**	**BGC0000282**
3b	NRPS	Gobichelin A / B	16	BGC0000366
**4**	**Melanin**	**Melanin**	**100**	**BGC0000911**
5	NRPS-t1PKS	Sceliphrolactam	8	BGC0001908
6	t1PKS	C-1027	17	BGC0001584
7	Bacteriocin	Tetronasin	3	BGC0000163
**8**	**NRPS-t1PKS**	**SGR_PTMs**	**100**	**BGC0001043**
9	NRPS	Nucleocidin	47	BGC0001387
10	Terpene	Hopene	69	BGC0000663
**11**	**Terpene**	**2-methylisoborneol**	**100**	**BGC0000658**
12	Bacteriocin	-	-	-
**13**	**Oligossacharide-t2PKS-NRPS**	**Warkmycin CS1 / CS2**	**97**	**BGC0001438**
14	Siderophore	Ficellomycin	3	BGC0001593
15	Terpene	-	-	-
**16**	**Lanthipeptide**	**AmfS**	**100**	**BGC0000496**
**17**	**Melanin**	**Melanin**	**100**	**BGC0000912**
18	Thiopeptide-LAP	Frigocyclinone	17	BGC0002028
19	NRPS	Enduracidin	8	BGC0000341
**20**	**Ectoine-Butyrolactone-Ladderane-Arylpolyene-NRPS-t1PKS**	**Skyllamycin**	**95**	**BGC0000429**
21	Ectoine	Ectoine	75	BGC0000853
**22**	**Siderophore**	**Desferrioxamine E**	**100**	**BGC0001478**
23	Lanthipeptide	-	-	-
24	Lanthipeptide-NRPS	CDA1b / CDA2a / CDA2b / CDA3a / CDA3b / CDA4a / CDA4b	7	BGC0000315
**25**	**Ectoine**	**Ectoine**	**100**	**BGC0000853**
26	Terpene	Steffimycin D	19	BGC0000273
27	NRPS	-	-	-
28	LAP-Thiopeptide	Quinichelins	11	BGC0001752
29	t3PKS	-	-	-
**30**	**NRPS**	**Streptobactin**	**94**	**BGC0000368**
**31**	**Terpene**	**Geosmin**	**100**	**BGC0001181**
**32**	**Butyrolactone**	**A-factor**	**100**	**BGC0000848**

Table S5. BGC prediction by antiSMASH v.5 in *Streptomyces* sp. CS065a. Clusters with similarity ≥85% with already known BGCs were highlighted in bold.

Cluster	Type	Most similar known cluster	% similarity	MIBiG BGC-ID
**1**	**t3PKS**	**Alkylresorcinol**	**100**	**BGC0000282**
**2**	**Melanin**	**Melanin**	**100**	**BGC0000911**
3	NRPS	Thiocoraline	21	BGC0000445
4	Bacteriocin	Tetronasin	3	BGC0000163
**5**	**t1PKS-NRPS**	**SGR_PTMs**	**100**	**BGC0001043**
6	NRPS	A201a	8	BGC0001138
7	terpene	Hopene	69	BGC0000663
8	NRPSlike-arylpolyene-ectoine	Kosinostatin	13	BGC0001073
9	NRPS	Asukamycin	12	BGC0000187
10	Bacteriocin	-	-	-
11	Siderophore	Ficellomycin	3	BGC0001593
12	Terpene	-	-	-
**13**	**Lanthipeptide**	**AmfS**	**100**	**BGC0000496**
**14**	**Lassopeptide**	**keywimysin**	**100**	**BGC0001634**
15	Nucleoside	Toyocamycin	30	BGC0000881
16	Betalactone	Divergolide A - D	6	BGC0001119
17	t1PKS-NRPS	Sceliphrolactam	60	BGC0001770
18	NRPS	Phosphonoglycans	3	BGC0000806
19	PKSlike-NRPS	Malacidin A / B	5	BGC0001448
20	Thiopeptide-LAP	-	-	-
**21**	**Siderophore**	**Desferrioxamin B**	**100**	**BGC0000941**
22	Lanthipeptide	-	-	-
**23**	**t2PKS-oligosaccharide**	**Chromomycin A3**	**100**	**BGC0000210**
**24**	**Ectoine**	**Ectoine**	**100**	**BGC0000853**
25	Terpene	Steffimycin D	19	BGC0000273
26	t3PKS	Herboxidiene	6	BGC0001065
27	NRPS	Coelichelin	81	BGC0000325
**28**	**NRPS**	**Streptobactin**	**88**	**BGC0000368**
**29**	**Terpene**	**Geosmin**	**100**	**BGC0001181**
**30**	**Butyrolactone**	**SCB1 / SCB2 / SCB3**	**100**	**BGC0000849**

Table S6. BGC prediction by antiSMASH v.5 in *Streptomyces* sp. CS081a. Clusters with similarity ≥85% with already known BGCs were highlighted in bold. BGCs curated by hand were numbered with a letter after the cluster number. BGCs shorter than 20Kb are marked with an asterisk.

Cluster	Type	Most similar known cluster	% similarity	MIBiG BGC-ID
1	Siderophore	Ficellomycin	3	BGC0001593
2	Bacteriocin	-	-	-
**3**	**Terpene**	**Isorenieratene**	**100**	**BGC0000664**
**4a**	**Butyrolactone**	**SCB1 / SCB2 / SCB3**	**100**	**BGC0000849**
4b	NRPS	Primycin	5	BGC0001447
**5**	**Oligossacharide-OtherKS-t2PKS**	**Cosmomycin D**	**97**	**BGC0001074**
6	Butyrolactone - Furan	Methylenomycin	9	BGC0000914
7	Terpene	Hopene	69	BGC0000663
8	Nucleoside	-	-	-
**9**	**Terpene**	**2-methylisoborneol**	**100**	**BGC0000658**
10	NRPS	Streptolydigin	13	BGC0001046
11*	Melanin	Melanin	28	BGC0000908
12	Terpene	Borrelidin	4	BGC0000031
**13**	**Lassopeptide**	**Albusnodin**	**100**	**BGC0002006**
**14**	**Melanin**	**Melanin**	**100**	**BGC0000910**
**15**	**Siderophore**	**Desferrioxamine B**	**100**	**BGC0000941**
16	Terpene	-	-	-
17	NRPS	Cadaside A / B	19	BGC0001968
**18**	**Ectoine**	**Ectoine**	**100**	**BGC0000853**
**19**	**Terpene**	**Geosmin**	**100**	**BGC0001181**
**20**	**NRPS**	**-**	**-**	**-**
**21a**	**NRPS**	**Albachelin**	**90**	**BGC0001211**
21b	NRPS	Paenibactin	83	BGC0000401
22a	Terpene	Salinomycin	12	BGC0000144
22b	t2PKS	Spore pigment	83	BGC0000271
23	t1PKS	Meoabyssomicin / abyssomicin	56	BGC0001694
24	Phenazine	Endophenazine A / B	44	BGC0001080
25	NRPS-t1PKS	Arsono-polyketides	16	BGC0001283

Table S7. BGC prediction by antiSMASH v.5 in *Streptomyces* sp. CS090a. Clusters with similarity ≥85% with already known BGCs were highlighted in bold. BGCs curated by hand were numbered with a letter after the cluster number. BGCs shorter than 20Kb are marked with an asterisk.

Cluster	Type	Most similar known cluster	% similarity	MIBiG BGC-ID
1a	t3PKS	Herboxidiene	6	BGC0001065
1b	NRPS-betalactone	UK-68,597	4	BGC0001178
**2a**	**Terpene**	**2-methylisoborneol**	**100**	**BGC0000658**
2b	NRPS	Xanthothricin	33	BGC0000929
3	Terpene	Steffimycin	19	BGC0000273
**4**	**Ectoine**	**Ectoine**	**100**	**BGC0000853**
5	t2PKS-Oligosaccharide	Salpromycin E	69	BGC0001384
6	Lanthipeptide	-	-	-
**7**	**Siderophore**	**Desferrioxamin B**	**100**	**BGC0000941**
8	NRPSlike	Bottromycin A2	39	BGC0000469
9	Thiopeptide-LAP	-	-	-
10	Melanin	Grixazone	61	BGC0000662
11	t1PKS	Lavendiol	32	BGC0001649
**12**	**Lassopeptide**	**keywimysin**	**100**	**BGC0001634**
13	Bacteriocin	-	-	-
**14**	**Lanthipeptide**	**AmfS**	**100**	**BGC0000496**
15	Terpene	-	-	-
16	Siderophore	Ficellomycin	3	BGC0001593
17	Bacteriocin	-	-	-
18*	Butyrolactone	-	-	-
19	Lanthipeptide	SF2575	4	BGC0000269
20	Terpene	Hopene	69	BGC0000663
**21**	**NRPS-t1PKS**	**SGR_PTMs**	**100**	**BGC0001043**
22	Bacteriocin	Tetronasin	3	BGC0000163
23	NRPS	Crochelin A	7	BGC0002001
24	NRPS	Valinomycin / montanastatin	56	BGC0001846
**25**	**Melanin**	**Melanin**	**100**	**BGC0000911**
**26a**	**t3PKS**	**Alkylresorcinol**	**100**	**BGC0000282**
26b	NRPS	Gobichelin A / B	22	BGC0000366
**27**	**Terpene**	**Isorenieratene**	**100**	**BGC0000664**
28	Thiopeptide-Lanthipeptide-NRPS-t1PKS	Lactazole	33	BGC0000606
29	LAP	C-1027	7	BGC0000965
**30**	**Butyrolactone**	**SCB1 / SCB2 / SCB3**	**100**	**BGC0000849**
**31**	**Terpene**	**Geosmin**	**100**	**BGC0001181**
32a	PKSlike-t1PKS-TransATPKS-NRPS	Cosmomycin D	5	**BGC0001074**
**32b**	**NRPS**	**Streptobactin**	**94**	**BGC0000368**
33a	NRPS	Coelichelin	81	BGC0000325
33b*	Melanin	Melanin	28	BGC0000908
34	t3PKS	Violapyrone B	28	BGC0001905
35	t3PKS	-	-	-
36	PKSlike	-	-	-

Table S8. BGC prediction by antiSMASH v.5 in *Streptomyces* sp. CS113. Clusters with similarity ≥85% with already known BGCs were highlighted in bold. BGCs curated by hand were numbered with a letter after the cluster number. BGCs shorter than 20Kb are marked with an asterisk.

Cluster	Type	Most similar knowncluster	% similarity	MIBiG BGC-ID
1	t1PKS-NRPS-other	Polyoxypeptin	37	BGC0001036
2	Other	-	-	-
**3**	**NRPS**	**Coelichelin**	**100**	**BGC0000325**
4	Bacteriocin	Informatipeptin	42	BGC0000518
5	t1PKS-PKS like	Arsono-polyketide	83	BGC0001283
**6**	**Terpene**	**Hopene**	**100**	**BGC0000663**
**7**	**Lanthipeptide**	**SapB**	**100**	**BGC0000551**
**8**	**Terpene**	**Isorenieratene**	**100**	**BGC0001456**
9	Siderophore	Enduracidin	6	BGC0000341
10	Indole	7-prenylisatin	80	BGC0001294
**11**	**Terpene**	**Geosmin**	**100**	**BGC0001181**
12*	Bacteriocin	-	-	-
**13**	**t1PKS-NRPS like**	**Undecylprodigiosin**	**95**	**BGC0001063**
14a*	Siderophore	-	-	-
14b	t2PKS	Hatomarubigin	75	BGC0000232
15	NRPS	BD-12	17	BGC0001379
16	t2PKS	Spore pigment	66	BGC0000271
**17**	**Terpene**	**Albaflavenone**	**100**	**BGC0000660**
**18**	**Siderophore**	**Desferrioxamine_B**	**100**	**BGC0000941**
**19**	**Melanin**	**Melanin**	**100**	**BGC0000910**
**20**	**Ectoine**	**Ectoine**	**100**	**BGC0000853**
**21a**	**t2PKS-OtherKS-Oligosaccharide**	**Antibiotic HKI 10311129 / antibiotic A2121-1 / cervimycin D / cervimycin C / cervimycin A**	**90**	**BGC0001439**
21b	PKS-like / Other	-	-	-
22	t3PKS	Herboxidiene	8	BGC0001065
23	NRPS	Paulomycin	7	BGC0001732
24	NRPS	Paenibactin	83	BGC0000401
25	Terpene	-	-	-
**26**	**Butyrolactone - t3PKS**	**Germicidin**	**100**	**BGC0001454**
27	Terpene	Carotenoid	45	BGC0000633
28	Indole	5-isoprenylindole-3-carboxylate β-D-glycosyl ester	33	BGC0001483
**29**	**NRPS**	**Coelibactin**	**100**	**BGC0000324**
30	HglE-KS-lanthipeptide	Azinomycin B	6	BGC0000960

Table S9. BGC prediction by antiSMASH v.5 in *Streptomyces* sp. CS131. Clusters with similarity ≥85% with already known BGCs were highlighted in bold. BGCs curated by hand were numbered with a letter after the cluster number. BGCs shorter than 20Kb are marked with an asterisk.

Cluster	Type	Most similar known cluster	% similarity	MIBiG BGC-ID
1	NRPS-like	Nocathiacin	4	BGC0000609
**2**	**Butyrolactone**	**SCB1 / SCB2 / SCB3**	**100**	**BGC0000849**
**3**	**Terpene**	**Geosmin**	**100**	**BGC0001181**
**4a**	**NRPS**	**Streptobactin**	**94**	**BGC0000368**
**4b**	**NRPS**	**Coelichelin**	**90**	**BGC0000325**
5	t3PKS	Herboxidiene	6	BGC0001065
**6**	**NRPS-other**	**Actinomycin D**	**89**	**BGC0000296**
**7**	**Terpene**	**Isorenieratene**	**87**	**BGC0000664**
8	NRPS	A83543A	8	BGC0000148
9	Terpene	Steffimycin	19	BGC0000273
**10**	**Ectoine**	**Ectoine**	**100**	**BGC0000853**
11	Lanthipeptide	-	-	-
**12**	**Siderophore**	**Desferrioxamine_B**	**100**	**BGC0000941**
13	LAP-Thiopeptide	-	-	-
14	NRPS-like	Anthracimycin	13	BGC0001300
15	NRPS	Phosphonoglycans	3	BGC0000806
16	Lassopeptide	-	-	-
17	Lanthipeptide	Chalcomycin	9	BGC0000035
18a	NRPS	RP-1776	10	BGC0000429
18b	Ladderane-Arylpolyene	WS9326	35	BGC0001297
**18c**	**Lassopeptide**	**Keywimysin**	**100**	**BGC0001634**
19	Lanthipeptide	Carbapenem MM4550	6	BGC0000842
20	NRPS-t1PKS	WS9326	7	BGC0001297
**21**	**Lanthipeptide**	**AmfS**	**100**	**BGC0000496**
22*	Ectoine	Showdomycin	35	BGC0001778
23	Terpene	-	-	-
24	Siderophore	-	-	-
25	Lanthipeptide	Griselimycin	15	BGC0001414
26	Bacteriocin	-	-	-
**27**	**NRPS**	**Holomycin**	**92**	**BGC0000373**
**28**	**Terpene**	**2-methylisoborneol**	**100**	**BGC0000658**
29	PKSlike-NRPS	Fluostatins M-Q	16	BGC0001596
30	Terpene	Hopene	69	BGC0000663
**31**	**NRPS-t1PKS**	**SGR_PTMs**	**100**	**BGC0001043**
32	Bacteriocin	Tetronasin	3	BGC0000163
**33a**	**Terpene**	**Isorenieratene**	**100**	**BGC0000664**
33b	t1PKS-NRPS	Valinomycin / montanastatin	13	BGC0001846
**34**	**Melanin**	**Melanin**	**100**	**BGC0000911**
35	Thiopeptide-LAP	Lactazole	33	BGC0000606
**36a**	**t3PKS**	**Alkylresorcinol**	**100**	**BGC0000282**
36b	NRPS	CDA	7	BGC0000315
37	NRPS-t1PKS	Daptomycin	6	BGC0000336
**38**	**t1PKS-oligosaccharide**	**Stambomycin**	**96**	**BGC0000151**
39	NRPS	Actinomycin	7	BGC0000296

Table S10. BGC prediction by antiSMASH v.5 in *Streptomyces* sp. CS147. Clusters with similarity ≥85% with already known BGCs were highlighted in bold. BGCs curated by hand were numbered with a letter after the cluster number.

Cluster	Type	Most similar known cluster	% similarity	MIBiG BGC-ID
1	NRPS-like	Lavendiol	6	BGC0001649
**2**	**Butyrolactone**	**SCB1 / SCB2 / SCB3**	**100**	**BGC0000849**
**3**	**Terpene**	**Geosmin**	**100**	**BGC0001181**
**4a**	**NRPS**	**Streptobactin**	**94**	**BGC0000368**
**4b**	**NRPS**	**Coelichelin**	**90**	**BGC0000325**
4c	NRPS	Gougerotin	13	BGC0001610
5	t3PKS	Herboxidiene	6	BGC0001065
6	Arylpolyene	Chloramphenicol	11	BGC0000893
**7**	**Terpene**	**Isorenieratene**	**87**	**BGC0000664**
8	Terpene	Steffimycin D	19	BGC0000273
**9**	**Ectoine**	**Ectoine**	**100**	**BGC0000853**
10	Lanthipeptide	-	-	-
**11**	**Siderophore**	**Desferrioxamine B**	**100**	**BGC0000941**
12	NRPS	Phosphonoglycans	3	BGC0000806
13a	Phosphonate	Ishigamide	11	BGC0001623
**13b**	**Lassopeptide**	**Keywimysin**	**100**	**BGC0001634**
14	Lanthipeptide	Kistamicin A	8	BGC0001635
**15**	**Ladderane-NRPS**	**Chlorattinimycin**	**100**	**BGC0002100**
**16**	**t1PKS-oligosaccharide**	**Vicenistatin**	**90**	**BGC0000167**
17	NRPS-t1PKS	WS9326	10	BGC0001297
**18**	**Lanthipeptide**	**AmfS**	**100**	**BGC0000496**
19	Terpene	-	-	-
20	Siderophore	-	-	-
21	Bacteriocin	-	-	-
**22**	**NRPS**	**Holomycin**	**92**	**BGC0000373**
**23**	**Terpene**	**2-methylisoborneol**	**100**	**BGC0000658**
24	Terpene	Hopene	69	BGC0000663
**25a**	**NRPS-t1PKS**	**SGR_PTMs**	**100**	**BGC0001043**
25b	Bacteriocin	Tetronasin	3	BGC0000163
**26a**	**Terpene**	**Isorenieratene**	**100**	**BGC0000664**
26b	t1PKS-NRPS	-	-	-
**27**	**Melanin**	**Melanin**	**100**	**BGC0000911**
28	Thiopeptide-LAP	Lactazole	33	BGC0000606
**29a**	**t3PKS**	**Alkylresorcinol**	**100**	**BGC0000282**
29b	NRPS	Tetronasin	11	BGC0000163
30	NRPS-t1PKS	Daptomycin	7	BGC0000336
31	Lassopeptide	Ansatrienin (Mycotrienin)	7	BGC0000957

Table S11. BGC prediction by antiSMASH v.5 in *Streptomyces* sp. CS0149. Clusters with similarity ≥85% with already known BGCs were highlighted in bold. BGCs curated by hand were numbered with a letter after the cluster number.

Cluster	Type	Most similar known cluster	% similarity	MIBiG BGC-ID
1	NRPSlike	Nocathiacin	4	BGC0000609
**2**	**Butyrolactone**	**SCB1 / SCB2 / SCB3**	**100**	**BGC0000849**
**3**	**Terpene**	**Geosmin**	**100**	**BGC0001181**
**4a**	**NRPS**	**Streptobactin**	**94**	**BGC0000368**
**4b**	**NRPS**	**Coelichelin**	**90**	**BGC0000325**
**5**	**Terpene**	**Isorenieratene**	**100**	**BGC0000664**
6	t3PKS	herboxidiene	6	BGC0001065
7	NRPS-t1PKS	Daptomycin	17	BGC0000336
8	Terpene	Steffimycin D	19	BGC0000273
**9**	**Ectoine**	**Ectoine**	**100**	**BGC0000853**
10	Lanthipeptide	-	-	-
**11**	**Siderophore**	**Desferrioxamine B**	**100**	**BGC0000941**
12	Betalactone-Furan-Butyrolactone	Asukamycin	27	BGC0000187
13	Oligosaccharide-LAP	Prejadomycin / rabelomycin / gaudimycin C / gaudimycin D / UWM6 / gaudimycin A	12	BGC0000262
**14**	**Lassopeptide**	**Keywimysin**	**100**	**BGC0001634**
15	NRPS-t1PKS	Leinamycin	4	BGC0001101
**16**	**Lanthipeptide**	**AmfS**	**100**	**BGC0000496**
17	NRPS	Friulimicin A/ B/ C/ D	48	BGC0000354
18	Terpene	-	-	-
19	Siderophore	-	-	-
**20**	**t1PKS-oligosaccharide**	**Sipanmycin**	**100**	**BGC0001452**
**21**	**NRPS-t1PKS**	**Collismycin A**	**85**	**BGC0000973**
22	Bacteriocin	-	-	-
**23**	**NRPS**	**Holomycin**	**92**	**BGC0000373**
24	PKSlike-NRPS	Fluostatins M-Q	16	BGC0001596
25	Terpene	Hopene	69	BGC0000663
**26a**	**NRPS-t1PKS**	**SGR_PTMs**	**100**	**BGC0001043**
26b	Bacteriocin	Tetronasin	3	BGC0000163
27	t1PKS-NRPS	-	-	-
**28**	**Melanin**	**Melanin**	**100**	**BGC0000911**
29	Thiopeptide-LAP	Lactozole	33	BGC0000606
30	t3PKS	Tetronasin	11	BGC0000163
31	NRPS-t1PKS	Daptomycin	6	BGC0000336

Table S12. BGC prediction by antiSMASH v.5 in *Streptomyces* sp. CS159. Clusters with similarity ≥85% with already known BGCs were highlighted in bold. BGCs curated by hand were numbered with a letter after the cluster number. BGCs shorter than 20Kb are marked with an asterisk.

Cluster	Type	Most similar known cluster	% similarity	MIBiG BGC-ID
**1**	**NRPS**	**Coelichelin**	**100**	**BGC0000325**
2	Bateriocin	Informatipeptin	42	BGC0000518
3	t1PKS-PKSlike	Arsono-polyketides	83	BGC0001283
**4**	**Terpene**	**Hopene**	**100**	**BGC0000663**
5	NRPS-transatPKS	Phthoxazolin	20	BGC0001740
**6**	**Terpene**	**Isorenieratene**	**100**	**BGC0000229**
7a	NRPS-betalactone	Paulomycin	13	BGC0001732
7b*	Siderophore	-	-	-
8	Betalactone	Hormaomycins	13	BGC0000374
**9**	**Terpene**	**Geosmin**	**100**	**BGC0001181**
10	Bacteriocin	-	-	-
**11a**	**NRPS**	**Coelibactin**	**100**	**BGC0000324**
**11b**	**t1PKS-NRPSlike**	**Undecylprodigiosin**	**95**	**BGC0001063**
12	Siderophore	Ficellomycin	3	BGC0001593
13	t2PKS	Spore pigment	66	BGC0000271
**14**	**Terpene**	**Albaflavenone**	**100**	**BGC0001660**
15	Other	Ulleungmycin	16	BGC0001814
**16**	**NRPS**	**Sarpeptin A / sarpeptin B**	**100**	**BGC0001984**
17	t2PKS	Allocyclinone	24	BGC0001500
**18**	**Siderophore**	**Desferrioxamine B**	**100**	**BGC0000940**
19	Melanin	Melanin	60	BGC0000909
**20**	**Ectoine**	**Ectoine**	**100**	**BGC0001853**
21	t3PKS	Herboxidiene	8	BGC0001065
**22**	**t3PKS**	**Germicidin**	**100**	**BGC0001454**
23	Terpene	Carotenoid	54	BGC0000633
24	Índole	5-isoprenylindole-3-carboxylate β-D-glycosyl ester	33	BGC0001483
**25**	**Terpene**	**2-methylisoborneol**	**100**	**BGC0000658**
26	t2PKS	Medermycin	11	BGC0000245
27*	Butyrolactone	Coelimycin P1	16	BGC0000038
28	Other	Nenestatin	45	BGC0001693

Table S13. BGC prediction by antiSMASH v.5 in *Streptomyces* sp. CS207. Clusters with similarity ≥85% with already known BGCs were highlighted in bold. BGCs curated by hand were numbered with a letter after the cluster number. BGCs shorter than 20Kb are marked with an asterisk.

Cluster	Type	Most similar known cluster	% similarity	MIBiG BGC-ID
**1**	**Terpene**	**Albaflavenone**	**100**	**BGC0000660**
2	t2PKS	Spore pigment	66	BGC0000271
**3**	**Siderophore**	**Desferrioxamin_B**	**100**	**BGC0000941**
**4**	**Melanin**	**Melanin**	**100**	**BGC0000910**
**5**	**Ectoine**	**Ectoine**	**100**	**BGC0000853**
6	t3PKS	Herboxidiene	8	BGC0001065
**7**	**NRPS**	**Streptothricin**	**100**	**BGC0000432**
8	Terpene	Isorenieratene	63	BGC0001456
9	Índole	5-isoprenylindole-3-carboxylate β-D-glycosyl ester	33	BGC0001483
10	Terpene	Arylomycin	22	BGC0000306
**11**	**Índole**	**7-prenylisatin**	**100**	**BGC0001294**
12a*	Butyrolactone	Merochlorin	19	BGC0001083
12b	t2PKS	Fluostatin M_Q	67	BGC0001596
**13**	**Terpene**	**Hopene**	**92**	**BGC0000663**
14	NRPS	CDA1b / CDA2a / CDA2b / CDA3a / CDA3b / CDA4a / CDA4b	72	BGC0000315
15	Siderophore	Paulomycin	9	BGC0001732
**16**	**Terpene**	**Geosmin**	**100**	**BGC0001181**
17	Bacteriocin	-	-	-
18	Siderophore	-	-	-
19	NRPS-like	Alanylclavam / 2-hydroxymethylclavam / 2-formyloxymethylclavam / clavam-2-carboxylate	12	BGC0000843
20	Lanthipeptide	SapB	75	BGC0000551
21	NRPS	Ansamitocin P-3	7	BGC0001511

Table S14. BGC prediction by antiSMASH v.5 in *Streptomyces* sp. CS227. Clusters with similarity ≥85% with already known BGCs were highlighted in bold. BGCs curated by hand were numbered with a letter after the cluster number. BGCs shorter than 20Kb are marked with an asterisk.

Cluster	Type	Most similar known cluster	% similarity	MIBiG BGC-ID
1	NRPS	Cadaside A / B	19	BGC0001968
2	Terpene-NRPS	Butyrolactol A	20	BGC0001537
3	NRPS	Leinamycin	18	BGC0001101
**4**	**NRPS-t1PKS**	**SGR_PTMs**	**100**	**BGC0000663**
5	Terpene	Hopene	76	BGC0000663
6	Bacteriocin	-	-	-
7	Bacteriocin	-	-	-
8	Terpene-t1PKS	Tetronasion	9	BGC0000163
9	Siderophore	Ficellomycin	5	BGC0001593
**10**	**Terpene**	**Geosmin**	**100**	**BGC0001181**
**11**	**Terpene**	**Albaflavenone**	**100**	**BGC0000660**
12	Thiopeptide-LAP	Fluostatins M-Q	4	BGC0001596
13	Bacteriocin	-	-	-
**14**	**Lanthipeptide**	**SAL-2242**	**100**	**BGC0000546**
15	NRPS	Dechlorocuracomycin	16	BGC0001569
**16**	**NRPS**	**Surugamide A / D**	**100**	**BGC0001792**
**17**	**Siderophore**	**Desferroxiamine B**	**100**	**BGC0000941**
**18**	**Ectoine**	**Ectoine**	**100**	**BGC0000853**
**19**	**Bacteriocin-Terpene**	**Isorenieratene**	**85**	**BGC0000664**
20a	t3PKS	Herboxidiene	13	BGC0001065
**20b**	**t1PKS-NRPS**	**Candicidin**	**100**	**BGC0000034**
**20c**	**t1PKS-NRPS**	**Antimycin**	**100**	**BGC0000958**
20d	Lanthipeptide-t1PKS-NRPS	Thiolactomycin	60	BGC0001237
21	t1PKS-oligosaccharide	Stambomycin	68	BGC0000151
22*	Butyrolactone	-	-	-

Table S15. BGC prediction by antiSMASH v.5 in *Streptomyces coelicolor* A3(2). Clusters with similarity ≥85% with already known BGCs were highlighted in bold. BGCs curated by hand were numbered with a letter after the cluster number.

Cluster	Type	Most similar known cluster	% similarity	MIBiG BGC-ID
1	t1PKS-hglE-KS	Leinamycin	2	BGC0001101
**2**	**Terpene**	**Isorenieratene**	**100**	**BGC0000664**
3	Lanthipeptide	-	-	-
**4**	**NRPS**	**Coelichelin**	**100**	**BGC0000325**
5	Bacteriocin	Informatipeptin	42	BGC0000518
6	t3PKS	Herboxidiene	8	BGC0001065
**7**	**Ectoine**	**Ectoine**	**100**	**BGC0000853**
**8**	**Melanin**	**Melanin**	**100**	**BGC0000910**
**9**	**Siderophore**	**Desferrioxamine B**	**100**	**BGC0000941**
**10**	**NRPS**	**CDA1b / CDA2a / CDA2b / CDA3a / CDA3b / CDA4a / CDA4b**	**87**	**BGC0000315**
**11**	**t2PKS**	**Actinorhodin**	**100**	**BGC0000194**
**12**	**Terpene**	**Albaflavenone**	**100**	**BGC0000660**
13	t2PKS	Spore pigment	66	BGC0000271
14	Siderophore	-	-	-
**15**	**t1PKS-NRPSlike**	**Undecylprodigiosin**	**100**	**BGC0001063**
16	Bacteriocin	-	-	-
**17**	**Terpene**	**Geosmin**	**100**	**BGC0001181**
18	Siderophore	Enduracidin	6	BGC0000341
**20**	**Butyrolactone-t1PKS**	**Coelimycin P1**	**100**	**BGC0000038**
21	NRPS	Nogalamycin	40	BGC0000249
**22**	**Lanthipeptide**	**SapB**	**100**	**BGC0000551**
**23**	**Terpene**	**Hopene**	**100**	**BGC0000663**
**24**	**t1PKS-PKSlike**	**Arsono-polyketides**	**100**	**BGC0001283**
25	Lanthipeptide	-	-	-
**26**	**Other-t3PKS**	**Germicidin**	**100**	**BGC0001454**
27	Índole	5-isoprenylindole-3-carboxylate β-D-glycosyl ester	28	BGC0001483
**28a**	**t3PKS**	**Alkylresorcinol**	**100**	**BGC0000282**
**28b**	**NRPS**	**Coelibactin**	**100**	**BGC0000324**
**28c**	**Terpene**	**2-methylisoborneol**	**100**	**BGC0000658**
SCP1 plasmid				
29	Terpene	Lavendiol	6	BGC0001649
**30**	**Furan-butyrolactone**	**Methylenomycin A**	**100**	**BGC0000914**

Table S16. BGC prediction by antiSMASH v.5 in *Streptomyces avermitilis* MA-4680. Clusters with similarity ≥85% with already known BGCs were highlighted in bold. BGCs curated by hand were numbered with a letter after the cluster number.

Cluster	Type	Most similar known cluster	% similarity	MIBiG BGC-ID
**1**	**Terpene**	**Avermitilol**	**100**	**BGC0000683**
2	Lassopeptide	Moomysin	75	BGC0001673
**3**	**t1PKS**	**Filipin**	**100**	**BGC0000059**
4	NRPS	Diisonitrile antibiotic SF2768	66	BGC0001574
5	NRPS-PKSlike-T1PKS	Landepoxcin	11	BGC0001202
**6**	**t1PKS**	**LL-F28249α**	**100**	**BGC0000109**
**7**	**Terpene**	**Carotenoid**	**100**	**BGC0000633**
**8**	**Melanin**	**Melanin**	**100**	**BGC0000908**
9	NRPS-t1PKS	Foxicins A-D	12	BGC0001598
**10**	**Terpene**	**Hopene**	**92**	**BGC0000663**
11	Siderophore	Grincamycin	8	BGC0000229
**12**	**Terpene**	**Geosmin**	**100**	**BGC0001181**
13	Lassopeptide	Ashimides	8	BGC0001961
14	Bacteriocin	-	-	-
15	Butyrolactone-PKSlike-t1PKS-other	Antibiotic HKI 10311129 / antibiotic A2121-1 / cervimycin D / C / A	6	BGC0001439
16	PKSlike-t1PKS-t2PKS	Auroramycin	19	BGC0001522
17	Siderophore	-	-	-
18a	t2PKS	-	-	-
**18b**	**t1PKS**	**Oligomycin**	**100**	**BGC0000117**
**19**	**Terpene**	**Pentalenolactone**	**100**	**BGC0000678**
**20**	**Terpene**	**Albaflavenone**	**100**	**BGC0000660**
21	NRPS	Gobichelin A / B	16	BGC0000366
22	NRPS	WS9326	7	BGC0001297
23	PKSlike-Butyrolactone	5-isoprenylindole-3-carboxylate β-D-glycosyl ester	42	BGC0001483
24	NRPS-arylpolyene-ladderane	Atratumycin	28	BGC0001975
25	Terpene	-	-	-
**26**	**Siderophore**	**Desferrioxamin B**	**100**	**BGC0000941**
27	Melanin	Melanin	80	BGC0000909
**28**	**Lassopeptide**	**Citrulassin D**	**100**	**BGC0001550**
29	NRPS	SCO-2138	50	BGC0000595
**30**	**Ectoine**	**Ectoine**	**100**	**BGC0000853**
31	NRPS	Fogacin A / B / C	5	BGC0002021
32	t3PKS	Herboxidiene	8	BGC0001065
33	t1PKS	Kirromycin	5	BGC0001070
34	Siderophore	-	-	-
35	T1PKS-hglE-KS	Herboxidiene	10	BGC0001065
**36**	**Bacteriocin-lanthipeptide**	**Informatipeptin**	**100**	**BGC0000518**

Table S17. BGC prediction by antiSMASH v.5 in *Streptomyces clavuligerus* ATCC 27064. Clusters with similarity ≥85% with already known BGCs were highlighted in bold. BGCs curated by hand were numbered with a letter after the cluster number.

Cluster	Type	Most similar known cluster	% similarity	MIBiG BGC-ID
1	t1PKS	JBIR-100	72	BGC0001348
2	**Terpene**	**Geosmin**	**100**	**BGC0001181**
**3**	LAP-PKSlike-t1PKS-butyrolactone	4-hexadecanoyl-3-hydroxy-2-(hydroxymethyl)-2H-furan-5-one	63	BGC0000140
4	**Ectoine**	**Ectoine**	**100**	**BGC0000853**
**5**	**Siderophore**	**Desferrioxamin B**	**100**	**BGC0000941**
6	NRPS	-	-	-
7	Butyrolactone	Lactonamycin	3	BGC0000238
8	Lanthipeptide	-	-	-
9	Blactam	Alanylclavam / 2-hydroxymethylclavam / 2-formyloxymethylclavam / clavam-2-carboxylate / clavulanic acid	75	BGC0000843
**10**	**Melanin**	**Melanin**	**100**	**BGC0000911**
11	NRPS-blactam	Clavulanic acid / cephamycin C	58 / 78	BGC0000845 BGC0000319
**12**	**Nucleoside**	**Tunicamycin B1**	**85**	**BGC0000880**
13	Lanthipeptide	-	-	-
14	t1PKS-NRPS	Kanamycin	1	BGC0000703
15	Siderophore	-	-	-
16	NRPS	A-201A	15	BGC0000873
17	Bacteriocin	-	-	-
18a	t2PKS	Spore pigment	83	BGC0000271
18b	Lanthipeptide	SapB	75	BGC0000551
19	NRPS	-	-	-
20	Terpene	Hopene	69	BGC0000663
**21**	**NRPS**	**Holomycin**	**100**	**BGC0000373**
22	NRPS	Nucleocidin	47	BGC0001387
**23**	**t3PKS**	**Naringenin**	**100**	**BGC0001310**
24	NRPS-T1PKS-other-terpene	SGR PTMs	83	BGC0001043
25	siderophore	-	-	-
PSCL4 plasmid				
26	Terpene	(+)-T-muurolol	80	BGC0000675
27a	NRPS	Pyrronazol B	22	BGC0001750
**27b**	**terpene**	**(-)-δ-cadinene**	**100**	**BGC0000674**
28	Lassopeptide	-	-	-
29	Terpene	-	-	-
30	NRPSlike-indole-terpene	Abyssomicins	9	BGC0001492
31	Lanthipeptide-terpene	Venezuelin	75	BGC0000563
32	Terpene	-	-	-
33	Butyrolactone	-	-	-

34	NRPS-t1PKS-amglyccycl-terpene	Daptomycin	7	BGC0000336
**35**	**Blactam**	**Alanylclavam / 2-hydroxymethylclavam / 2-formyloxymethylclavam / clavam-2-carboxylate / clavulanic acid**	**100**	**BGC0000841**
**36**	**Índole**	**Staurosporine**	**93**	**BGC0000825**
37	Bacteriocin	-	-	-
38	Terpene	Primycin	5	BGC0001447
39	Melanin-t1PKS	Neocarzinostatin	15	BGC0000112
40	NRPS,NRPS-like -t1PKS-Ectoine-Phosphoglycolipid	Maduropeptin	30	BGC0001008
41	Terpene-NRPS	Rapamycin	7	BGC0001040
42	NRPS-like	Indigoidine	40	BGC0000375

Table S18. BGC prediction by antiSMASH v.5 in *Streptomyces albidoflavus* J1074. Clusters with similarity ≥85% with already known BGCs were highlighted in bold. BGCs curated by hand were numbered with a letter after the cluster number.

Cluster	Type	Most similar known cluster	% similarity	MIBiG BGC-ID
1	t1PKS-NRPS	-	-	-
**2**	**t1PKS-NRPS**	**SGR PTMs**	**100**	**BGC0001043**
3	terpene	Hopene	76	BGC0000663
4	PKSlike-oligosaccharide	Paulomycin	66	BGC0001731
5	Bacteriocin	-	-	-
6	NRPS	Dechlorocuracomycin	16	BGC0001569
7	Siderophore	Ficellomycin	5	BGC0001593
**8**	**Terpene**	**Geosmin**	**100**	**BGC0001181**
**9**	**Terpene**	**Albaflavenone**	**100**	**BGC0000660**
10	LAP-thiopeptide	Fluostatins M-Q	4	BGC0001596
11	Bacteriocin	Goadsporin	12	BGC0000565
**12**	**Lanthipeptide**	**SAL-2242**	**100**	**BGC0000546**
13	NRPS	WS9326	7	BGC0001297
**14**	**NRPS**	**Surugamide A / D**	**100**	**BGC0001792**
15	NRPS	-	-	-
**16**	**Siderophore**	**Desferrioxamin B**	**100**	**BGC0000941**
**17**	**Ectoine**	**Ectoine**	**100**	**BGC0000853**
18	NRPSlike	Indigoidine	80	BGC0000375
19a	Bacteriocin	-	-	-
**19b**	**Terpene**	**Isorenieratene**	**85**	**BGC0000664**
20	t3PKS	Herboxidiene	12	BGC0001065
**22**	**t1PKS-NRPSlike**	**Candicidin**	**100**	**BGC0000034**
**23**	**NRPS-NRPSlike-t1PKS-lanthipeptide**	**Antimycin**	**100**	**BGC0000958**

Table S19. BGC prediction by antiSMASH v.5 in *Pseudonocardia* sp. Ae150A_Ps1. Clusters with similarity ≥85% with already known BGCs were highlighted in bold.

Cluster	Type	Most similar known cluster	% similarity	MIBiG BGC-ID
1	t1PKS	Nystatin-like *Pseudonocardia* polyene	78	BGC0000116
2	Bacteriocin-NRPSlike	Streptobactin	11	BGC0000368
3	t1PKS	Nystatin-like Pseudonocardia polyene	47	BGC0000116
4	Terpene	SF2575	6	BGC0000269
5	NRPS	Scabichelin	40	BGC0000423
6	Betalactone	Cmc-thuggacin A / Cmc-thuggacin B	12	BGC0001342
7	Bacteriocin	-	-	-
8	NRPSlike	Stenothricin	13	BGC0000431
9	NRPS	Mirubactin	42	BGC0000392
**10**	**Ectoine**	**Ectoine**	**100**	**BGC0000853**
11	Oligosaccharide	-	-	-
12	Other	Thiocoraline	5	BGC0000445
13	Lanthipeptide	Kanamycin	1	BGC0000703
14	NRPSlike	Tomaymycin	11	BGC0000448
15	NRPS	Mirubactin	21	BGC0000392
16	Terpene	Isorenieratene	28	BGC0000664
