# Supplementary figures and images for: A Multidisciplinary Approach to Unraveling the Natural Product Biosynthetic Potential of a Streptomyces Strain Collection Isolated from Leaf-Cutting Ants
Source: Microorganisms. 2021 Oct 26;9(11):2225. doi: 10.3390/microorganisms9112225 (PMC8621525; doi:10.3390/microorganisms9112225)

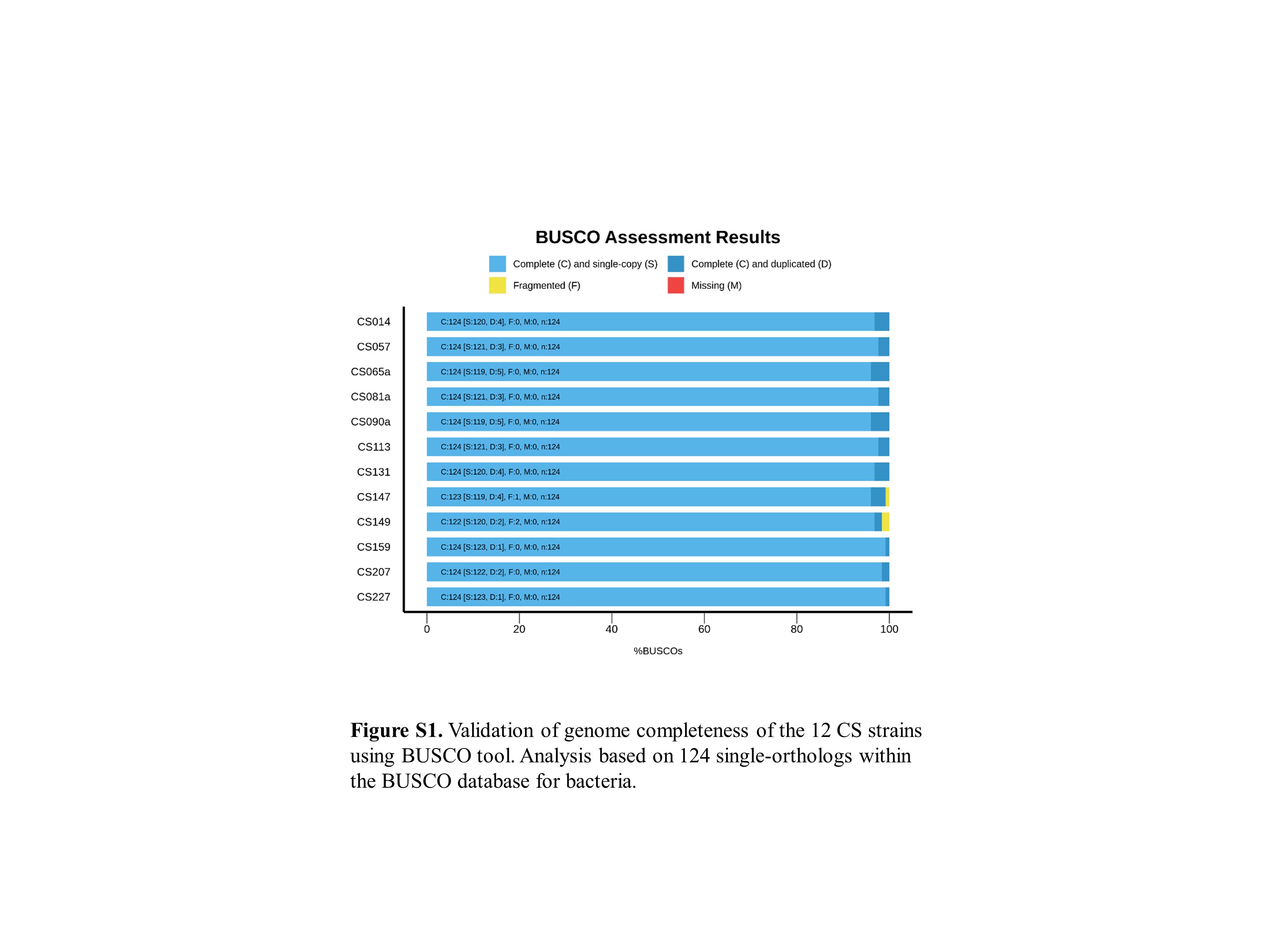

Supplement: Supplementary file 1 [file microorganisms-09-02225-s001.zip › Figure. S1. BUSCO.jpg]
